# Biobank-scale methods and projections for sparse polygenic prediction from machine learning

**DOI:** 10.1038/s41598-023-37580-5

**Published:** 2023-07-19

**Authors:** Timothy G. Raben, Louis Lello, Erik Widen, Stephen D. H. Hsu

**Affiliations:** 1 Department of Physics and Astronomy, Michigan State University, Michigan, USA; 2 Genomic Prediction, Inc., North Brunswick, NJ USA

**Keywords:** Genomics, Quantitative trait, Heritable quantitative trait

## Abstract

In this paper we characterize the performance of linear models trained via widely-used *sparse* machine learning algorithms. We build polygenic scores and examine performance as a function of training set size, genetic ancestral background, and training method. We show that predictor performance is most strongly dependent on size of training data, with smaller gains from algorithmic improvements. We find that LASSO generally performs as well as the best methods, judged by a variety of metrics. We also investigate performance characteristics of predictors trained on one genetic ancestry group when applied to another. Using LASSO, we develop a novel method for projecting AUC and correlation as a function of data size (i.e., for new biobanks) and characterize the asymptotic limit of performance. Additionally, for LASSO (compressed sensing) we show that performance metrics and predictor sparsity are in agreement with theoretical predictions from the Donoho-Tanner phase transition. Specifically, a future predictor trained in the Taiwan Precision Medicine Initiative for asthma can achieve an AUC of $0.63_{(0.02)}$ and for height a correlation of $0.648_{(0.009)}$ for a Taiwanese population. This is above the measured values of $0.61_{(0.01)}$ and $0.631_{(0.008)}$, respectively, for UK Biobank trained predictors applied to a European population.

## Introduction

Given the complexity of the human genome, large datasets are required to detect associations between specific genetic variations and their effect on phenotypes. These large datasets provide the statistical power necessary to overcome false signals (fluctuations) resulting from examination of millions of genetic variants at a time. With the advent of very large biobanks^1–3^, which collect millions of individual genotypes and associated phenotypes, it has become possible to probe the genetic architectures of important disease risks and other complex traits.

The analysis of large genotype and phenotype datasets has led to the development of polygenic scores (PGS). A PGS is simply a score built from numerically weighting the state of a persons genome. In most work to date, and in this paper, we are interested in linear PGS built from single nucleotide polymorphisms (SNPs), i.e., $PGS = \sum \bar{X}\cdot \vec {\beta }$, for genotype matrix $\bar{X}$ and SNP weights $\vec {\beta }$. SNP weights are typically obtained through a machine learning algorithm on genotype/phenotype pairs and can be as simple as single marker regression (e.g., Genome-Wide-Association-Studies or GWAS).

The vast majority of available GWAS and biobank data is from individuals of European ancestry. For example, the UK Biobank^3^ (UKB) is more than $90\%$ self-reported white. As a consequence, current PGS perform better for descendants of Europeans. There are a number of new biobank-scale efforts, focusing on non-European populations, which will ameliorate this situation, e.g., the Taiwan Precision Medicine Initiative^4^ (TPMI). Other novel projects have focused on gathering samples with ancestral equity in mind, e.g., All of Us^5^ (AoU). However, until more diverse data become available, it is necessary to adapt the European results for other ancestral populations in order for PGS to have utility for the largest number of individuals possible—i.e., in applications such as disease risk estimates, potential clinical interventions, etc.

As genotype databases become larger and as sequencing technology incorporates more SNPs (i.e., increasing the number of SNPs through imputation, larger arrays, and whole genome sequencing or WGS), novel difficulties arise in PGS construction. First, larger samples and features require greater computational power (we comment on the computational requirements for the results in this paper in the Supplementary Information). Second, and as mentioned above, the application of PGS has been largely restricted to those of European ancestry^6–17^. In order to “transport” PGS to other ancestry groups, many techniques have been proposed, using features that are most important in different groups and adjusting their specific weights^15,18–22^ (e.g., by using minor allele frequency differences or functional information). The complexity of this analysis clearly scales with the number of relevant features. Third, future benefits of PGS^8,11,11,16,17,23–43^ rely on genotyping future participants. If this genotyping can be restricted to a small number of SNPs (e.g. as opposed to more costly WGS) it can be more cost effective to implement. Fourth, most PGS development approaches and methods use linear models. Further challenges include non-linear SNP effects (e.g., which are responsible for the difference between narrow and broad sense heritability^44–50^), the relationship between tagged vs causal SNPs, and incorporating genome-environment interactions. Addressing these challenges again scales with the number of relevant features.

In this paper we focus on the performance, detailed application, and future power of *sparse* algorithms, i.e. algorithms that perform feature selection, for the 11 traits listed in Table 1. Performing feature selection can help ameliorate some of the issues raised above. Sparse algorithms have previously been shown to be comparable to non-sparse methods in terms of standard metrics (e.g., area under receiver operator curve (AUC), correlation, $r^2$, etc.)^11,51–53^. We focus on 11 phenotypes that have been previously been shown to cover a wide range of sparsities (see Table 1): asthma, atrial fibrillation, breast cancer, coronary artery disease (CAD), hypertension, type 1 and type 2 diabetes (T1D, T2D), body mass index (BMI), direct bilirubin, height, and lipoprotein A.

As mentioned, there are many efforts to modify PGS trained in primarily European ancestries to improve performance in non-European ancestries. This is a complex endeavor that involves trait specific characteristics, allele frequency and effect size dependencies, LD structures, and more. (See for example^52,54,54–61^ for recent progress along these lines and^62^ for a review of outstanding challenges). As we discuss, these improvements, while important, are unlikely to close the gap completely. To directly close this gap, large data cohorts from or including diverse ancestry groups are needed to perform *de novo*, ancestry specific training. This is difficult and expensive. Hence it is valuable to understand in advance what the resulting benefits will be for polygenic prediction. In this work we present a novel method for projecting and predicting the results from sparse algorithms in a novel biobank.

The main results of this paper can be summarized as follows

1. Widely-used sparse methods perform comparably, with a simple LASSO-based approach regularly achieving the best results.
2. Increased biobank/database size and access to new datasets will lead to large gains, especially for the performance of PGS in diverse ancestries.
3. We develop a novel method which predicts correlation and AUC for continuous and case/control phenotypes (respectively), with uncertainty bands, for biobank-sized datasets. This new projection method can be used to guide biobank recruitment.
4. We explore details of “phase change” behavior of compressed sensing/LASSO with increasing data size, and the corresponding the SNP content of resulting predictors.

**Table 1:**
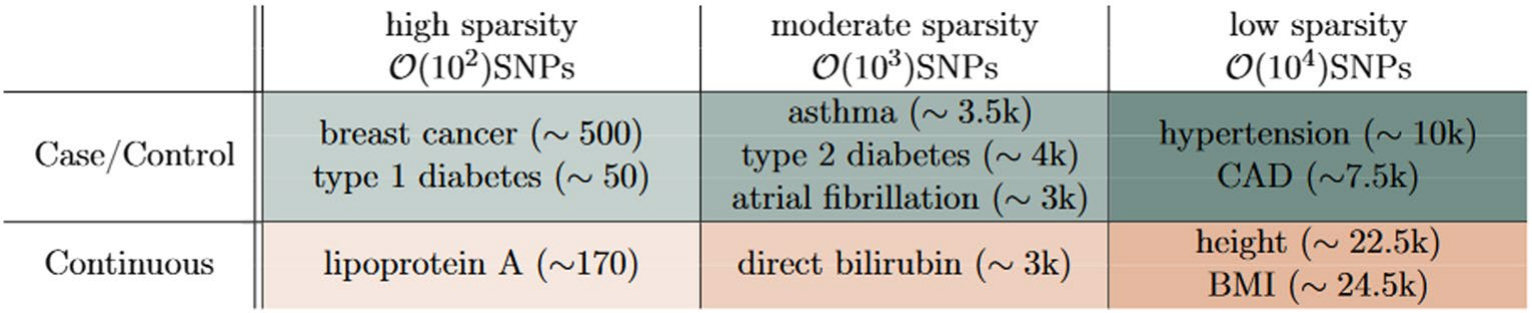
The 11 phenotypes studied in this work and their relative sparsity. As described in Section “Sparsity” predictor sparsity can be defined in a variety of ways. Here we color-code the various traits according to the order of magnitude of SNP sparsity. Numbers in parentheses are the approximate number of SNPs used in a LASSO trained predictor using the maximum amount of data from the UKB. This definition of sparsity is consistent with the number of SNPs with non-zero weights found in previous publications^11,12^.

## Results

Here we present the main results of this project. Additional details concerning specific methods are found in Section “Methods” and in the Supplementary Information. The main results are obtained using UKB genotype-phenotype data. Training with PRScs at times uses linkage disequilibrium (LD) information from the 1000 Genomes Project^1^ (1kg). Projections are given for de novo training in TPMI and AoU. We refer to ancestry groupings European (EUR), South Asian (SAS), East Asian (EAS), African (AFR), and American (AMR). These labeling conventions come from^63^.

### Comparison of sparse predictors

We compare the performance of several sparse methods: LASSO, Elastic Net, L1-penalized Logistic regression (for case-control conditions), and PRScs with LD matrix information from either UKB or 1KG. It is important to note that the results presented here for AUC and correlation are for *purely genetic* PGS. In brief, phenotypes are first regressed on covariates (such as age, sex, and the first 20 genetic principal components) – this allows them to explain as much of the variance as possible so that we can conservatively estimate purely genetic effects. Only then are SNP predictors are trained (using genotype-phenotype data or summary statistics in the case of PRScs) on residual phenotypes. Further details about the training and evaluation of PGS can be found in Section “Methods”.

**Figure 1:**
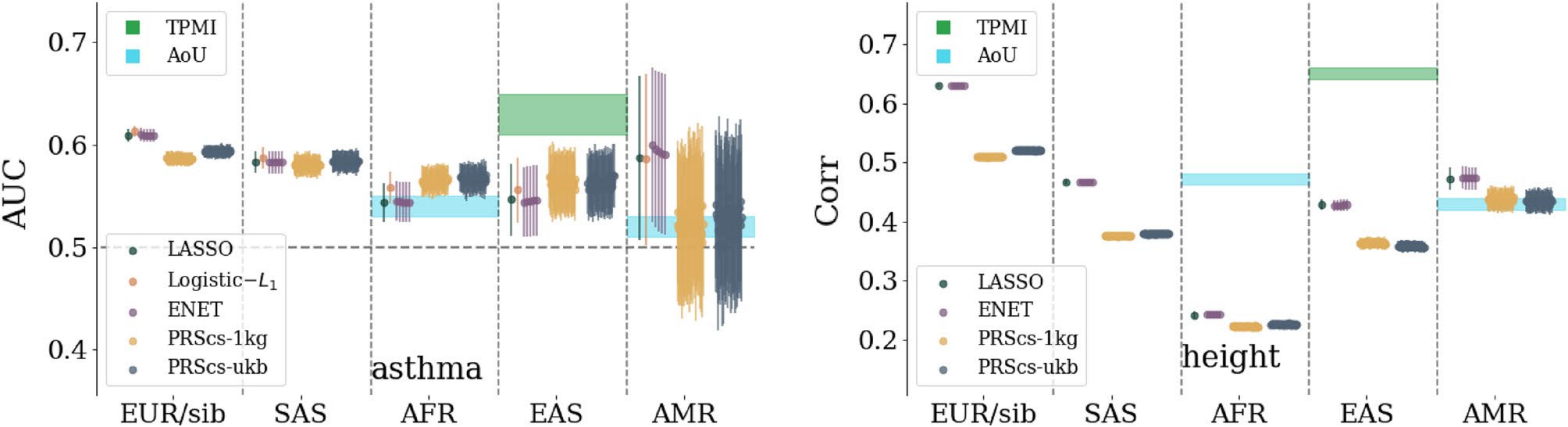
Comparison of sparse methods for asthma and height predictors with a comparison to prediction bands for more diverse biobanks. On the left, asthma predictors trained on a UKB white population. Predictors are built with LASSO, $L_1$-penalized Logistic regression, Elastic Nets, and PRScs with UKB and 1,000 Genomes LD matrices. The specific parameters for the Elastic nets and PRScs are described in Section “Methods”. Similar results for the other phenotypes can be found in the Supplementary Information.

In Fig. 1 we see the comparison results for asthma and height. Similar plots for the other phenotypes can be found in the Supplementary Information. Ancestry groups SAS, AFR, EAS, and EUR result from UKB definitions of self-reported ancestry (although training, as described in Section “Methods” involves a principal component, or PC, adjustment). AMR refers to an American-like group constructed via principal component clustering detailed in the Supplementary Information and similar to that found in^7^. Sib refers to a set of white siblings (i.e. every member of the set has at least one sibling also in the set) where the ancestry is self-reported, but the sibling status is determined by a genetic analysis as detailed in the Supplementary Information. This sib-set attempts to partially control for environmental effects as described in^64,65^. It also allows for performing sibling selection experiments as described in Section “Methods”. All results reflect training on a EUR population and then applied to a set of siblings or a different ancestry group, not used in training. The bands for TMPI/AoU are based on projections for future biobanks—this is described in detail in Section “Biobank projections”.

Uncertainty (error bars) depends on cross-validation (i.e. multiple training sets), finite size effects from computing AUC/correlation, and from sample sizes. Details about uncertainty calculations are given in the Supplementary Information. For case-control conditions this can lead to error bars that are the same size as the central values. However, for continuous phenotypes with much larger sample sizes this is not the case. For example, compare the AMR group on both plots in Fig. 1. On the left, the AUC error bar overlaps 0.5 (i.e. consistent with no signal), while on the right, the correlation error bar is relatively small.

Comparing performance across ancestry groups, we see a well known fall-off behavior which is observed when predictors trained in one population are applied to another (e.g., see^7,52^). The amount of fall-off is phenotype specific and ranges from complete fall-off, e.g. diabetes fall-off from sib to AFR, to negligible fall-off, e.g. breast cancer AUC from sibs to EAS. The relative order of fall-off is also phenotype specific. For most traits, the (EUR-ancestry) sibling set shows the least reduction—then either SAS, EAS, and AMR,—and finally AFR. However, there are clear exceptions like the direct bilirubin correlation which goes from largest metric to smallest: Sib, SAS, AFR, EAS, AMR. There have been recent arguments that PGS fall-off is roughly linear as a function of local genetic distance^52^. We should note that this claim is not necessarily in conflict with the results we present here. First, there are many exceptions to this general linear behavior, e.g., Figure 5 in^52^. Second, the claimed linear fall-off is a function of Euclidean distance in PC space of training population. For genetically distant groups, the axes of variation will be different: any measure of PC distance is therefore a *local* measure. In other words, only ancestry groups that are near-enough to the original population where PCs were computed can be considered well-ordered in terms of genetic difference. These effects can also be exacerbated by the fact that these are all *sparse* predictors and, after projecting onto PC space, the order of Euclidean genetic-distance may change.

**Figure 2:**
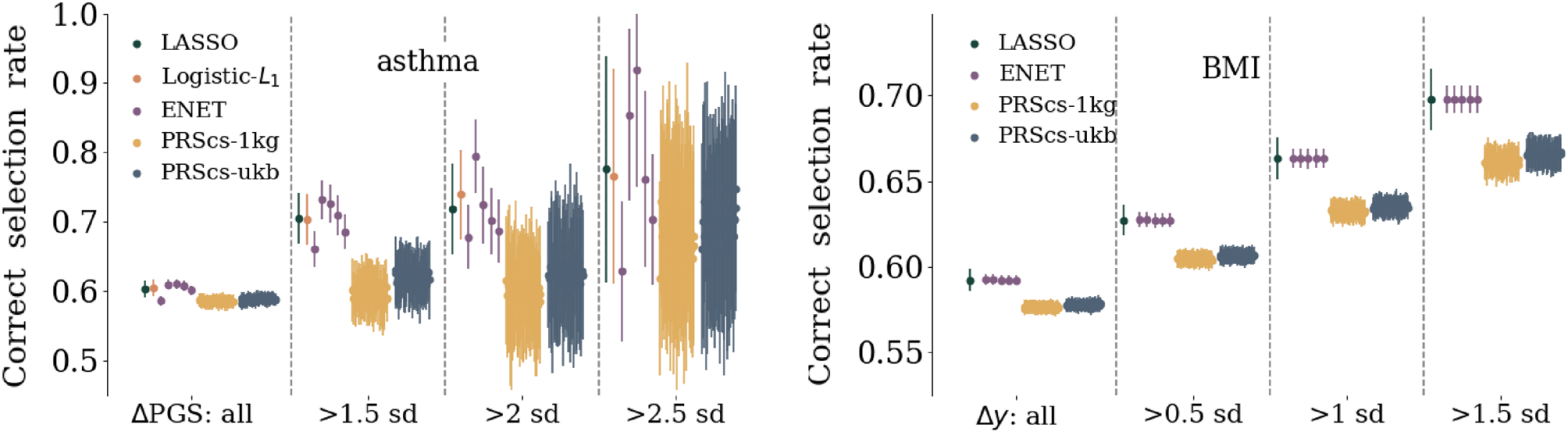
Left: affected sibling pair (ASP) selection rate for asthma. Pairs of siblings, where one person is a case and the other a control, are used and the rate corresponds to the number of times the case sibling has the higher PGS. The rate of correct selection, and uncertainty, increases if the siblings are also separated by at least 1.5, 2, or 2.5 standard deviations in PGS. Right: rank order selection rate for BMI respectively. The rate corresponds to frequency of the sibling with the larger BMI also having the larger PGS. Again the selection rate, and uncertainty (due to reduced statistics), increase if the sibling BMI is required to differ by at least 0.5, 1, or 1.5 standard deviations. Similar results for the other phenotypes are found in the Supplementary Information. These tests were developed in^64,65^. More detailed descriptions of these tests and how siblings are defined can be found in Section “Methods” and in the Supplementary Information.

The performance of PGS can always be confounded due to environmental factors, interactions between genes and the environment, and non-linear genetic effects (e.g., epistasis). To attempt to guard against some of these effects we can perform sibling tests similar to those described in^64,65^. Genetic siblings can be assumed to have, on average, a more similar environmental background than unrelated individuals. However, there can be a competing effect from the enhancement of signals from effects like genetic nurture^66^. For case control conditions we can create *affected sibling pairs* (ASPs) where one person is a case and one is a control. Then we can ask what fraction of the time does the higher PGS correspond to the case vs control. We can also condition this question on the PGS difference being larger than some cut-off (e.g., 1.5, 2, or 2.5 standard deviations). For continuous phenotypes we can simply compare the fraction of the time where the person with the higher PGS also has the higher phenotype value. We can again condition this question for siblings whose phenotypes are separated by a cut-off (e.g., 0.5, 1, or 1.5 standard deviations). These selection rates for asthma and BMI are shown in Fig. 2 as an example; the results for all traits considered in this work can be found in the Supplementary Information. Again, we see that while several methods are extremely competitive, LASSO is regularly among the best performing methods. For larger and larger cut-offs, the selection rate improves, but the associated uncertainty also increases largely because of decreasing sample sizes. Interestingly, while PRScs performed similarly to other methods in terms of AUC and correlation, it routinely underperforms methods training directly on genetic data in sibling selection tests.

**Figure 3:**
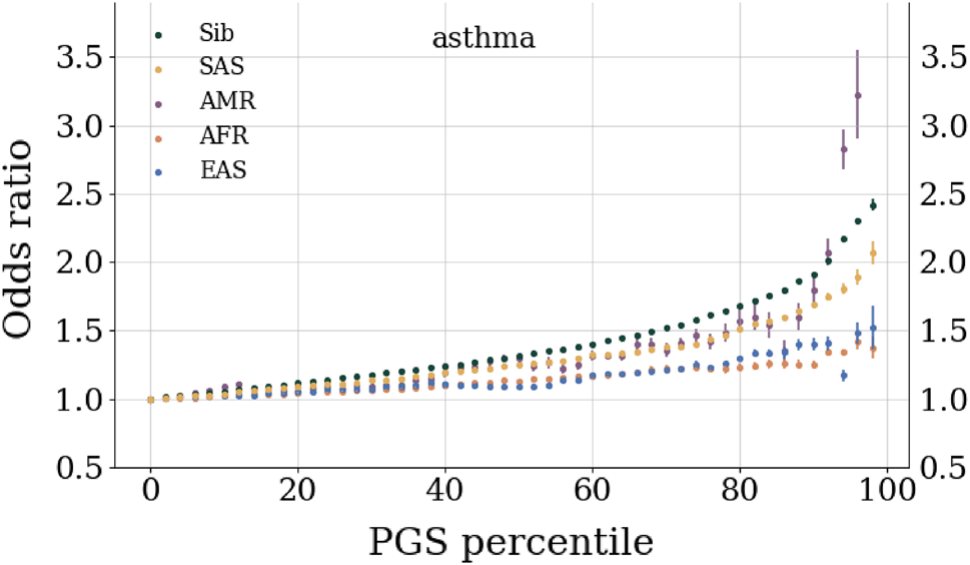
Inclusive odds ratio (OR) for asthma. The inclusive OR is the ratio of all cases to controls *at a given PGS or above* normalized to the ratio of the total number of cases to controls. At the highest PGS bins, data is omitted if there are no cases or controls. Similar plots for the other phenotypes and details about how uncertainties are computed are all located in the Supplementary Information.

PGS for case-control conditions can be converted to more clinically interpretable metrics. In Fig. 3 we see an example of an *inclusive* odds ratio (OR) for asthma. It is inclusive in the sense that the OR corresponds to the ratio of the cases to controls (normalized by the ratio of the total cases to controls) *at a specific PGS value or above*. As before uncertainties are conservative and include contributions from multiple cross-validation folds and finite size effects. Similar plots for the other case control conditions can be found in the Supplementary Information. Because LASSO routinely performed among the best predictors in terms of AUC and correlation we only present OR plots for this method. Analogous plots for the other methods can be generated similarly, although for all 7 case-control traits it leads to 357 plots which can be difficult to interpret. An initial interpretation of these results is that at extreme values of PGS there are large increases in OR: for asthma, within ancestry testing (i.e. the sibling group) leads to $2<\text {OR}<2.5$ at large PGS. When testing on other ancestries, an optimistic interpretation is that asthma OR ranges from 1.25 to 3.5 at large PGS. While this is encouraging, we also urge caution. The extremes of the PGS distributions are the regions where model assumptions are most likely to break down, e.g., the linearity of SNP effects. Additionally, the sample sizes in these regions are smallest which leads to large uncertainties and difficulty interpreting the results. In addition, odds ratios are difficult to model in the presence of non-Gaussian distributions. The inclusive odds ratio can be written as a ratio of cumulative distribution functions of cases and controls. Similarly the PGS percentile can be written as an integral over the sum of the probability distribution functions for cases and controls.

### Biobank projections

This section details how training at various sizes in current biobanks can be used to model the growth dependence on training size. For most clinically relevant metrics, e.g., correlation and AUC below, this growth can be modeled with uncertainty. Using relatively simple parametric functions, we find that we can model the growth of these metrics. The results can be used to guide future studies and genotype-phenotype database construction by identifying where large gains can be made.

For sparse methods like LASSO, it has been previously shown that using self-reported ancestry performs similarly to principal component based clustering when based on AUC and correlation metrics^11^. Nonetheless, after sorting on self-reported ancestry, we perform an additional regression on the top 20 PCs to adjust for any remaining population stratification.

To be conservative in the projections for PRS performance in other biobanks, several modeling assumptions are made. First, to be conservative we assume that these biobanks will have population prevalence rates for diseases even though some biobanks over-recruit cases to enrich their datasets. Additionally, the actual incidence rate for disease conditions fluctuates over time. For the conditions considered in this paper we try to consider the most recent surveys of all ages. Finally, when there are various estimates for disease prevalence within ancestry sub-groups, we choose a conservative (i.e. low) prevalence as the representative for the overall ancestry prevalence. Specific details about within ancestry prevalences are given in the Supplementary Information. Preliminary training using TPMI and the LASSO method described here was presented at ASHG 2022^67^ and is consistent with the projection function predictions.

**Figure 4:**
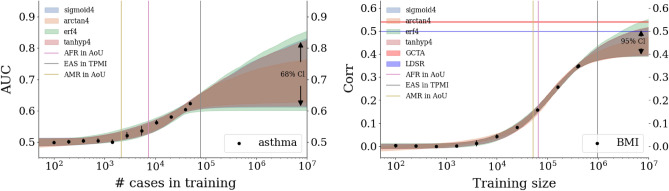
Growth of AUC (left: asthma) and correlation (right: BMI) as a function of training size in the UKB. Colored, curved bands come from fitting data with various 4 parameter functions. Width of the band corresponds to a confidence interval on the predictions: on the left 2 standard deviations or $\sim 68\%$ and on the right 4 standard deviations or $\sim 95\%$. Vertical bars represent projections for de novo training in other biobanks using literature prevalences, summarized in the Supplementary Information. If one assumes that a phenotype is determined by the sum of a genetic component and another *uncorrelated* random component (i.e., P = G + E), then the heritability is simply the square of the correlation between P and G. On the right, this apprximation is used to convert heritability predictions from GCTA and LDSR to horizontal correlation bands.

**Table 2:**
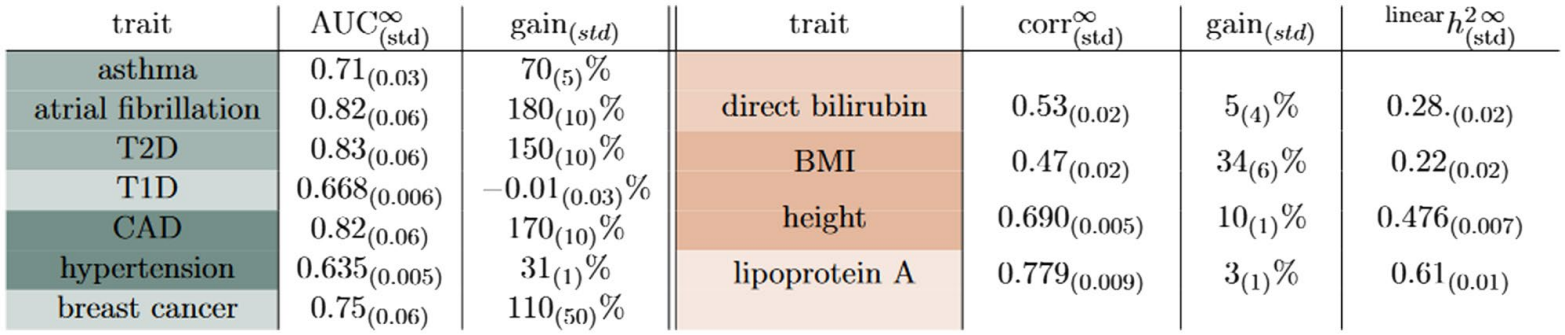
Asymptotic projections for AUC and and correlation for case-control and continuous traits respectively. Type 1/2 diabetes (T1/2D), coronary artery disease (CAD), and body mass index (BMI) are all abbreviated to save space. “Gain” represents the additional gain over the best result from training reported here. For most case control conditions, except T1D, there are large gains that can be found from increased training sizes. For continuous phenotypes, BMI can benefit from training on larger data sizes. Correlations are also translated to the asymptotic projection of linear, narrow-sense SNP heritability explained by the predictor. Colors correspond the relative sparsity of the predictor as mentioned in Table 1.

In Fig. 4 we see projection bands for asthma and BMI. The bands grow from no signal (0.5 AUC and 0 correlation respectively) to asymptotic values. The various colored prediction bands correspond to the Monte Carlo (MC) confidence intervals for the various fit functions as described in Section “Methods”. The asymptotic predictions for each trait can be averaged, incorporating the confidence intervals, and the results are given in Table 2. Further projection plots for the remaining traits can be found in the Supplementary Information.

The fraction of phenotypic variance captured by SNPs (i.e., the linear, narrow sense heritability) of traits is traditionally estimated via means such as Restricted Maximum Likelihood estimates (REML) and using Linkage Disequilibrium Score Regression (LDSR). For continuous traits, the correlation of the residual phenotype with the PGS can be related to the linear SNP heritability *explained by the predictor* by simply squaring the correlation. In Table 2 this can be seen in the final column. The heritability explained by the predictor is a *lower bound* on the REML heritability in that there may not be enough data to saturate the REML estimates. In Fig. 4 we can see the heritability estimates from GCTA (using REML) and LDSR converted to a correlation scale. To make this comparison, we assume that a phenotype is determined by the sum of a genetic component and another *uncorrelated* random component (i.e., P = G + E). In this case, the heritability is simply the square of the correlation between P and G. This allows a direct comparison of the new correlation results presented here and traditional methods like GCTA and LDSR. For traits like BMI and height it appears that, eventually, sparse predictors will capture all the linear SNP heritability. For much sparser conditions, e.g. Lipoprotein A presented in the Supplementary Information, it appears that sparse methods are out performing traditional measures of heritability.

### Sparse output interpretation

An advantage of sparsity is that, because there are fewer features, it is relatively easier to categorize features compared to non-sparse methods. Here we identify important features for predictors for each trait. Because a simple LASSO routinely performed as one of the best predictors in terms of AUC and correlation in Section “Comparison of sparse predictors” we focus here on interpreting the LASSO outputs.

**Figure 5:**
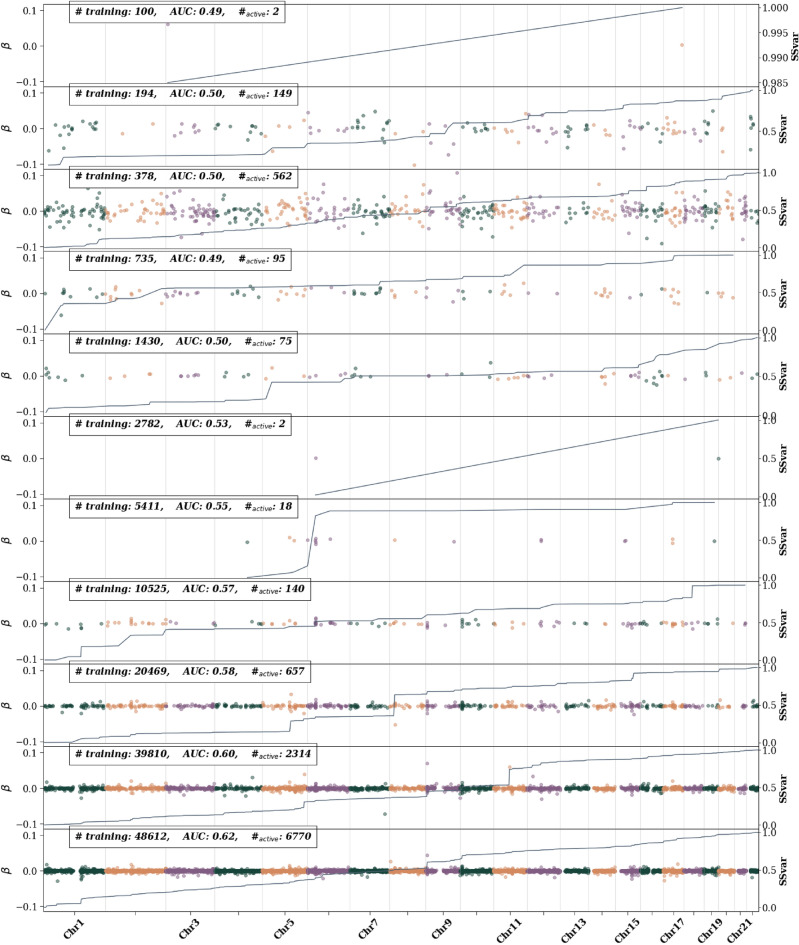
Asthma active SNPs—i.e., SNPs with non-zero $\beta$ weights—as training size is increased. The left axis shows the $\beta$ value and is represented by colored dots. Different colors are used to differentiate chromosomes. The right axis represents the single SNP variance (SSV) normalized to the total SSV. The solid line showes the cumulative SSV. The “training” label represents the number of cases used in training. The first 10 (from the top) training sizes use equal number of cases and controls. The final training size uses all possible remaining controls.

In Fig. 5 we see an example of the SNP content for an asthma predictor as it is trained with larger and larger training sizes. The LASSO weights $\beta$, single-SNP-variance (SSV), and training sizes are all further described in Section “Methods”. A notable feature from this figure is that, as training size is increased, the LASSO algorithm first adds more SNPs (becomes less sparse) while barely increasing the AUC. Eventually the algorithm gets rid of most of these SNPs, becomes much more sparse, and then quickly increase AUC as SNPs are then added again. This is an example of the algorithm “searching” for seemingly important features. Once some of the important features are identified, the algorithm finds more and more features that are important as evidenced by the rising AUC. Note that, as described in Section “Methods”, LASSO does not try to directly optimize AUC. This sparsity behavior (rising and falling before eventually finding important features that greatly increase AUC/correlation) appears in the rest of the predictors seen in the Supplementary Information and appears to be related to the Donoho-Tanner phase transition. This behavior can be seen in other features of each predictor—e.g., the SSV—as displayed in Supplementary Information. The Donoho-Tanner phase transition^68–71^ comes from the field of compressed sensing and generally refers to sharp behavior in the recovery of a sparse signal in under-determined systems. In simple language, signal recovery or predictor construction changes sharply as relevant parameters change. In genomics, key parameters are trait heritability, sparsity (i.e., number of important variants affecting the trait), and the sample size. In the case of genomics, crossing the phase transition boundary does not guarantee that true causal SNPs are found. For modern applications to this phase boundary for the LASSO see, e.g.,^72,73^ and for its application to genetics see^74^.

At the largest training sizes—i.e., after the phase transition—we also look at how the SNP content varies across cross-validation (CV) folds. As detailed in Section “Methods”, for case control phenotypes the largest training size includes as many controls as possible while all previous training sizes contain an equal number of cases and controls. In the Supplementary Information we have examples of the SNP content for the different types of large training sizes. For the largest training sizes we can also average over the CV folds to find the fraction of SSV per chromosome. Plots for SSV per chromome for each phenotype can be found in the Supplementary Information. There are several important takeaways from these results. The main metric for case-control phenotypes is much more affected by the number of cases than controls. Specifically, increasing the number of cases used in training increases the AUC, but increasing the number of controls in training, even by a factor of 2 or more, either does not change the AUC or leads to a very minor change. However, when looking at the single SNP variance, the number of both cases and controls do have appreciable effects. Take for example asthma. If we look for common features among the folds in maximal training (i.e., using all possible cases and controls) we see a modest jump in SSV at specific locations on chromosome 1, 2, 5, 6, 9, 10, 11, and 17. Additionally the increase in SSV on chromosome 17 is much larger on a single fold. In contrast, we also perform near-maximal training with the maximum number of cases and equal number of controls. Near-maximal training results in high impact regions (regions where the SSV increases by at least a few percent) at places similar to those found in maximal training (i.e., all cases and controls used), but the fraction of SSV at the beginning of chromosome 9 is much larger and the effect on chromosome 17 seems to be more smoothed out. This feature, which contrasts the two cases, is also seen when looking at the SSV fraction per chromosome. These plots, and the corresponding plots for the other phenotypes, are found in the Supplementary Information.

To interpret these plots we have collected and identified every SNP that accounts for at least $1\%$ of total SSV. These tables are found in the Supplementary Information. Here we review the most important SNPs and compare them to known results, i.e. we identify genes, associated with the particular phenotypes, that are within at least 2 million base pairs of a SNP accounting for $>1\%$ SSV. We choose a 2 million base pair distance to be a conservative measure of possible long-range LD^75^. All predictors, except those for Lipoprotein A, include SNPs that account for at least $1\%$ of total SSV but are not near any known gene that associates with the phenotype. Additionally, we list the p-value associated with a GWAS on the raw (case control) or adjusted (continuous) phenotype to highlight that many of these important LASSO SNPs would be missed by a traditional GWAS approach.

SNPs which we identify that are located near known associated genes for several phenotypes are listed here:

- Asthma: SNPs around FLG, IL1RL1, TSLP, IL33, SMAD3, HLA-DQ, RORA, CLEC16A, and SERPIN7 which have been previously been identified by GWAS^76–80^
- Atrial fibrillation: at least 164 SNPs have been identified in GWAS studies^81–84^, and while none of these exact SNPs appear in the 58 SNPs identified using LASSO, many of the LASSO SNPs are located around the genes KCNN3, PMVK, LMNA, KIFAP3, PRRX1, SCN5A, SCN10A, PITX2, FAM13B, WNT8A, CAV1, SH3PXD2A, HCN4, ZFHX3, RPL, and FBXO32 which are associated with some of the gwas SNPs
- Breast cancer: none of the SNPs identified here are near the genes identified in^85^
- Type 2 Diabetes: we find relevant SNPs in the associated genes GCKR, TCF7L2, and SLC12A1^86^
- Type 1 Diabetes: we find SNPs near HLA-A, TRIM26, MICA, HLA-DRB1, and LAT^87^
- Coronary Artery Disease: we find SNPs near the associated genes PCSK9, PLPP3, IL6R, MIA3, VAMP5, ZEB2, SLC22A4/A5, SLC22A3, LPAL2, LPA, PLG, and CETP^88,89^
- Hypertension: we find important contributions near the ULK4, NR3C2, PRRC2A, and NOS3 genes^90^
- Direct Bilirubin: we find results near the UGT1A1, SLCO1B3, and SLCO1B1 associated genes^91,92^
- Body Mass Index: we find SNPs in genes TMEM18 and the well studied FTO – both appear with more than $1\%$ SSV for BMI^93^
- Height: Liu et al.^94^ categorized over 400 genes associated with height that were later reanalyzed by Yengo et al.^95^. Of these, SNPs near ORC1, COL11A2, FANCE, BRAF, ACAN, ANKRD11, CDK10, CDT1, FANCA, GALNS, and RPL13 all appear in our analysis
- Lipoprotein A: all SNPs appear in or near the genes LPA, LPAL2, and SLC22A3 which are all known to be associated with Lipoprotein A levels^96,97^ and additionally contribute to coronary artery diseases^98^.

There are several interesting aspects of examining SNPs in this manner. First we note that we are interested in *common* variants and exclude SNPs with a minor allele frequency below 0.001 to avoid any spurious associations. Because of this, rare variants can’t appear in our analysis, even if they are known to be associated with a phenotype. An example of this can be seen in the case of breast cancer where the BRCA mutations aren’t included in our analysis. Interestingly there are SNPs that have previously been identified via GWAS, that are available on our array, but are *not* selected by LASSO. An example would be rs116716490 which is part of the ZBTB10 gene, previously associated with asthma via GWAS^78^, but not selected by LASSO.

A more coarse grained interpretation of the impactful regions can be found in the Supplementary Information. There, we examine—for each trait—the fraction of SSV that resides on each chromosome. For case-control phenotypes, we also compare the result for max possible training (i.e., the max possible cases *and* controls), and training with the largest possible equal number of cases and controls. We highlight some of the notable results. For atrial fibrillation, max training and equal case control training both find a large fraction of SSV on chromosome 4, but the signal is much larger for equal cases and controls. On chromosome 15 there is a large signal for max training, but not for equal cases and controls. For type 2 diabetes a large fraction of SSV is on chromosome 10, but the signal is largest for equal cases and controls. For type 1 diabetes. equal cases and controls find a strong signal on chromosome 6 while max training also finds signals on chromosomes 1, 11, 15, and 17. For CAD, the equal case control training finds the largest signal in chromosome 6 while max training finds similarly large signals in chromosomes 1, 2, 3, 6, and 12. Hypertension shows varying signals all throughout the chromosome with the most precise signal coming from chromosome 1. Breast cancer has the most diverse differences between the two types of training with large signals for equal case control training on chromosomes 10 and 16, and large signals on 7, 11, 16, and 19 for max training. For continuous phenotypes we only have one measure of SSV per chromosome. For direct bilirubin the largest contributions are on chromosomes 1 and 2. For BMI the largest signals are on chromosomes 1, 2, and 3. For height there is strong signal throughout most of the genome. Finally for Lipoportein A, the signal seems to be concentrated on chromosome 6.

### Sparsity and heritability

**Figure 6:**
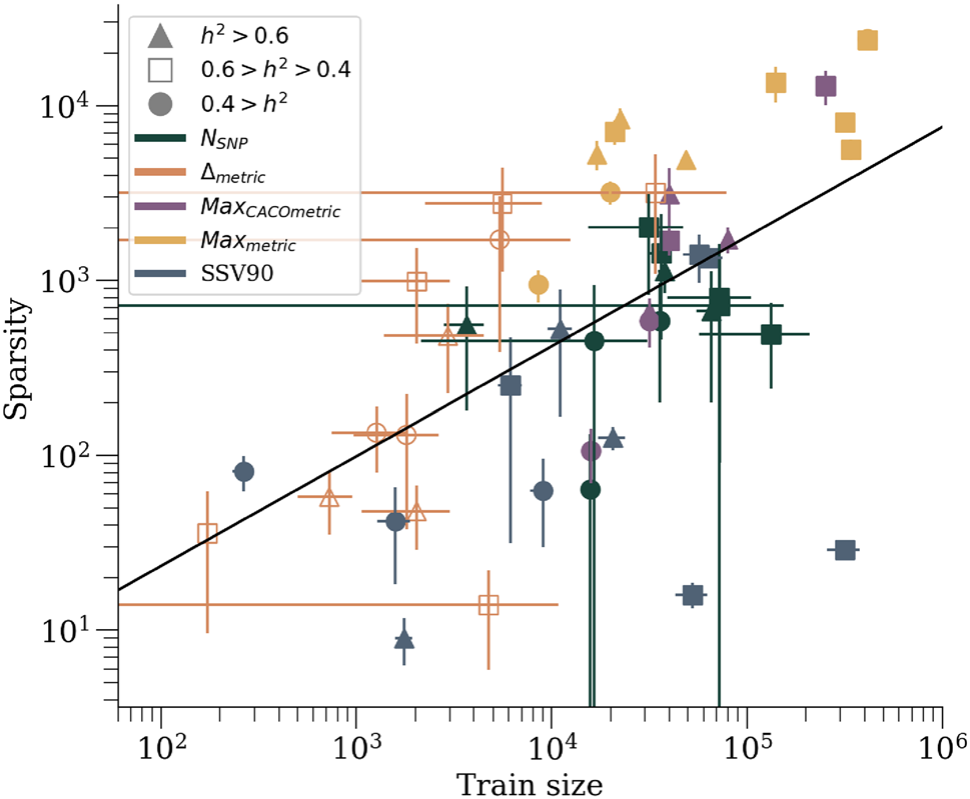
Sparsity measurements, as a function of training size, for all 11 traits. Different markers correspond to different (arbitrary) estimated heritability groupings. Different colors correspond to different versions of sparsity. Heritablity here for case-control phenotypes is *broad sense* heritability reported from twin/family study literature, whereas GCTA was used to estimate heritability for continuous phenotypes. Low heritability traits (circles) include: atrial fibrillation, breast cancer, and BMI^100,101^. Medium heritability traits (squares) include: CAD, hypertension, direct bilirubin, height, and lipoprotein A^102,103^. High heritability traits (triangles) include: asthma and type 1/2 diabetes^104–106^.

We can see examples of all the definitions of sparsity in Fig. 6 where the definitions themselves are explaind in Section “Sparsity”. The traits are roughly grouped according to their heritability estimate using GCTA^99^. Using all metrics together, the scatter of data can be regressed linearly in $log_{10}$ scales in both training size N and sparsity s. We find $log_{10}(s) = 0.7_{(0.1)} log_{10}(N) + 0.1_{(0.5)}$ (the black line in Fig. 6) with an $r^2=0.43$. This weakly implies $s\sim N^{0.7}$ (or conversely $N \sim s^{1.4}$). In^74^ it was shown, using simulated data, that for $h^2=0.5$ (where $h^2$ represents the narrow-sense heritability) the compressed sensing phase transition occurs at $N\approx 30\text { s}$. The result reported here is consistent with this previous prediction, but tighter error bars are required to completely determine the coefficient and its dependence on $h^2$.

**Figure 7:**
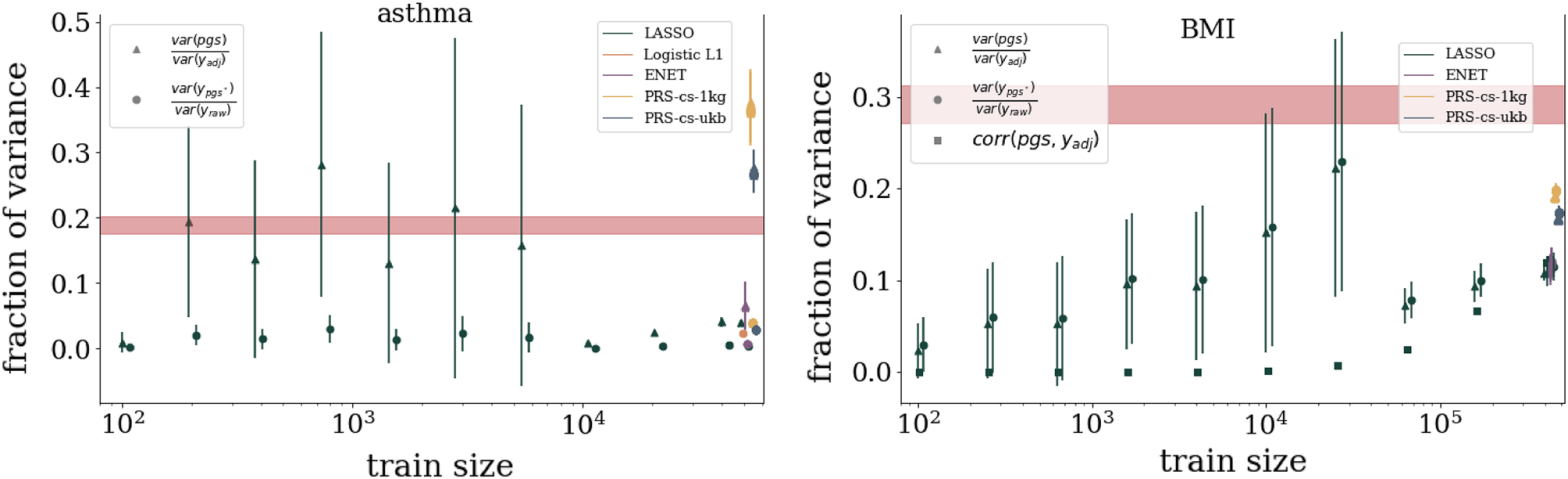
Estimates of the fraction of variance explained from *purely genetic* contributions for asthma and BMI. There are various ways to estimate the variance explained as explained in Section “Methods”. Similar plots for the other traits are found in the Supplementary Information.

Finally we can see estimates of heritability using a variety of metrics in Fig. 7 and in the Supplementary Information. In Fig. 7 we see examples of these heritability estimates, from a LASSO training, as a function of training size. (Additional phenotypes are in the Supplementary Information). Additionally, for the largest training size, we also record these heritability estimates for the other training methods. As a function of training size we generally see the phenotype dependent phase transition behavior noticed before. After a critical amount of training data is used, the error bars for all methods greatly decrease. Identifying this transition behavior is important; it indicates that central values *below* the critical training size may suggest a deceptively high estimate. For most phenotypes–regardless of training size, metric, or method–the estimated heritability is below what can be estimated from GCTA. However, there are a few exceptions: for asthma and type 1 diabetes, at the largest training size PRScs using the $\blacktriangle$ metric outperforms GCTA; for height the $\bullet$ metric generally, and PRScs at largest training, outperforms GCTA; and for Lipoprotein A, every metric and method *except* PRScs results in a higher value than GCTA.

## Methods

### Predictor training

We start with a very brief description of the general training pipeline for generating predictors: **step (1)** initial populations are separated into groups; **step (2)** quality control (QC) for both phenotypes and genotypes; **step (3)** phenotypes are regressed on covariates and adjusted phenotypes are built; **step (4)** SNP set is filtered down to a computationally manageable size; **step (5)** machine learning on adjusted phenotype and genotypes using cross-validation; **step (6)** predictors are tested on withheld groups.

As an initial grouping of UKB data, we separate participants using self-reported ancestry (a principal component adjustment is done below). We use participants reporting ancestry as White (EUR), South Asian (SAS), East Asian (EAS), and Black (AFR). Additionally we construct an American ancestry (AMR) set done using the approach from^7^ and described in the Supplementary Information (there is a small 60 person subset of the 322 AMR labeled participants who also self identify as white and could appear in the training. This set is so small that its effects are assumed to be negligible). From the EUR population we identify genetic siblings as described in the Supplementary Information and remove them to use as a final testing set. The remaining EUR population is used for training.

Step 2 involves performing quality control on both the genotypes and phenotypes. Full phenotype definitions, including UKB codes, are given in the Supplementary Information. For phenotype quality control we exclude any missing values or negative values (usually used as a placeholder or indicator in the UKB). For continuous phenotypes we average over all recorded measurements (many participants are measured on repeated visits, but there is not a consistent number of visits per participant). Case control conditions are defined with a logic ‘or’, i.e., if the participant is recorded as a case for *any* relevant code, then they are counted as a case. The UKB array contains 805,426 SNPs. We run QC using PLINK to filter out (remove) variants (SNPs) with more than 3% missing values, samples (participants) with more than 3% missing values, and variants with minor allele frequency less than 0.001 (i.e. 0.1%). After QC this leaves 663,533 SNPs and 487,048 participants. (Exact number of participants can slightly vary due to participant withdrawal from the UKB program.) Finally we reduce our SNP set down to only the autosome as this allows us to roughly double our training base, i.e. use all sexes in training (except for sex specific phenotypes).

Step 3 can be briefly described as sex-specific z-scoring and covariate adjustment. The z-scoring is only done on continuous phenotypes while covariate adjustment is done for all phenotypes. We z-score to improve the efficiency of the machine learning algorithms (e.g., using normalized data lowers the risk of large numbers appearing in a gradient descent algorithm). If we assume that we are looking for common genetic factors that are independent of sex then we can z-score *each sex individually* and roughly double our training data. The ultimate aim is to identify genetic variants that we are most confident are related to a phenotype. To do this we assume that common covariates and population stratification *have a maximal effect*. That is, we regress covariates on the raw (or z-scored) phenotype and then adjust (i.e., create a residual phenotype) phenotypes for the contribution explained by these covariates. Common covariates included are: age, sex (except for sex specific traits like breast cancer), and the top 20 principal components as computed by the UKB. Even though we do a sex specific z-scoring, at this stage we still assume sex can have an impact. That is, for phenotype, $\vec {y}$, and covariates, $\bar{H}$, we regress: $\vec {y}\sim \vec {\alpha } \bar{H}$. Then we can construct an *adjusted phenotype*, $\vec {y}_{adj}$, that just includes the residual signal: $\vec {y}_{adj} = y - \vec {\alpha } \bar{H}$.

Unfortunately, before we employ a machine learning algorithm, we have to reduce down the $\sim 600k$ SNPs to a computationally manageable number. The exact computational details will depend on the machine being used to run the analysis and whether or not computational cost saving measures can be used (e.g., parallelization). For our analysis, as shown in the Supplementary Information, the time to run lasso scales as a function of training data, *N*, roughly as $\sim N^{1.35}$. Additionally, the memory and CPU usage, while growing more slowly than exponential, grows quickly. For step 4, we choose to subset our SNP set by selecting the top 50k SNPs (10k for penalized logistic regression which is more computationally intensive) via GWAS with the training set.

Next, in step 5 we run machine learning algorithms. For LASSO and Elastic Net we use the Scikit-learn^107^ lasso path linear_model.lasso_path and enet path linear_model.enet_path algorithm. The Elastic Net algorithm minimizes the objective function,

1 $$\begin{aligned} \frac{1}{2 N} ||\vec {y}_{adj} - \bar{X}\cdot \vec {\beta }||^2_{L_2} + \lambda \left( L_{1 ratio} |\vec {\beta }|_{L_1} + \frac{1 - L_{1 ratio}}{2} ||\vec {\beta }||^2_{L_2}\right) , \end{aligned}$$

where $\bar{X}$ is the normalized genotype matrix, $\vec {\beta }$ the regression weights, $\lambda$ the hyperparameter, $L_{1 ratio}$ relatively weights the $L_1$ and $L_2$ penalties. As $L_{1 ratio}\rightarrow 1$ this becomes LASSO and $L_{1 ratio}\rightarrow 0$ it becomes ridge regression. For all phenotypes we run for 5 different ratio weights: $L_{1 ratio} \in \{.1,.3,.5,.7,.9\}$. For $L_1$ penalized logistic regression we use the Scikit-learn function linear_model.LogisticRegression with an L1 penalization and parallelize it using the Multiprocessing package Pool function. For all methods of (penalized) regression we use five-fold cross validation and use a 2,500 sample validation set (withheld from training) for hyperparemeter selection. For case-control phenotypes, the validation set is equal number of cases and controls. Finally, we also use PRS-cs^108^ which is a Bayesian shrinkage prior which runs directly on summary statistics (i.e., GWAS output) and an LD matrix. We use three fold cross validation and run with the global shrinkage/sparseness parameter, $\phi \in \{10^{-1},10^{-3},10^{-5},10^{-7},10^{-9}\}$ and the local scale parameters $\{a,b\} \in \{1/2,1,3/2\}$.

In the final step 6 we apply the predictors to all testing sets: the EUR genetic siblings set, an SAS set, an EAS set, an AFR set, and an AMR set. Case control phenotypes are evaluated by computing the Area Under the receiver operator Curve (AUC) which compares the true positive rate to the false positive rate, i.e. how well the predictor is calling cases and controls. Within this work we generally report the AUC *only using the genetic weights* to emphasize how well cases and controls can be identified using only genetic information. For case control phenotypes we can also compute an odds ratio, as a function of polygenic score, as seen in Fig. 3. This is an *inclusive* odds ratio: at each score, we compute the ratio of the number of cases to controls (normalized to the total cases and controls) of all individuals with that score or a higher score. I.e., the 80th percentile represents all individuals with a score $\ge 80\%$. For continuous phenotypes we simply report the correlation between the polygenic score and the adjusted phenotype, $\text {Corr}(PGS,\vec {y}_{adj})$, and again this estimates the *purely genetic* contribution.

In addition to standard metrics like AUC and correlation, we are often interested in the amount of variance described by a particular polygenic predictor. For a particular polygenic score, the variance is described by

2 $$\begin{aligned} \text {var}(PGS)&= \text {var}\left( \sum _i x_i\beta _i\right) = \sum _i\left( \text {var}(x_i\beta _i)+2\sum _{j<i}\text {cov}(x_i\beta _i,x_j\beta _j)\right) \end{aligned}$$

3 $$\begin{aligned}&= \sum _i\left( 2\beta _i^2(1-f_i)f_i+2\sum _{j<i}\text {cov}(x_i\beta _i,x_j\beta _j)\right) \approx \sum _i 2\beta _i^2(1-f_i)f_i \equiv \text {SSV}\,, \end{aligned}$$

where the approximation defines the Single SNP Variance (SSV). This approximation assumes that the covariance between SNPs is small. For most sparse methods, this covariance is minimized to enforce sparsity. For example, in the objective function Eq. (1), adding extra SNPs with non-zero weights amounts to decreasing the first term and increasing the second term. For an added SNP that is highly correlated with another SNP with non-zero weight, the decrease in the first term is likely smaller than the increase in the second. The accuracy of this approximation is demonstrated in the Supplementary Information.

#### Sibling tests

For both types of phenotypes there are sibling specific tests that we perform. These were developed in^64,65^. All siblings used in these analyses are *genetic* siblings identified using *KING*^109,110^ and filtered in a manner similar to^110^. More detail is provided in the Supplementary Information. Siblings generally share a similar environment growing up which helps to control for some external factors. For case control phenotypes we can consider Affected Sibling Pairs (ASPs), which are pairs of siblings where one person is a case and the other a control. We can then look at the correct selection rate, that is the fraction of the time the person with the larger polygenic score corresponds to the case. This computation can be done again while requiring that the sibling pair’s scores are at least 1.5, 2, or 2.5 standard deviations different. For continuous phenotypes we show a similar selection rate for the amount of time the person with the larger polygenic score has the larger phenotype. Again this rate can be recomputed with the requirement that the phenotype difference between the siblings is at least 0.5, 1, or 1.5 standard deviations.

### Metric projection

We can model improvement in predictor performance metrics as a function of training data size. In Fig. 4 we see examples of this for asthma and BMI. We use four functions, which have left and right asymptotes, to model this growth: sigmoid, inverse tangent, error function, and hyperbolic tangent. The hyperbolic tangent can be written as a rescaled version of a sigmoid function. We use that as a cross-check, i.e. we make sure both functions give the same results, to avoid fitting routines getting stuck in a local minimum. For all functions, four parameters are used to fit the performance, e.g., $\text {erf4}(x;a,b,c,d) = a +b\,\text {erf}(c(x+d))$. These parameters are subject to physical constraints. Roughly this corresponds to $0.5<\text {AUC}<1$, $0<\text {Corr}<1$, metric growth is a function of training size, and the growth happens when training with multiple samples. Care is taken to incorporate uncertainty from cross-validation and finite data sizes. More details about the functions, uncertainty calculations, and fitting results are given in the Supplementary Information. The non-linear fit of each functional form involves computing a Hessian matrix as a function of the parameters $\{a,b,c,d\}$. The inverse of this matrix is the correlation matrix for the model’s fit parameters. Using these empirical correlations we build a MC method that relies on a Cholesky decomposition. For a given correlation matrix ${\underline{C}}$ of fit parameters, we can generate a correlated set of MC parameters, $\vec {Y}$, from random numbers, $\vec {X}$, via a Cholesky matrix, ${\underline{L}}$:

4 $$\begin{aligned} {\underline{C}} = {\underline{L}}\,{\underline{L}}^* \qquad \rightarrow \qquad {\underline{L}}\vec {X} = \vec {Y} \, . \end{aligned}$$

    We can then build MC bands as seen in Fig. 4. For both types of phenotypes, these projection bands are informative: they indicate **(1)** the asymptotic (i.e. best possible) metric obtained from infinite data, as in Table 2**(2)** which traits are nearing that asymptotic maximum or which are still improving substantially with more training data, and **(3)** what sample size other biobanks will need to obtain meaningful results. These projection bands can be compared to the application of European trained predictors applied to other ancestry groups. In Fig. 1 we see that predictors built using data from TPMI and AoU will greatly surpass the results of UKB trained predictors when applied to distant ancestry groups.

More details can be found in the Supplementary Information and in the code examples found in the affiliated Github repository.

### Sparsity

The sparsity associated with a phenotype can be defined in *several* ways and it is not *a priori* clear which definition is most appropriate. The definitions used here are:

1. The number of SNPs with non-zero weights using all possible cases and controls. This quantity can appear to continue to grow without reaching an asymptote even though traditional metrics (e.g. AUC or correlation) appear to asymptote. However, this definition is obviously impacted by training sample size and difficult to compare between datasets/biobanks. Assuming infinite data generated by a linear model plus noise, compressed sensing guarantees complete signal recovery and the sparsity will reach an asymptotic value corresponding to the underlying model that generated the data^74^. Additionally, for algorithms like LASSO there is debate as whether to use the maximal metric for hyperparameter selection or to step one standard deviation “back” (i.e. towards fewer features) in hyperparameter space to avoid over-fitting when applying to outside true testing sets. (see^11^ and references therein).
2. We can use the previous definition but for the training case with the maximum number of equal cases and controls. The same caveats from definition 1. apply.
3. Because we expect traits not to be 100% heritable, we expect metrics to asymptotically approach a limit value. We can look at relative increase in these metrics to try to identify the point of largest growth (or above some cut), i.e. the inflection point. We can do this by looking at a *relative metric*, i.e. $(y_{i+1}-y_i)/y_i$. We can look at the sparsity of the predictors at this point as a second version of sparsity.
4. Instead of looking at the metrics, we can instead look at the relative increase in the number of features (SNPs) at each training size and look for the relative maximum increase (or increase above some cut) in features to define a sparsity value.

Comparisons for these definitions can be seen in Fig. 6.

### Fraction of variance explained

Heritability, $H^2$, is traditionally defined as the proportion of phenotypic variance explained by genetic factors. All the work discussed here involves linear genomic prediction, i.e. that $PGS \equiv y_{PGS} = \sum \vec {\beta } \cdot \bar{X}$. The proportion of variance explained by a linear model is generally referred to as the narrow-sense or linear heritability, $h^2_{linear}$. There are several ways we can estimate the fraction of variance explained via the results of a linear predictor. First, we note that our linear predictors are trained after sex specific z-scoring. Because of this, the overall scale of $y_{PGS}$ (i.e. the variance) is expected to be of the order of the adjusted phenotype, *not* the original raw phenotype. Therefore, we can consider the ratio of the variance of the PGS to the adjusted phenotype ($\blacktriangle$ denotes $var(pgs)/var(y_{adj})$) or we can undo the sex specific z-scoring only on the PGS and compute the ratio of variance in this rescaled PGS to the raw phenotype ($\bullet$ denotes $var(y^*_{pgs})/var(y_{raw})$). Finally, for continuous traits we can simply look at the correlation between pgs and adjusted phenotype as in^11,51,64^.

In both Section “Biobank projections” and in Section “Sparse output interpretation” we refer to the heritability estimated via REML and LDSR. The LDSR results are reported from^111,112^. The REML results are produced using the GCTA software^99,113^. All GCTA computations used 350,000 SNPs and the following number of samples: asthma 20,469; atrial fibrillation 9027; type 2 diabetes 11,077; type 1 diabetes 1755; CAD 11,077; hypertension 11,659; breast 9027; direct bilirubin 21,544; BMI 25,118; height 21,544; lipoprotein A 21,544.

### Public Data

Raw data is not available for direct sharing, but can be obtained via application to the UK biobank (https://www.ukbiobank.ac.uk/enable-your-research/apply-for-access). Predictors (i.e. SNPs and weights) and some code used to produce results can be found at https://github.com/MSU-Hsu-Lab/biobank-scale-methods-paper-2023/.

## Discussion

Development of a large genetic database or biobank is a significant endeavor. To ensure that these efforts are focused on phenotypes and recruitment sample targets that will produce the most beneficial results, we have developed a novel method for projecting polygenic score performance to the size of novel biobanks. We make specific predictions for TPMI and AoU based on their currently stated recruitment goals and known population prevalences for various phenotypes. These and other biobanks are still recruiting and genotyping participants; hopefully these results can be used to plan recruitment targets and drive new analyses.

Sparse methods optimize prediction while activating as few features (SNPs) as possible during training. In contrast, non-sparse methods construct predictors in which potentially every SNP in the genome has non-zero (but possibly very small) effect size. Because sparse predictors perform about as well as predictors constructed using non-sparse methods^114–116^, it is reasonable to conclude that actual genetic architectures are themselves sparse. For all known complex traits and disease risks, only a small fraction of common SNPs are required to build a predictor which performs nearly, or equally, as well as any non-sparse predictor.

In this paper we analyzed many aspects of sparse predictors. Within the class of algorithms that produce spare predictors we find that LASSO, also known as compressed sensing, is competitive with other more complex techniques. In fact, methodological improvements in predictor training, although important, generally produce improvements which are relatively small (in rough terms, of order 5 or 10%), whereas increases in data size are likely to produce much larger gains. Additionally, we demonstrate the performance characteristics promised from the compressed sensing literature. Specifically, we demonstrate phase transition behavior in performance using actual genotype/phenotype pairs—above a certain data threshold we recover the SNPs which provide the strongest contribution to performance metrics. Additionally, techniques to improve predictor portability (e.g.,^52,54,54–61^) rely on understanding the SNP content of the original predictor. This issue is considerably simplified if the predictors are trained using sparse algorithms to narrow down the possible resulting SNP set.

We develop a methodology for projecting the performance of LASSO on larger datasets. This method can be applied to anticipated future biobanks and to analysis which is forthcoming on existing biobanks. Specifically, we project that a predictor trained in the Taiwan Precision Medicine Initiative for asthma can achieve an AUC of $0.63_{(0.02)}$ and for height a correlation of $0.648_{(0.009)}$ for a Taiwanese population. For comparison, the measured values are $0.61_{(0.01)}$ and $0.631_{(0.008)}$, respectively, for UK Biobank trained predictors applied to a European population. We also show that, in terms of AUC, atrial fibrillation, type 2 diabetes, CAD, and breast cancer will more than double their signal if trained on larger datasets.

Other researchers have investigated the question of how prediction and association results depend on sample size and have tried to predict the results for future large sample sizes. We highlight here some lines of research in this direction so that we can emphasize how our new analysis is different. First, most of the previous work involves GWAS based methods (e.g.,^95,117–123^) whereas this work involves machine learning algorithms that go beyond single marker regression. Secondly, much of the previous work makes assumptions about how performance metrics, e.g. AUC or Area Under receiver operator Curve, depend on trait heritability, model parameter effect distributions, and their functional form. For example, in^124,125^, AUC is modeled with a cumulative distribution function for the Gaussian distribution and depends on the population variance of the PGS. Using these approaches, after fitting to the training of a single PGS, predictions can be made for other training sample sizes. In this work we make no such assumptions about the functional form of AUC or the scaling dependence of an underlying genetics effects model. Instead we simply assume that AUC is a finite and bounded quantity. We then use a series of different functional forms to model the growth of AUC. These functional forms are chosen because based on parsimony, i.e. that they have the fewest number of parameters for functions that satisfy the simple physical constraint. This is an advantageous approach when there are limited or small samples with which to train. In contrast, in this work for each phenotype we use 11 different training sizes to model the growth of performance metrics. In using such a large number of different training sizes, we developed a novel method for taking into account the uncertainty/error associated with the metrics when modeling the growth as a function of training size.

Throughout this work we are primarily concerned with sparse methods that are trained directly on genomes. The one exception being the PRScs method, which is a summary-statistics (GWAS) based method. We stress that that our main results are concerned with training methods that directly use genome matrices themselves and might not generalize to the comparison to generic GWAS based methods. For example, although a simple LASSO regularly outperformed or performed comparably to PRScs, it is not necessarily true that this will be the case for all GWAS based methods with similar training sizes—especially if it includes additional information like fine-mapping, functional information, other -omics, etc. However, it has been found that for some phenotypes, e.g. height, methods like LASSO usually require much smaller sample sizes than GWAS based methods to find the same phenotype-PGS correlation^51,95^. GWAS based methods however have an advantage over direct genome training: it is relatively easy to combine GWAS summary statistics from disjoint studies to increase sample size. This is advantageous as it requires less individual computing resources for training while still increasing sample size. For example, meta-analysis of GWAS for height and BMI has found novel loci^122^. Some GWAS based methods, like PRScs, require information about the LD structure. Computing LD information can be computationally intensive, but it only has to be done *once* and then the results can be efficiently reused^126,127^.

One of the main challenges of polygenic prediction is that most of the available data comes from studies in which most of the participants are individuals of European ancestry. Thus the current predictors perform poorly when applied to other ancestry groups (e.g., East Asians or Africans). The hope of developing methods which “transport” a predictor trained on one ancestry to other ancestral groups remains, but despite ongoing research efforts the goal remains elusive. In the absence of such a breakthrough, much larger cohorts of non-Europeans are required to produce predictors of comparable quality. The new methods developed here allow us to predict, e.g., how well new predictors trained on biobank-scale data from the Taiwan Precision Medicine Initiative (which is planned to surpass 1 million genotypes) and the US All of Us project. In the former case, we predict that new predictors will exceed AUC / correlation metrics for current best-in-class predictors for European ancestry.

## Supplementary Information

Supplementary Information.

## Data Availability

Access to the UK Biobank resource is available via application (http://www.ukbiobank.ac.uk). UKB data was collected under policies conforming with national and local requirements and subject to privacy rights. (https://www.ukbiobank.ac.uk/privacy-policy) All data handled by researchers in this work was de-identified. MSU researchers working on this project do not have access to any identifiable data and are covered under MSU IRB [STUDY00006493].
