# Supplementary material for: Biobank-scale methods and projections for sparse polygenic prediction from machine learning
Source: Sci Rep. 2023 Jul 19;13:11662. doi: 10.1038/s41598-023-37580-5 (PMC10356957; doi:10.1038/s41598-023-37580-5)
Supplement: Supplementary file 1 — Supplementary Information. [file 41598_2023_37580_MOESM1_ESM.pdf]

Biobank-scale methods and projections for sparse polygenic prediction from machine learning

Timothy G. Raben, Louis Lello, Erik Widen, Stephen D.H. Hsu

July 12, 2023

1 Data

1.1 Populations

The UK Biobank provides a self-reported ethnic background field (field 21000) on which to filter into various super-populations – European, American, South Asian, East Asian and African. Self-reported Europeans are identified by the codes 1001, 1002, 1003 or 1 (White, British, Irish, Any other white background). Self-reported South Asians are identified by the codes 3, 3001, 3002, 3003, 3004 (Asian or Asian British, Indian, Pakistani, Bangladeshi, Any other Asian background). Self-reported East Asians are identified by the code 5 (Chinese). Self-reported Africans are identified by the codes 4, 4001, 4002, 4003, 4004 (Black or Black British, Caribbean, African, Any other Black background). Additionally code 6 is "Other ethnic group" and codes 2001-2004 are mixed background.

In addition to self-report, genetic ancestry is computed with ADMIXTURE Version 1.3.0 [1] and the 1000 genomes phase 3 as the reference panel. First, 1000 genomes was filtered down to SNPs which overlap the UK Biobank and then further sampled to 23,326 SNPs for computational ease. With this SNP subset, Admixture was run unsupervised on the 1000 genomes with 5 populations. The output P files (the allele frequencies of the inferred ancestral populations) were then applied via projection mode to the UK Biobank in batches - resulting in each individual with a percentage in each of the 5 super-populations (given by the ancestry fractions Q file).

The 5 different components were verified to correspond to the different super-populations in 1000 genomes. A set of American ancestry individuals was selected by keeping all individuals who had at least 35% on the American component of the analysis. This results in 322 individuals who correspond to the following report codes: 6:206, 1003:56, 2004:33, 1001:3, 4001/2003/2002/2001/2/1:1. These 322 individuals are considered the American super-population test group and are withheld from the other self-reported test sets.

1.2 Siblings

The UK Biobank estimated kinship coefficients for individuals using *KING* [2] as described in [3]. The UKB records related pairs of degree 3 or closer and provide the results as a single pairwise kinship table which is provided with the UK Biobank data. The set of siblings used in this work is identified by filtering this pairwise list on kinship coefficient and IBS0 in a manner similar done by the UKB (see the supplement of [3]). Specifically, to be included as a sibling pair, we keep all pairs with kinship coefficient larger than 0.176 and IBS0 larger than 0.0012. This procedure results in 22,667 sibling pairs, in agreement with [3].

1.3 Phenotypes

The phenotype definitions used here involve various UKB-fields to define case and control status. The definitions are inclusive in that if any of the indicated data fields report a diagnoses or self report then the individual is counted as a case. For continuous phenotypes, the average of all measurements was used. The definitions used the ICD9, ICD10 and OPCS4 codes in UKB-fields 41271, 41270, 41272; self-reported non-cancer codes from UKB-field 20002 and cancer codes in UKB-field 20001. Additionally, some diseases were specifically included in the intake questionnaire or otherwise used other UKB-fields, which also are listed below.

There might be some quantitative performance gains that could come from the inclusion of more fields/codes for the phenotypes. Performance gains might also come from an analysis of related information like medication, lifestyle choices, and family history.

Training and evaluation of the predictors used a UKB download date of April 2021. The following disease definitions were used:

Asthma non-cancer codes: `1111`; ICD9: `49300, 49309 ,49310 ,49319 ,49390 ,49399`; ICD10: `J450,J451,J458`

Atrial fibrillation non-cancer codes: `1471, 1483`; ICD9: `4273`; ICD10: `I480-I484, I489`

Breast cancer cancer codes: `1002`; ICD9: `174, 1749`; ICD10: `C50, C500-C506, C508, C509`; field ID 40001: `C50, C500-C506, C508, C509`; field ID40002: `C50, C500-C506, C508, C509`; field ID 40006: `C50, C500-C506, C508, C509`; field ID 40013: `174, 1749`

Coronary artery disease non-cancer codes: `1075`; ICD9: `410, 4109, 412, 4129`; ICD10: `I21, I210-I214, I219, I21X, I22, I220, I221, I228, I229, I23, I230-I236, I238, I241, I252`; OPCS4: `K401-K404, K411-K414, K451-K455, K491, K492, K498, K499, K502, K751-K754, K758, K759`

Diabetes type I non-cancer codes: `1222`; ICD10: `E100-E109, O240`

Diabetes type II non-cancer codes: `1223`; ICD9: `25000, 25002, 25010, 25012, 25020, 25022, 25030, 25032, 25040, 25042, 25050, 25052, 25060, 25062, 25070, 25072, 25080, 25082, 25090, 25092`; ICD10: `E11,E110-E119`

Hypertension non-cancer codes: `1065,1072,1073`; ICD9: `4010,4011,4019,4050,4051,4059,4160,6420,6423,6429`; ICD10: `I10`

Body Mass Index field ID: `21001`

Direct Bilirubin field ID: `30660`

Height field ID: `50`

Lipoprotein A field ID: `30790`

1.4 Disease Prevalence

For asthma, recent surveys have found a prevalence of 10.9% in African Americans [4] (consistent with statistics from a decade earlier [5]). Recent studies have found the prevalence of asthma greatly increasing in Taiwan over the past several decades [6] (with rates in children reaching as high as $\sim 20\%$), however we use the more conservatively reported number of 7.9% reported in [7]. The prevalence of asthma in Hispanic communities varies enormously up to $\sim 30\%$ [8], but we use the conservative figure of 4.9% for Mexicans given in [8].

For atrial fibrillation a prevalence of 0.35% has been found for African Americans [9]. In Taiwan, a sex specific prevalence of 14% for men and 7% for women was found [10]. Because TPMI recruitment is still ongoing, this

was averaged to 10.5%. For Hispanics and Latinos in the USA, after doing a weighted average over prevalences in sub-ancestry groups, an overall prevalence of just 1% was found[11].

Breast cancer prevalence in Hispanics fluctuates slightly within ancestry subgroups in the USA, but was recently observed to be 1% [12]. For African Americans the prevalence is much higher at 11.5% [13]. In Taiwan the prevalence is only .83% [14].

CAD as defined in 1.3 is a complex phenotype made up of various self report, ICD, and OPCS codes. While this definition is widely used, e.g. in [15], it is not necessarily consistent with other definitions of CAD reported in survey literature. Because of this we use the reported prevalence in UKB, 5%, as the same prevalence in other biobanks. This is consistent with reports of CAD prevalence in Taiwan (4% sex averaged) [16], African Americans (5.4%) [17], and Hispanics in the USA (5.1%) [17].

It has been shown that there are differences in the rate and outcomes of hypertension in African Americans and caucasians in the USA [18]. Here it was found that the sex averaged rate of hypertension in African Americans is $\sim$ 42%. In Taiwan, the prevalence of hypertension is 26.1% according to a 2017 national survey [19]. Among Hispanics in the USA, the prevalence varies based on ancestry, but affects roughly 30% of the population [20].

The prevalence of type 1 diabetes has been found to be 0.57% among African Americans [21], 0.18% in American Hispanics [22], and as little as 0.01-0.05% in Taiwan [23, 24]. In contrast, type 2 diabetes is much more prevalent, being counted in roughly 13% of African Americans [25], 9.5% of American Hispanics [25], and 8.3% of Taiwanese [26].

For continuous traits—BMI, Direct Bilirubin, height, and Lipoprotein A—we assumed the same reporting rate as the UKB. For all 4 of these traits, measurements were recorded for 97% of UKB participants. This same rate was assumed for the other biobanks.

2 Uncertainty Analysis

Performance metrics—AUC, correlation, etc.—are often reported with a single measure of uncertainty, e.g. a standard error. In this work, the uncertainty of the metric estimate is a *key* ingredient to model predictive behavior. To this end, in this section we detail exactly how we characterize uncertainty.

2.1 Standard Errors

Standard errors (SE) characterize the precision with which a statistic has been measured. SEs generally tend toward 0 as the amount of data increases. The exact form of the SEs depend on how the underlying statistic is modeled. In this work we use the following common definitions *uniformly minimum variance unbiased* (UMVU) estimators for a distribution with mean (μ), variance (V), and standard deviation (σ):

$$\mathrm{SE}_\mu \approx \frac{\sigma_\mu}{\sqrt{n}}\,, \qquad \mathrm{SE}_V \approx \sigma^2\sqrt{\frac{2}{n-1}}\,, \qquad \mathrm{SE}_\sigma \approx \frac{\sigma}{\sqrt{2(n-1)}}\,, \tag{2.1}$$

where n is the number of samples used to estimate the statistic. Similarly, for computing the correlation (ρ) between two sets of length n data, we can estimate the SE as:

$$\mathrm{SE}_\rho \approx \sqrt{\frac{1-\rho^2}{n-2}}\,. \tag{2.2}$$

These SEs are all defined up to order $\mathcal{O}(1/n)$.

For computing AUCs we are not aware of a canonical *analytic* approximation of the SE. However, we can numerically approximate the uncertainty via Monte Carlo, i.e. we assume cases and controls are Gaussian distributed and randomly sample. As can be seen in 1 this was done for 20 different values of AUCs and 3 different ratios of cases to controls.

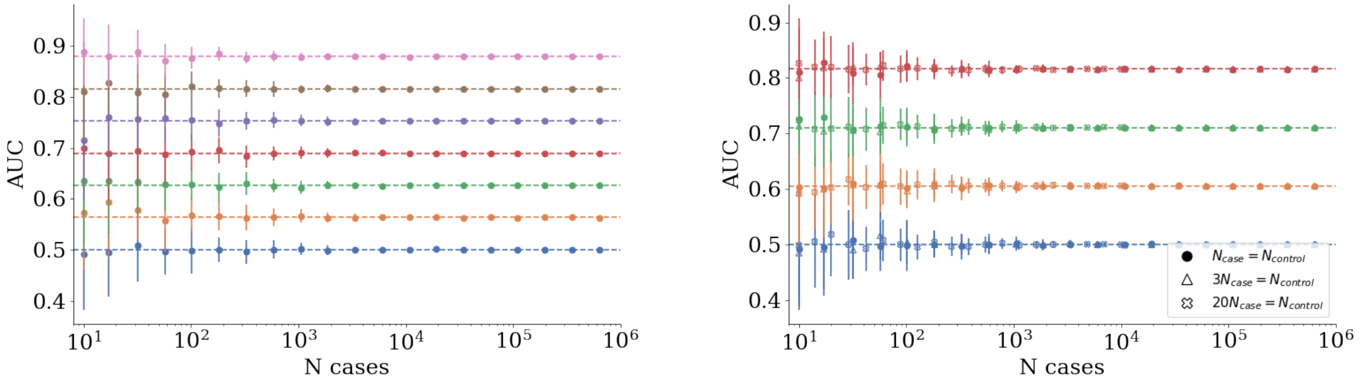

Figure 1: MC AUC error using simulated data. Left, equal cases and controls at different theoretical AUC values. Right, different ratios of cases of controls shows a very weak dependence on the number of controls.

The AUC uncertainty can be fit with a simple polynomial. We find very little dependence on the number of controls, but instead find, after averaging over all 60 draws,

$$\sigma_{AUC} = 0.000_{(0.001)} + 0.30_{(0.09)} N_{\text{case}}^{-0.51_{(0.07)}} \approx \frac{1}{3\sqrt{N_{\text{case}}}}, \tag{2.3}$$

where the right hand side is the approximation we use in the analysis.

2.2 Data Uncertainty

Because understanding the uncertainty on our datapoints is critical to the analysis, especially the projection method, we give some examples here of how error bars/uncertainty was computed.

Odds ratio : the results presented for the LASSO algorithm involve 5-fold cross validation (CV). For each fold, an inclusive odds ratio (OR) is computed. At a given PGS value we can count how many cases have that PGS value or greater, n, how many controls have that PGS value or greater, N, and the total number of cases, n_0, and total number of controls, N_0. A standard error of the OR can be computed by looking at the log(OR) (for noisy data, this approach can lead to nonphysical OR bounds as the OR is not a symmetric distribution) and we find

$$\sigma_{cnt}^{(i)} = \frac{n^{(i)} N_0^{(i)}}{N^{(i)} n_0^{(i)}} \sqrt{\frac{1}{n^{(i)}} + \frac{1}{N^{(i)}} + \frac{1}{n_0^{(i)}} + \frac{1}{N_0^{(i)}}}, \tag{2.4}$$

where we have added the superscript $\frac{1}{n^{(i)}}$ to indicate this contribution comes from the i-th CV fold. We use the label "*cnt*" to indicate that this quantity depends on the counts of cases and controls themselves. To compute the OR we take the mean over the folds, μ. This average itself has a standard deviation, σ_μ. The total uncertainty associated with μ then has a contribution directly from the distribution of CV values and from the counts themselves,

$$\sigma_{OR} = \sqrt{\sigma_\mu^2 + \frac{1}{n_{cv}} \sum_{i=1}^{n_{cv}} \sigma_{cnt}^{(i)\,2}}, \tag{2.5}$$

where n_{cv} is the number of CV folds. The first term comes from the distribution of OR values over CV folds and the second term is an average uncertainty from the counts of cases and controls.

AUC : computing the uncertainty of an AUC measurement is similar to that for the OR: there is one piece coming from averaging over the CV folds and a second piece that comes from the size of the data sample used. Using eq. (2.3), and the fact that each validation set is the same size, we have

$$\sigma_{AUC} = \sqrt{\sigma_\mu^2 + \frac{1}{9N_{case}}}\,. \tag{2.6}$$

Correlation : the uncertainty is analogous to that computed for AUC but now using eq. (2.2),

$$\sigma_{corr} = \sqrt{\sigma_\mu^2 + \frac{1-\rho^2}{n-2}}\,. \tag{2.7}$$

In all these examples, the second contribution is always dependent on the sample size used to compute the quantity. In practice, the size of this contribution compared to the size of the first contribution can be used as an indicator of whether enough data was used for validation/testing. In the results shown in this work the second term is consistently the smaller contribution indicating that the distribution over folds is the limiting factor.

3 Projection method

The value of any metric will depend on the number of samples (cases and controls, or total number for continuous phenotype) used in training *and* validation/testing. It is not *a priori* known what functional form various metrics should take as a function of training samples. From a biological perspective it is true whatever function used should be bounded from above and below, i.e., there should be a lower limit to reflect that linear SNP genetics is playing no role and an upper limit to reflect that you have completely captured linear genetic effects.

<table>
<tr><th>name</th><th>functional form</th><th>min</th><th>max</th></tr>
<tr><td>sigmoid</td><td>$a + \frac{b}{1+e^{c(x+d)}}$</td><td>$a$</td><td rowspan="4">$a+b$</td></tr>
<tr><td>inverse tangent</td><td>$a + \frac{2b}{\pi}\tan^{-1}\left[c(x+d)\right]$</td><td rowspan="3">$a-b$</td></tr>
<tr><td>error function</td><td>$a + b\,\mathrm{erf}\left[c(x+d)\right]$</td></tr>
<tr><td>hyperbolic tangent</td><td>$a + b\tanh\left[c(x+d)\right]$</td></tr>
</table>

Table 1: Various bounded functions used for fitting. Here, x is the $\log_{10}$ of the number of cases included in training. Note that all functions are centered at $x = -d$ which implies that for $x < -d$ there is faster than linear growth in AUC.

Here we collect a group of bounded functions that are used in the main analysis (see **Table 1**). The sigmoid, inverse tangent, and error functions used are all separate, four parameter functions that have left and right finite asymptotes. The hyperbolic tangent can actually be written in terms of the sigmoid, $tanh(x) = 2\Sigma(2x) - 1$, and so it is used as a check that the numerical fitting routine is finding a global minimum. If the routine was getting stuck in a local minimum, the sigmoid and hyperbolic tangent could give separate results. Additionally we checked two other four parameter functions with left and right asymptotes – $a + b(x+d)/\sqrt{c + (x+d)^2}$ and $a + b(x+d)/(c + |x+d|)$ – but these functions have a very sharp increase and do not model well the growth of any of the phenotypes as judged by χ^2 tests.

Fitting is done using the SciPy python package [27], specifically with the non-linear least squares `optimize.curve_fit` function. Because the training sizes span several orders of magnitude, the functions are fit as a function of

function	$\{a,b,c,d\}_{min}$	$\{a,b,c,d\}_{max}$
sigmoid	$\{0.3, 0, -50, -10\}$	$\{0.65, 0.5, 5, -0.5\}$
$\tan^{-1}$,erf,tanh	$\{0.3, -.5, -50, -10\}$	$\{.7, 0.5, 5, -0.5\}$

Table 2: Parameter bounds for the functions listed in **Table 1** and used in the fitting routines.

$log_{10}(N_{train})$. Bounds are provided for all parameters so the "trust region reflective" (trf) algorithm is used. Uncertainty on data points is used for the `sigma` option. Fits are judged via a χ^2 test and reported in **Table 3–Table 13**. While there is no steadfast, *a priori* definition of a "good fit", χ^2 per degree of freedom (χ^2/dof) ≤ 1 is generally considered "good". A p_{value} for this χ^2 statistic is computed as well. $p_{value} \to 0$ indicates possible over-fitting. In cases with a large number of parameters, over-fitting necessitates care must be taken when extrapolating outside the region with data. $p_{value} \to 0$ indicates a poor fit.

As mentioned in the main text, we impose several constraints arising from expected behavior of statistical learning. The first is that the metrics are bounded: $0.5 < \text{AUC} < 1$ and $0 < \text{correlation} < 1$. Positive correlation is merely a convention choice. For the parameters listed in **Table 1** this leads to $a + b < 1$. For the sigmoid, $0.5 < a$ for AUC and $0 < a$ for correlation. For the other functions we have $0.5 < a - b$ for AUC and $0 < a - b$ for correlation. We also know that these metrics should *grow* with training size. This leads to the condition that $c < 0$. Finally we note that this growth should happen with training sizes with large numbers of people, e.g. more than 10. This sets the center of the growth (i.e. function inflection point), d to be negative. To be more conservative, we slightly relax these conditions and use the bounds listed in **Table 2** for the computations.

After the initial fits are found, error bands computed using the Cholesky decomposition method detailed in the main text. Plots demonstrating the full fit results can be found in the main text and in **Figure 2-Figure 6**. After computing the full fits, we can then use the ancestry specific prevalences from literature that we have compiled in section 1.4. Using the prevalences, in combination with biobank sizes (AoU and TPMI), we can estimate the expected numbers of cases in these other biobanks. For continuous measurements we simply use the measurement rate in UKB and assume it will be the same in AoU and TPMI. With these estimated training sizes we can then identify what the various functional forms predict for training in these other biobanks. These results are found in the main text and in **Table 14-Table 24**.

As described in the main text, the correlation of continuous traits with PGS can be used to estimate heritability, h^2_{SNP}, by simply squaring the correlation. We can then use the asymptotic correlation (again, found by simply averaging over the three independent functional forms) to estimate an asymptotic SNP heritability. Conversely, we use GCTA and LDSR results (as detailed in the main text) to estimate SNP heritability. Taking the square root of these estimates gives an expected correlation. These results can be found in **Table 25-Table 26**.

function	Asymp. μ	Asymp. σ	SEM	99% CI of μ	χ^2/dof	p-value
sigmoid4	0.7066	0.0510	0.0007	0.0019	0.72	0.65
arctan4	0.7024	0.0347	0.0005	0.0013	0.95	0.46
erf4	0.7260	0.0680	0.0010	0.0030	0.66	0.71
tanhyp4	0.7062	0.0495	0.0007	0.0018	0.72	0.65

Table 3: (left) Asymptotic central values, and uncertainty, from fitted curves for asthma. (right) Fit results. χ^2 and p_{value} are approximated to two significant digits.

function	Asymp. μ	Asymp. σ	SEM	99% CI of μ	χ^2/dof	p-value
sigmoid4	0.832	0.099	0.005	0.013	0.71	0.66
arctan4	0.815	0.105	0.004	0.011	0.59	0.76
erf4	0.821	0.104	0.004	0.011	0.78	0.60
tanhyp4	0.820	0.102	0.004	0.011	0.73	0.65

Table 4: Asymptotic central values, and uncertainty, from fitted curves for atrial fibrillation. Fit results for atrial fibrillation. χ^2 and p_{value} are approximated to two significant digits.

function	Asymp. μ	Asymp. σ	SEM	99% CI of μ	χ^2/dof	p-value
sigmoid4	0.831	0.099	0.005	0.012	2.16	0.03
arctan4	0.832	0.101	0.004	0.010	2.03	0.05
erf4	0.828	0.095	0.003	0.009	2.23	0.03
tanhyp4	0.823	0.102	0.004	0.009	2.18	0.03

Table 5: Asymptotic central values, and uncertainty, from fitted curves for type 2 diabetes. Fit results for type 2 diabetes. χ^2 and p_{value} are approximated to two significant digits.

function	Asymp. μ	Asymp. σ	SEM	99% CI of μ	χ^2/dof	p-value
sigmoid4	0.6640	0.0074	0.0001	0.0003	0.18	0.99
arctan4	0.6755	0.0134	0.0002	0.0005	0.12	1.00
erf4	0.6634	0.0073	0.0001	0.0003	0.21	0.98
tanhyp4	0.6637	0.0076	0.0001	0.0003	0.18	0.99

Table 6: Asymptotic central values, and uncertainty, from fitted curves for type 1 diabetes. Fit results for type 1 diabetes. χ^2 and p_{value} are approximated to two significant digits.

function	Asymp. μ	Asymp. σ	SEM	99% CI of μ	χ^2/dof	p-value
sigmoid4	0.821	0.107	0.005	0.013	1.65	0.12
arctan4	0.815	0.111	0.004	0.010	1.70	0.10
erf4	0.821	0.102	0.004	0.009	1.63	0.12
tanhyp4	0.824	0.104	0.004	0.009	1.65	0.12

Table 7: Asymptotic central values, and uncertainty, from fitted curves for CAD. Fit results for CAD. χ^2 and p_{value} are approximated to two significant digits.

function	Asymp. μ	Asymp. σ	SEM	99% CI of μ	χ^2/dof	p-value
sigmoid4	0.6317	0.0093	0.0001	0.0003	2.34	0.02
arctan4	0.6434	0.0084	0.0001	0.0003	2.30	0.02
erf4	0.6299	0.0102	0.0001	0.0004	2.47	0.02
tanhyp4	0.6321	0.0095	0.0001	0.0003	2.34	0.02

Table 8: Asymptotic central values, and uncertainty, from fitted curves for hypertension. Fit results for hypertension. χ^2 and p_{value} are approximated to two significant digits.

function	Asymp. μ	Asymp. σ	SEM	99% CI of μ	χ^2/dof	p-value
sigmoid4	0.748	0.104	0.002	0.005	0.84	0.56
arctan4	0.769	0.098	0.002	0.004	0.89	0.51
erf4	0.746	0.098	0.002	0.005	0.82	0.57
tanhyp4	0.747	0.099	0.002	0.005	0.84	0.56

Table 9: Asymptotic central values, and uncertainty, from fitted curves for breast cancer. Fit results for breast cancer. χ^2 and p_{value} are approximated to two significant digits.

function	Asymp. μ	Asymp. σ	SEM	99% CI of μ	χ^2/dof	p-value
sigmoid4	0.5226	0.0274	0.0004	0.0010	0.64	0.72
arctan4	0.5249	0.0240	0.0003	0.0009	0.63	0.73
erf4	0.5279	0.0324	0.0005	0.0013	0.65	0.72
tanhyp4	0.5218	0.0270	0.0004	0.0010	0.64	0.72

Table 10: Asymptotic central values, and uncertainty, from fitted curves for direct bilirubin. Fit results for direct bilirubin. χ^2 and p_{value} are approximated to two significant digits.

function	Asymp. μ	Asymp. σ	SEM	99% CI of μ	χ^2/dof	p-value
sigmoid4	0.4553	0.0246	0.0003	0.0009	0.20	0.98
arctan4	0.4836	0.0180	0.0003	0.0007	1.11	0.35
erf4	0.4669	0.0332	0.0005	0.0012	0.18	0.99
tanhyp4	0.4554	0.0252	0.0004	0.0009	0.20	0.98

Table 11: Asymptotic central values, and uncertainty, from fitted curves for BMI. Fit results for BMI. χ^2 and p_{value} are approximated to two significant digits.

function	Asymp. μ	Asymp. σ	SEM	99% CI of μ	χ^2/dof	p-value
sigmoid4	0.66702	0.00618	0.00009	0.00022	1.48	0.1700
arctan4	0.75880	0.00990	0.00010	0.00040	4.43	0.0001
erf4	0.65413	0.00550	0.00008	0.00020	0.99	0.4400
tanhyp4	0.66715	0.00609	0.00009	0.00022	1.48	0.1700

Table 12: Asymptotic central values, and uncertainty, from fitted curves for height. Fit results for height. χ^2 and p_{value} are approximated to two significant digits.

function	Asymp. μ	Asymp. σ	SEM	99% CI of μ	χ^2/dof	p-value
sigmoid4	0.7721	0.0100	0.0001	0.0004	0.13	1.0
arctan4	0.7992	0.0192	0.0003	0.0007	0.12	1.0
erf4	0.7690	0.0082	0.0001	0.0003	0.14	1.0
tanhyp4	0.7722	0.0101	0.0001	0.0004	0.13	1.0

Table 13: Asymptotic central values, and uncertainty, from fitted curves for lipoprotein A.

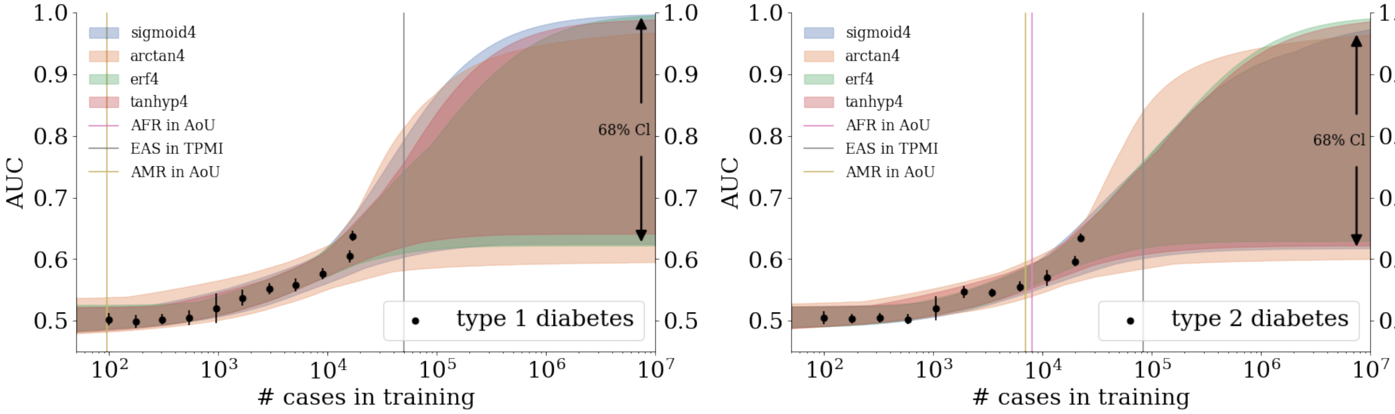

Figure 2: Growth of AUC as a function of training size in the UKB for diabetes type 1 and diabetes type 2. Colored, curved bands come from fitting data with various 4 parameter functions. Width of the bands corresponds to a $\sim 68\%$, or 2 standard deviations, confidence interval on the predictions. Vertical bars represent projections for de novo training in other biobanks using literature prevalences.

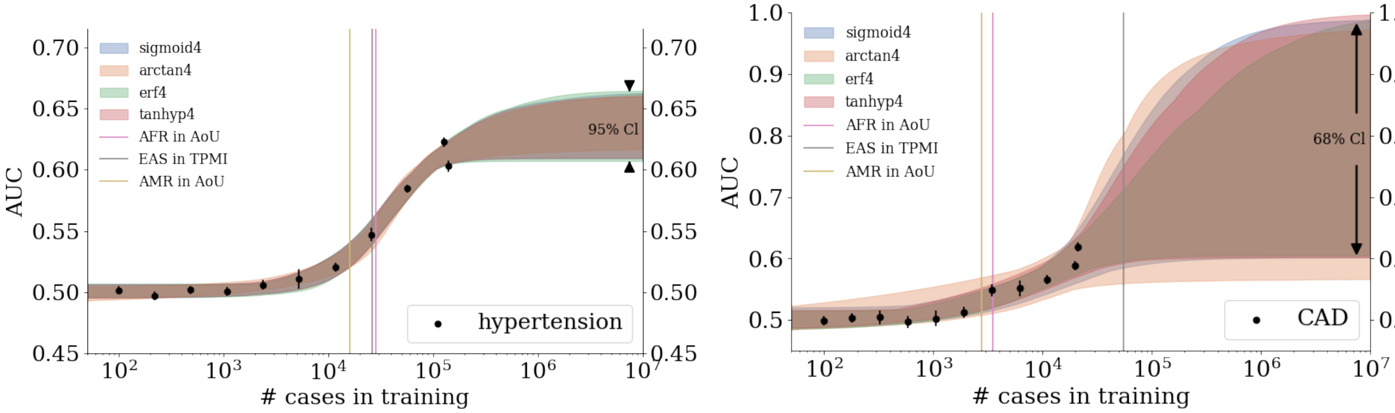

Figure 3: Growth of AUC as a function of training size in the UKB for hypertension and coronary artery disease. Colored, curved bands come from fitting data with various 4 parameter functions. Width of the band corresponds to a confidence interval on the predictions: on the right 2 standard deviations or $\sim 68\%$ and on the left 4 standard deviations or $\sim 95\%$. Vertical bars represent projections for de novo training in other biobanks using literature prevalences.

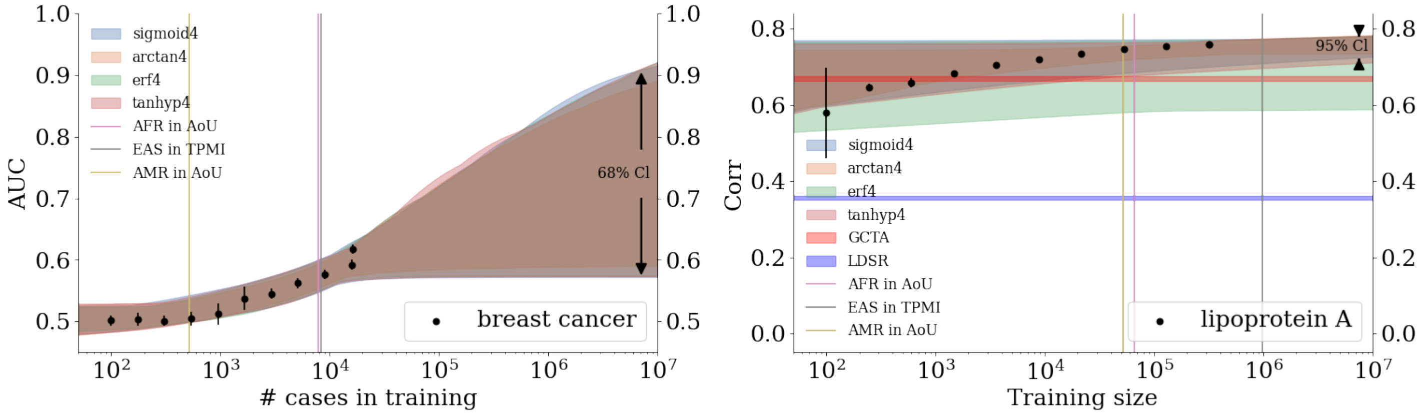

Figure 4: Growth of AUC (left: breast cancer) and correlation (right: lipoprotein A) as a function of training size in the UKB. Colored, curved bands come from fitting data with various 4 parameter functions. Width of the band corresponds to a confidence interval on the predictions: on the left 2 standard deviations or ∼ 68% and on the right 4 standard deviations or ∼ 95%. Vertical bars represent projections for de novo training in other biobanks using literature prevalences. On the right, horizontal lines indicate the correlation predicted from GCTA and LDSR.

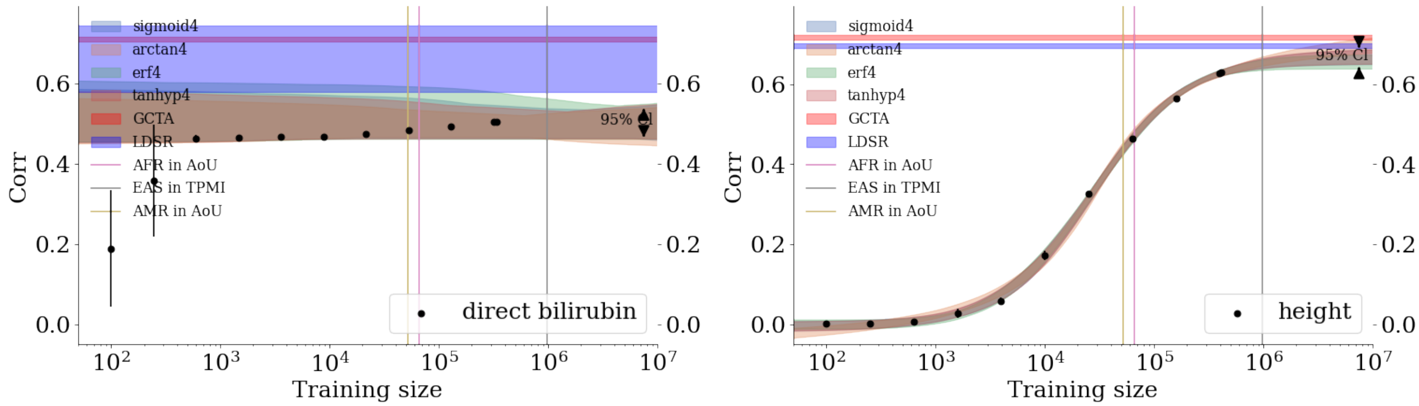

Figure 5: Growth of correlation as a function of training size in the UKB for direct bilirubin and height. Colored, curved bands come from fitting data with various 4 parameter functions. Width of the band corresponds to a ∼ 95% confidence interval, or 4 standard deviations. Vertical bars represent projections for de novo training in other biobanks using literature prevalences. Horizontal lines indicate the correlation predicted from GCTA and LDSR.

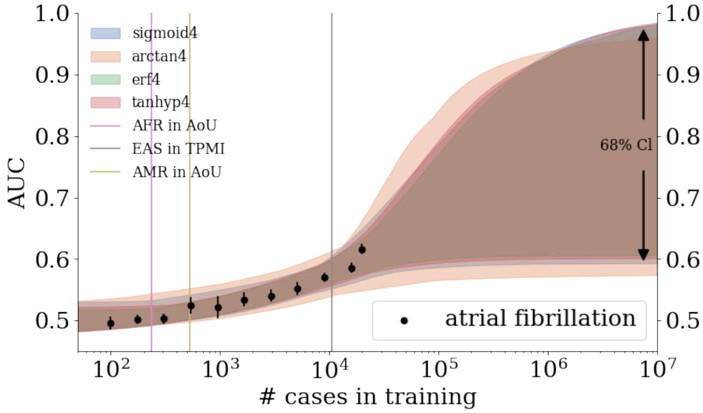

Figure 6: Growth of AUC as a function of training size in the UKB for atrial fibrillation. Colored, curved bands come from fitting data with various 4 parameter functions. Width of the band corresponds to a ∼ 68% confidence interval, or two standard deviations, on the predictions. Vertical bars represent projections for de novo training in other biobanks using literature prevalences.

	sigmoid	σ	arctan	σ	erf	σ	tanh	σ	pred	σ
SIB in UKB	0.61	0.01	0.61	0.01	0.61	0.02	0.61	0.02	0.612	0.009
AFR in AoU	0.54	0.02	0.54	0.01	0.55	0.02	0.54	0.02	0.540	0.010
EAS in TPMI	0.63	0.02	0.63	0.02	0.63	0.03	0.63	0.02	0.630	0.020
AMR in AoU	0.52	0.01	0.52	0.01	0.52	0.02	0.52	0.01	0.520	0.010

Table 14: Various functional predictions, and uncertainties, for asthma.

	sigmoid	σ	arctan	σ	erf	σ	tanh	σ	pred	σ
SIB in UKB	0.62	0.05	0.62	0.07	0.61	0.03	0.61	0.04	0.61	0.03
AFR in AoU	0.51	0.02	0.52	0.03	0.51	0.01	0.51	0.02	0.51	0.01
EAS in TPMI	0.58	0.03	0.58	0.04	0.58	0.02	0.58	0.02	0.58	0.02
AMR in AoU	0.52	0.02	0.52	0.03	0.51	0.01	0.51	0.01	0.52	0.01

Table 15: Various functional predictions, and uncertainties, for atrial fibrillation.

	sigmoid	σ	arctan	σ	erf	σ	tanh	σ	pred	σ
SIB in UKB	0.62	0.04	0.62	0.05	0.62	0.03	0.62	0.04	0.62	0.02
AFR in AoU	0.57	0.02	0.57	0.03	0.57	0.02	0.57	0.02	0.57	0.01
EAS in TPMI	0.68	0.08	0.70	0.10	0.69	0.07	0.68	0.07	0.69	0.06
AMR in AoU	0.56	0.02	0.57	0.03	0.57	0.02	0.57	0.02	0.57	0.01

Table 16: Various functional predictions, and uncertainties, for type 2 diabetes.

	sigmoid	σ	arctan	σ	erf	σ	tanh	σ	pred	σ
SIB in UKB	0.67	0.02	0.67	0.03	0.66	0.02	0.66	0.02	0.67	0.02
AFR in AoU	0.63	0.03	0.63	0.03	0.63	0.03	0.63	0.03	0.63	0.02
EAS in TPMI	0.67	0.03	0.67	0.03	0.66	0.02	0.67	0.02	0.67	0.02
AMR in AoU	0.50	0.10	0.50	0.10	0.52	0.09	0.50	0.10	0.52	0.06

Table 17: Various functional predictions, and uncertainties, for type 1 diabetes.

	sigmoid	σ	arctan	σ	erf	σ	tanh	σ	pred	σ
SIB in UKB	0.61	0.05	0.61	0.06	0.61	0.03	0.61	0.04	0.61	0.03
AFR in AoU	0.53	0.02	0.54	0.03	0.54	0.02	0.54	0.02	0.54	0.01
EAS in TPMI	0.68	0.09	0.70	0.10	0.65	0.06	0.67	0.08	0.67	0.05
AMR in AoU	0.53	0.02	0.54	0.03	0.53	0.02	0.53	0.02	0.53	0.01

Table 18: Various functional predictions, and uncertainties, for CAD.

	sigmoid	σ	arctan	σ	erf	σ	tanh	σ	pred	σ
SIB in UKB	0.61	0.01	0.610	0.010	0.62	0.01	0.62	0.01	0.615	0.006
AFR in AoU	0.55	0.01	0.550	0.010	0.55	0.01	0.55	0.01	0.551	0.006
EAS in TPMI	0.55	0.01	0.540	0.010	0.55	0.01	0.55	0.01	0.546	0.006
AMR in AoU	0.53	0.01	0.528	0.009	0.53	0.01	0.53	0.01	0.528	0.005

Table 19: Various functional predictions, and uncertainties, for hypertension.

	sigmoid	σ	arctan	σ	erf	σ	tanh	σ	pred	σ
EUR/SIB in UKB	0.58	0.03	0.58	0.02	0.58	0.02	0.58	0.03	0.58	0.01
AFR in AoU	0.57	0.03	0.57	0.02	0.57	0.02	0.57	0.03	0.57	0.01
EAS in TPMI	0.57	0.03	0.58	0.02	0.57	0.02	0.58	0.03	0.57	0.01
AMR in AoU	0.52	0.02	0.52	0.02	0.52	0.02	0.52	0.02	0.52	0.01

Table 20: Various functional predictions, and uncertainties, for breast cancer.

	sigmoid	σ	arctan	σ	erf	σ	tanh	σ	pred	σ
SIB in UKB	0.50	0.04	0.49	0.03	0.52	0.06	0.50	0.04	0.50	0.02
AFR in AoU	0.51	0.05	0.50	0.03	0.53	0.07	0.51	0.05	0.51	0.03
EAS in TPMI	0.50	0.04	0.49	0.03	0.51	0.05	0.50	0.04	0.50	0.02
AMR in AoU	0.51	0.05	0.50	0.03	0.53	0.07	0.51	0.05	0.51	0.03

Table 21: Various functional predictions, and uncertainties, for direct bilirubin.

	sigmoid	σ	arctan	σ	erf	σ	tanh	σ	pred	σ
SIB in UKB	0.36	0.01	0.36	0.01	0.36	0.01	0.36	0.01	0.36	0.01
AFR in AoU	0.16	0.01	0.16	0.02	0.17	0.01	0.16	0.01	0.16	0.01
EAS in TPMI	0.40	0.03	0.39	0.02	0.40	0.03	0.40	0.03	0.40	0.02
AMR in AoU	0.14	0.01	0.13	0.01	0.14	0.01	0.14	0.01	0.14	0.01

Table 22: Various functional predictions, and uncertainties, for BMI.

	sigmoid	σ	arctan	σ	erf	σ	tanh	σ	pred	σ
SIB in UKB	0.631	0.008	0.63	0.008	0.631	0.008	0.631	0.008	0.631	0.006
AFR in AoU	0.470	0.010	0.48	0.010	0.470	0.010	0.470	0.010	0.470	0.008
EAS in TPMI	0.650	0.010	0.65	0.010	0.650	0.010	0.650	0.010	0.648	0.009
AMR in AoU	0.430	0.010	0.44	0.010	0.430	0.010	0.430	0.010	0.434	0.009

Table 23: Various functional predictions, and uncertainties, for height.

	sigmoid	σ	arctan	σ	erf	σ	tanh	σ	pred	σ
SIB in UKB	0.73	0.03	0.75	0.02	0.68	0.09	0.73	0.04	0.72	0.03
AFR in AoU	0.72	0.04	0.74	0.02	0.67	0.09	0.72	0.04	0.71	0.03
EAS in TPMI	0.74	0.03	0.75	0.02	0.68	0.09	0.74	0.04	0.73	0.03
AMR in AoU	0.72	0.04	0.73	0.02	0.67	0.09	0.72	0.04	0.71	0.03

Table 24: Various functional predictions, and uncertainties, for lipoprotein A.

	direct bilirubin				BMI			
	Corr	σ	h^2_{SNP}	σ	Corr	σ	h^2_{SNP}	σ
GCTA	0.660	0.080	0.40	0.40	0.499	0.001	0.249	0.008
LDSR	0.711	0.006	0.51	0.02	0.540	0.003	0.290	0.020
Asymp	0.520	0.010	0.27	0.01	0.460	0.020	0.210	0.020

Table 25: Comparison of known correlation and heritability methods to the asymptotic prediction for direct bilirubin (left) and BMI (right).

	height				lipoprotein A			
	Corr	σ	h^2_{SNP}	σ	Corr	σ	h^2_{SNP}	σ
GCTA	0.697	0.005	0.49	0.02	0.356	0.006	0.13	0.09
LDSR	0.717	0.006	0.51	0.02	0.670	0.005	0.45	0.02
Asymp	0.663	0.003	0.439	0.005	0.779	0.007	0.61	0.01

Table 26: Comparison of known correlation and heritability methods to the asymptotic prediction for height (left) and lipoprotein A (right).

4 Sparse methods comparisons on various phenotypes

The main text details the use and comparison of various sparse methods for asthma and height. Here, in **Figure 7-Figure 11** we include the remaining phenotypes. Solid bars for AFR, EAS, and AMR come from the projection method described in section 3 and in the main text.

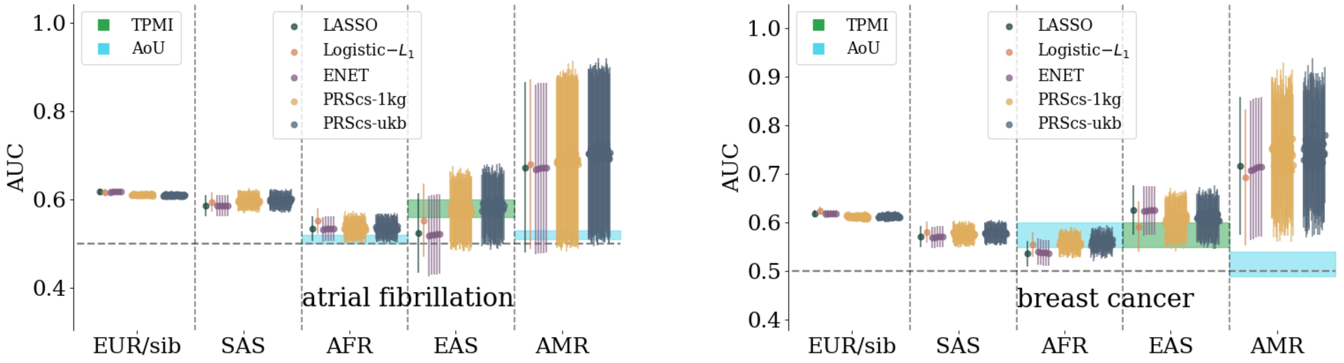

Figure 7: Comparison of sparse methods for atrial fibrillation and breast cancer predictors with a comparison to prediction bands for more diverse biobanks. Both trait predictors are trained on a UKB white population. Predictors are built with LASSO, L_1-penalized Logistic regression, Elastic Net, and PRScs with UKB and 1,000 Genomes LD matrices. The specific parameters for the Elastic Net and PRScs are described in the methods section.

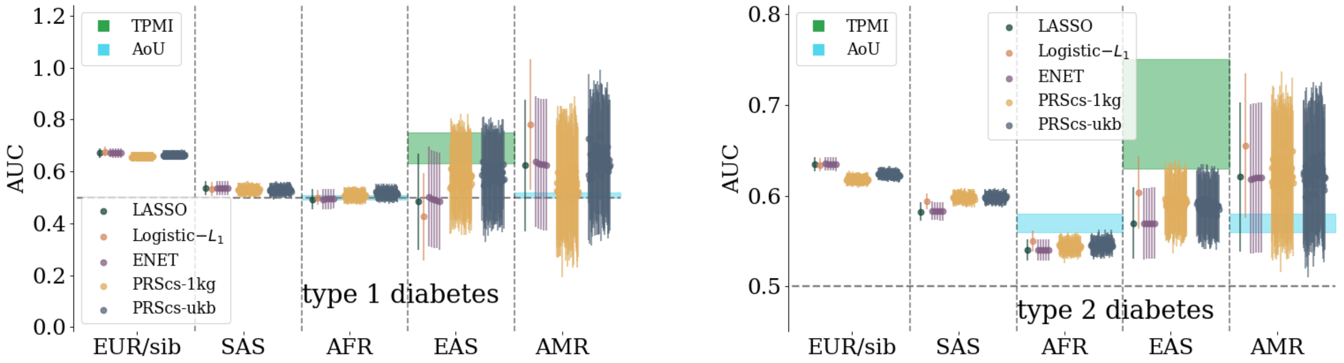

Figure 8: Comparison of sparse methods for type 1 diabetes and type 2 diabetes predictors with a comparison to prediction bands for more diverse biobanks. Both trait predictors are trained on a UKB white population. Predictors are built with LASSO, L_1-penalized Logistic regression, Elastic Nets, and PRScs with UKB and 1,000 Genomes LD matrices. The specific parameters for the Elastic nets and PRScs are described in the methods section.

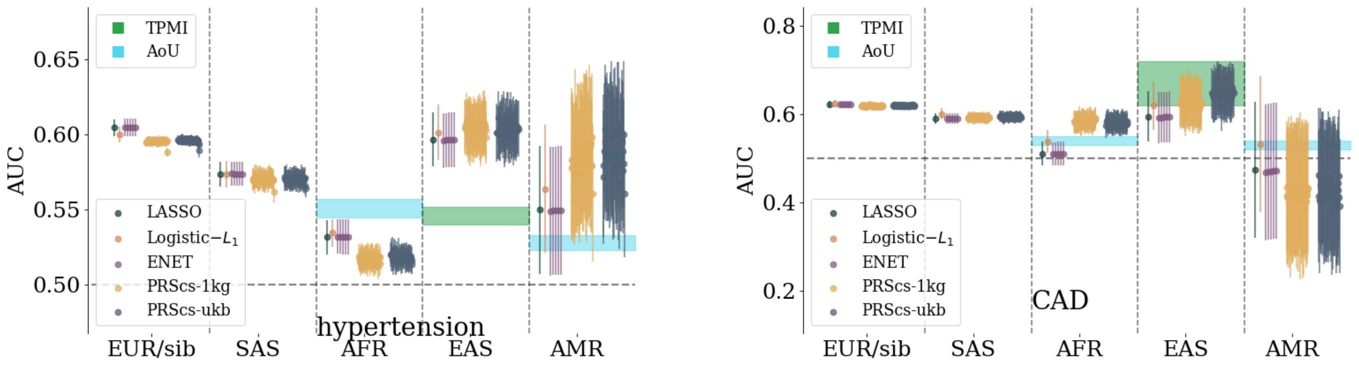

Figure 9: Comparison of sparse methods for hypertension and coronary artery disease predictors with a comparison to prediction bands for more diverse biobanks. Both trait predictors are trained on a UKB white population. Predictors are built with LASSO, L_1-penalized Logistic regression, Elastic Nets, and PRScs with UKB and 1,000 Genomes LD matrices. The specific parameters for the Elastic nets and PRScs are described in the methods section.

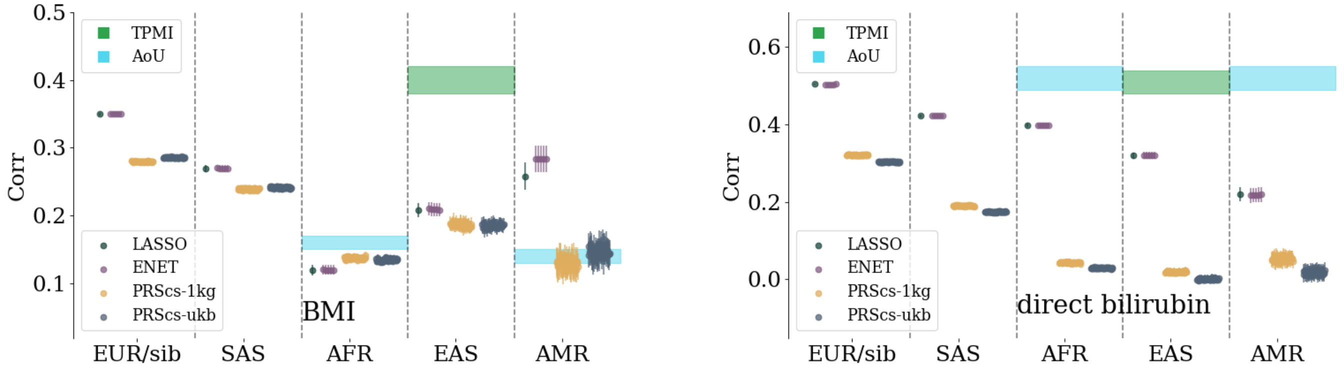

Figure 10: Comparison of sparse methods for BMI and direct bilirubin predictors with a comparison to prediction bands for more diverse biobanks. Both trait predictors are trained on a UKB white population. Predictors are built with LASSO, L_1-penalized Logistic regression, Elastic Nets, and PRScs with UKB and 1,000 Genomes LD matrices. The specific parameters for the Elastic nets and PRScs are described in the methods section.

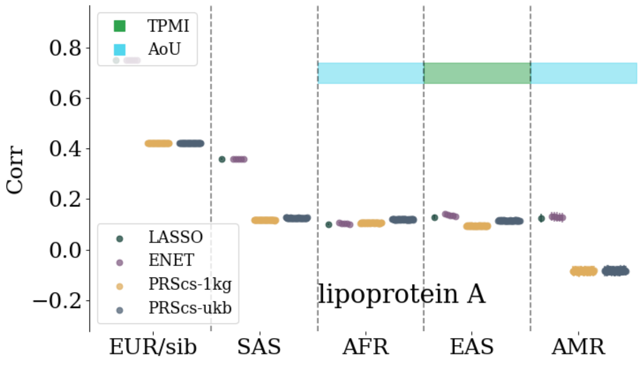

Figure 11: Comparison of sparse methods for lipoprotein A predictors with a comparison to prediction bands for more diverse biobanks. The trait predictors are trained on a UKB white population. Predictors are built with LASSO, L_1-penalized Logistic regression, Elastic Nets, and PRScs with UKB and 1,000 Genomes LD matrices. The specific parameters for the Elastic nets and PRScs are described in the methods section.

5 Sibling Tests

Sibling selection tests are described in the main text and presented there for asthma and BMI. Sibling tests for the remaining phenotypes are included here in **Figure 12-Figure 16**.

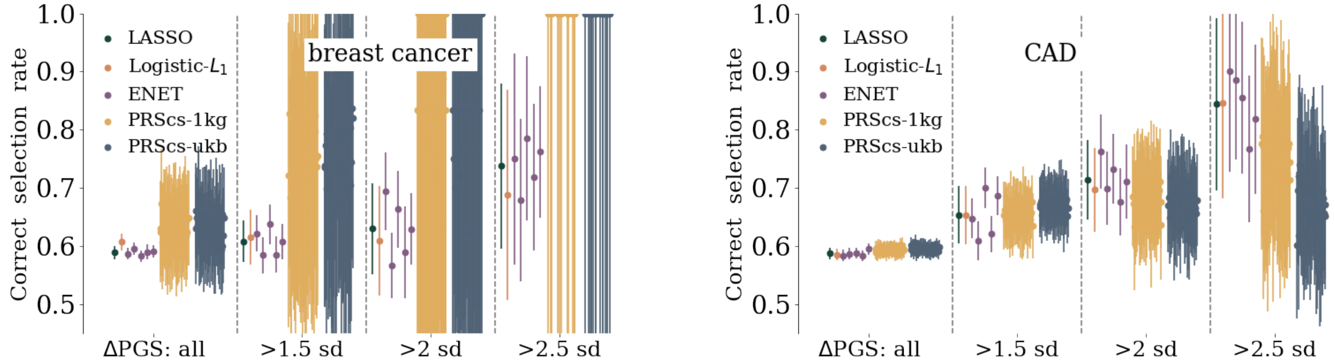

Figure 12: Affected sibling pair (ASP) selection rate for breast cancer and coronary artery disease. Pairs of siblings, where one person is a case and the other a control, are used and the rate corresponds to the number of times the case sibling has the higher PGS. The rate of correct selection, and uncertainty, increases if the siblings are also separated by at least 1.5, 2, or 2.5 standard deviations in PGS.

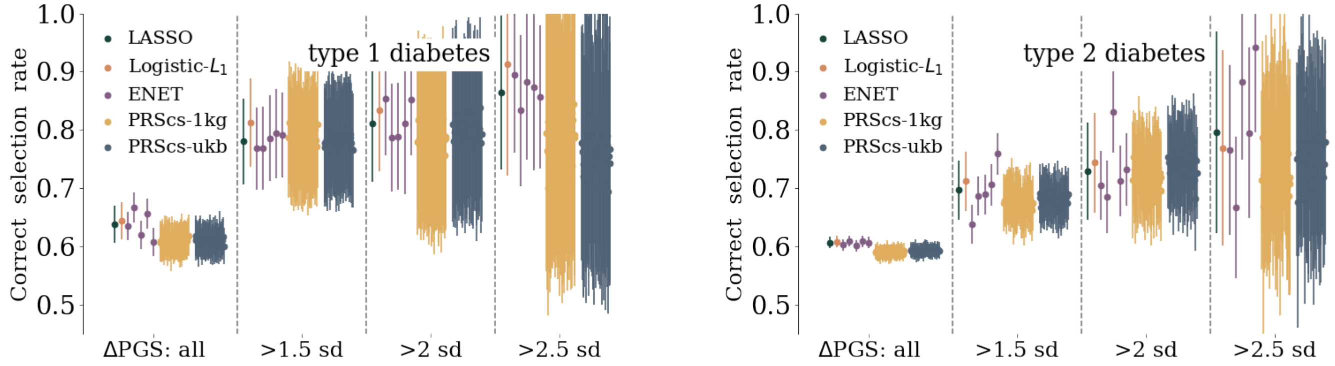

Figure 13: Affected sibling pair (ASP) selection rate for type 1 diabetes and type 2 diabetes. Pairs of siblings, where one person is a case and the other a control, are used and the rate corresponds to the number of times the case sibling has the higher PGS. The rate of correct selection, and uncertainty, increases if the siblings are also separated by at least 1.5, 2, or 2.5 standard deviations in PGS.

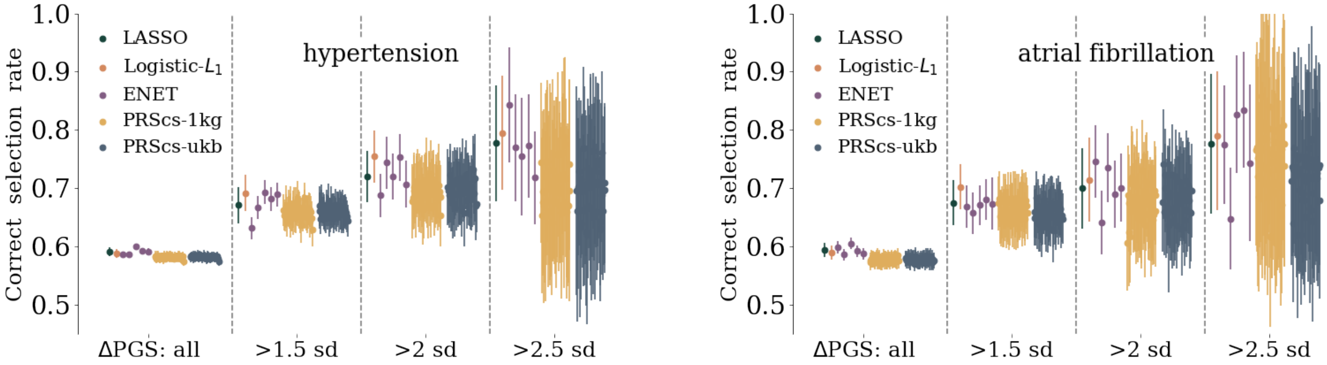

Figure 14: Affected sibling pair (ASP) selection rate for hypertension and atrial fibrillation. Pairs of siblings, where one person is a case and the other a control, are used and the rate corresponds to the number of times the case sibling has the higher PGS. The rate of correct selection, and uncertainty, increases if the siblings are also separated by at least 1.5, 2, or 2.5 standard deviations in PGS.

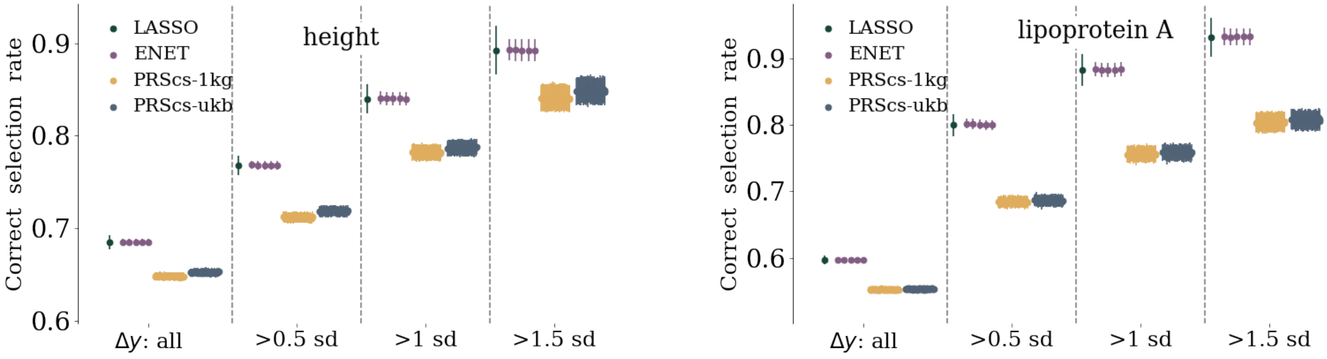

Figure 15: Rank order selection rate for height and lipoprotein A. The rate corresponds to frequency of the sibling with the larger phenotype also having the larger PGS. The selection rate, and uncertainty, increase if you require that the siblings phenotype is separated by at least 0.5, 1, or 1.5 standard deviations.

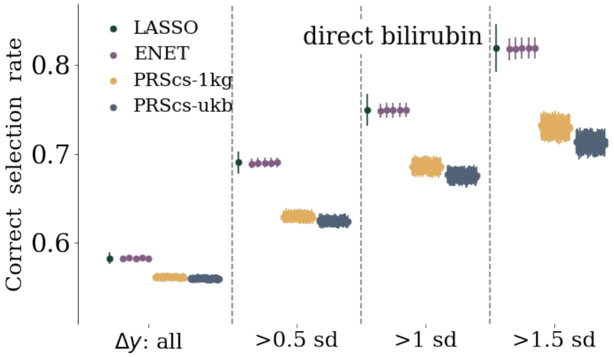

Figure 16: Rank order selection rate for direct bilirubin. The rate corresponds to frequency of the sibling with the larger phenotype also having the larger PGS. The selection rate, and uncertainty, increase if you require that the siblings phenotype is separated by at least 0.5, 1, or 1.5 standard deviations.

6 Impact Regions

Here we collect, in table form, the most impactful regions for LASSO predictors for each phenotype. The impactful SNPs are found by looking at SSV for each SNP. Associated genes are identified by using `www.ncbi.nlm.nih.gov/gene/` gene database, for primary build GRCh37, and extending the gene region by 2 million base pairs in both directions to capture possibly associated SNPs.

SNP	Chromo- -some	GRCh37 position	# of CV folds	Average gwas p-val	Near known assoc. genes	SNP	Chromo- -some	GRCh37 position	# of CV folds	Average gwas p-val	Near known assoc. genes
Affx-35292235	1	12,175,658	4	1.8E-07		rs146597587	9	6,255,967	4	3.8E-14	IL33
rs496451	1	55,553,568	1	1.7E-03		rs12413578	10	9,049,253	2	3.3E-34	
rs12123821	1	152,179,152	2	7.4E-23	FLG	rs144497064	10	75,305,340	3	5.8E-04	
rs150597413	1	152,277,622	3	7.4E-06	FLG	rs145065400	11	6,912,921	1	5.2E-02	
rs138726443	1	152,280,023	2	3.7E-05	FLG	rs11041171	11	7,110,751	1	2.1E-02	
Affx-52112636	1	155,691,351	1	3.5E-04		Affx-35631493	11	48,387,855	1	1.8E-02	
rs112674836	1	169,822,140	1	3.8E-03		rs150131716	11	77,774,947	1	5.9E-03	
rs141099682	1	186,083,113	1	7.0E-03		rs57224706	12	50,480,285	1	1.6E-03	
rs116178033	1	207,751,640	1	2.1E-02		rs1059513	12	57,489,709	1	1.9E-22	
rs35784105	2	7,017,943	1	2.4E-02		rs144880038	14	74,422,599	1	7.3E-03	
rs72823646	2	102,954,213	4	1.7E-60	IL1RL1	rs11071559	15	61,069,988	1	1.6E-15	RORA
rs144641723	2	209,308,236	1	1.7E-03		rs17293632	15	67,442,596	1	2.3E-36	SMAD3
rs189756911	3	24,522,932	1	2.9E-04		rs36045143	16	11,224,966	2	5.5E-27	CLEC16A
rs62323888	4	123,057,411	1	6.6E-12		rs140980200	17	3,840,797	1	4.9E-04	
rs148087185	4	166,978,362	1	1.6E-03		rs144535413	17	3,844,344	1	1.0E-03	
rs116351845	5	44,034,122	1	1.5E-06		rs115065009	17	8,215,755	1	1.3E-02	
rs1837253	5	110,401,872	2	2.1E-37	TSLP	rs75865341	17	55,262,828	1	3.8E-02	
rs3104413	6	32,582,650	1	6.4E-67	HLA-DQ	rs35250503	17	71,160,703	1	7.6E-03	
rs58156640	6	62,472,822	2	6.6E-03		rs59181893	17	76,887,003	4	5.8E-03	
rs4875777	8	3,569,431	1	3.6E-04		rs12964116	18	61,442,619	1	3.0E-08	SERPINB7
rs7827037	8	39,284,717	1	4.2E-03							

Table 27: Asthma SNPs with SSV > 1% of total SSV.

SNP	Chromo- -some	GRCh37 position	# of CV folds	Average gwas p-val	Near known assoc. genes	SNP	Chromo- -some	GRCh37 position	# of CV folds	Average gwas p-val	Near known assoc. genes
rs61730486	1	117,146,319	1	2.5E-03		rs150511464	7	127,235,385	1	6.7E-03	
rs13376333	1	154,814,353	1	2.7E-29	KCNN3 PMVK LMNA	rs149775276	8	42,587,435	1	3.2E-03	
rs140707188	1	156,169,862	1	2.6E-03	KCNN3 PMVK LMNA	rs16938829	8	75,157,149	1	5.2E-03	
rs6011	1	169,500,178	2	5.0E-04	KIFAP3	rs62521286	8	124,551,975	1	2.4E-10	FBXO32
rs72700121	1	170,195,607	2	1.9E-19	KIFAP3	rs112892337	8	135,614,553	1	2.8E-04	
rs1332965	1	170,283,751	2	1.0E-08		rs116352541	9	126,144,262	1	4.7E-03	
rs145618145	2	63,220,672	1	2.2E-02		rs6584555	10	105,299,611	2	4.7E-17	SH3PXD2A
rs35955389	2	173,825,951	1	4.7E-02		rs75053469	10	105,351,996	1	3.2E-09	SH3PXD2A
rs10176633	2	188,361,675	1	4.3E-03		rs79307606	11	132,861,210	2	4.4E-05	
rs146955672	3	24,200,787	1	2.3E-03		rs139258361	12	15,803,861	1	2.4E-03	
rs17079534	3	39,847,072	1	2.5E-02	SCN5A SCN10A	rs112129104	12	89,752,523	1	1.3E-05	
rs11574440	3	46,449,164	1	5.5E-02		rs189018671	13	97,639,878	1	5.8E-03	
rs2942857	4	69,687,987	1	1.2E-02		rs6576507	15	26,288,394	3	2.8E-03	
rs2200733	4	111,710,169	1	1.8E-140	PITX2	rs7164883	15	73,652,174	1	1.7E-13	HCN4
rs6843082	4	111,718,067	4	1.8E-160	PITX2	rs138423783	15	81,180,069	1	4.6E-03	
rs3853445	4	111,761,487	1	1.1E-32	PITX2	rs11858667	15	88,022,696	1	1.3E-02	
rs111334323	4	111,910,151	3	4.0E-06	PITX2	rs140703991	16	1,820,683	2	6.6E-05	
rs200747616	4	152,550,918	2	2.4E-03		rs147972626	16	1,997,064	1	1.1E-05	
rs138408376	5	140,801,426	1	3.8E-03		rs140185678	16	2,003,016	3	3.7E-14	RPL
rs114183232	6	1,401,166	1	7.4E-04		rs143944741	16	19,883,375	1	3.5E-03	
rs16893699	6	28,105,889	1	4.2E-03		rs2106261	16	73,051,620	3	2.2E-35	ZFHX3
rs181511246	6	75,848,630	1	1.7E-04		rs2649402	17	18,566,055	1	4.8E-03	
rs117984853	6	149,399,100	1	5.0E-14		rs111681839	17	79,176,142	1	3.9E-02	
rs7807549	7	43,917,465	1	5.5E-03		rs61731848	19	9,060,774	1	5.4E-03	
rs2229107	7	87,138,659	1	6.0E-03		rs10425706	19	53,741,219	1	3.8E-03	
rs3807989	7	116,186,241	1	1.7E-18	CAV1	rs115547861	22	31,032,704	1	1.4E-03	

Table 28: Atrial fibrillation SNPs with SSV > 1% of total SSV.

SNP	Chromo-some	GRCh37 position	# of CV folds	Average gwas p-val	Near known assoc. genes
rs77316894	1	90,804,699	1	1.4E-05	
rs138421943	1	152,192,110	3	9.8E-04	
rs150343459	2	54,021,461	4	1.1E-04	
rs72951831	2	217,957,699	2	1.4E-12	
rs7575022	2	237,964,265	2	1.2E-03	
rs74371893	3	4,345,484	3	1.6E-03	
rs76997204	3	20,027,251	1	1.9E-03	
rs75783758	4	137,761,414	1	1.3E-03	
rs148833559	5	172,755,066	2	9.3E-05	
rs16893699	6	28,105,889	4	1.5E-05	
rs2841646	6	43,270,326	1	1.6E-03	
rs41302073	9	12,709,125	1	1.7E-03	
rs3780548	9	99,525,487	2	8.6E-06	
rs74541872	9	116,798,596	4	5.4E-03	
rs1219648	10	123,346,190	1	7.5E-100	
rs2295878	10	123,996,934	1	8.2E-03	
rs7943891	11	48,843,790	1	8.2E-03	
rs75296154	11	69,343,815	2	9.3E-22	
rs151013524	11	134,062,648	1	3.7E-03	
rs3803466	15	75,648,650	1	1.7E-03	
rs75531903	16	3,613,720	2	8.5E-03	
rs4784227	16	52,599,188	1	2.3E-69	
rs61757659	16	88,951,594	1	7.1E-05	
rs16989263	21	33,694,195	4	5.0E-04	

Table 29: Breast cancer SNPs with SSV > 1% of total SSV.

SNP	Chromo-some	GRCh37 position	# of CV folds	Average gwas p-val	Near known assoc. genes
rs74066236	1	37,919,465	2	5.5E-03	
rs156258	1	45,042,530	1	3.4E-03	
rs116473331	1	50,486,231	3	7.5E-05	
rs139971696	1	150,935,216	1	1.3E-02	
rs145020579	1	223,972,058	2	2.1E-03	
rs79802002	1	247,654,493	1	1.1E-01	
rs200225276	2	27,262,648	2	3.9E-05	GCKR
rs143491198	2	27,695,208	2	8.8E-04	GCKR
rs6715188	2	125,359,946	1	8.6E-03	
rs78499613	2	210,705,365	2	5.7E-05	
rs72628104	3	24,006,497	1	2.7E-02	
rs150271072	3	57,631,400	1	2.1E-04	
rs146206905	3	120,347,344	2	3.2E-04	
rs16837181	3	125,878,980	1	1.4E-04	
rs61753468	3	129,156,151	1	5.1E-04	
rs144574896	4	22,456,492	1	3.0E-04	
rs75978835	4	120,501,178	1	4.1E-03	
rs146886108	5	14,751,305	2	9.2E-08	
rs141074846	5	132,209,649	2	1.0E-03	
rs115673052	6	83,106,667	1	2.8E-02	
rs3802177	8	118,185,025	1	3.7E-21	SLC30A8
Affx-52298270	9	87,338,515	1	4.9E-03	
rs7074231	10	3,304,976	1	7.9E-03	
rs75106042	10	114,651,604	1	1.4E-07	TCF7L2
rs7903146	10	114,758,349	3	4.4E-143	TCF7L2
rs34796596	11	67,414,455	4	1.1E-03	
rs61001398	12	22,040,823	1	2.6E-03	
rs139495835	12	57,642,464	1	4.7E-02	
rs73380117	12	100,346,464	2	7.3E-03	
rs79111014	13	43,463,378	1	2.3E-03	
rs35716003	14	22,580,806	1	8.8E-03	
rs143602956	14	101,201,112	1	9.3E-03	
rs74553953	15	49,048,132	2	1.4E-05	SLC12A1
rs34437030	16	29,859,305	1	8.9E-03	
rs6500304	16	48,134,784	1	2.6E-03	
rs6500305	16	48,134,856	2	2.8E-03	
rs35840072	16	83,288,120	2	1.3E-04	
rs149760662	17	5,291,126	1	5.0E-04	
rs9748611	18	14,763,987	1	3.5E-02	
rs35554127	18	21,736,486	1	3.8E-02	
rs150579280	19	16,006,361	2	6.2E-04	
rs114897412	20	4,162,837	1	8.7E-03	
rs141568926	20	9,434,049	1	1.6E-03	
rs34875296	22	26,423,535	2	3.9E-03	

Table 30: Type 2 diabetes SNPs with SSV > 1% of total SSV

SNP	Chromo-some	GRCh37 position	# of CV folds	Average gwas p-val	Near known assoc. genes
rs17098940	1	76,878,097	1	2.0E-02	
rs6679677	1	114,303,808	1	1.5E-14	
rs6587601	1	149,773,350	2	5.3E-05	
rs16861531	2	14,398,052	1	4.6E-02	
rs7588635	2	112,722,854	1	4.3E-03	
rs116825611	2	167,298,015	1	1.6E-02	
rs192447754	2	207,171,050	1	1.2E-02	
rs76844281	3	24,392,632	1	3.6E-02	
rs199768782	4	15,542,617	1	1.1E-01	
rs1136511	4	70,898,903	1	1.9E-01	
rs2219222	5	114,737,846	1	4.7E-03	
rs116763857	6	31,141,482	3	1.6E-05	HLA-A TRIM26 MICA HLA-DRB1
rs3806155	6	32,373,378	2	4.7E-53	MICA HLA-DRB1
rs9268577	6	32,395,942	1	1.2E-16	MICA HLA-DRB1
rs146733600	6	32,573,760	4	1.1E-45	MICA HLA-DRB1
rs9273363	6	32,626,272	3	1.3E-114	MICA HLA-DRB1
rs115018313	6	32710407	3	1.1E-57	MICA HLA-DRB1
rs115469976	6	32,986,508	1	1.7E-30	MICA HLA-DRB1
rs112222457	6	47,658,337	2	2.2E-02	
rs112609906	6	66,204,970	1	5.2E-02	
rs28382660	7	44,112,996	1	3.8E-02	
rs116376908	7	151,079,054	1	1.4E-01	
rs79108638	9	91,292,717	1	6.6E-02	
rs2702693	11	19,530,851	2	1.0E-01	
rs45438191	11	62,763,544	2	2.9E-03	
rs11837049	12	22,168,032	1	4.1E-04	
rs1669885	12	42,839,836	1	1.4E-02	
rs17592	15	42,678,464	3	2.5E-03	
rs80229418	16	31,412,772	1	6.7E-02	
rs13338753	16	55,903,548	1	8.4E-02	
rs35554127	18	21,736,486	1	5.1E-03	
rs8107847	19	763,508	1	7.4E-03	
rs146265828	20	12,619,601	2	1.3E-02	FLRT3

Table 31: Type 1 diabetes SNPs with SSV > 1% of total SSV.

SNP	Chromo-some	GRCh37 position	# of CV folds	Average gwas p-val	Near known assoc. genes
rs78426182	1	2,519,810	2	9.2E-04	
rs34561376	1	55,464,986	1	1.7E-03	PCSK9
rs11591147	1	55,505,647	2	5.9E-11	PCSK9
rs142460316	1	117,619,377	1	1.3E-04	
rs76443098	1	155,657,890	1	1.2E-02	IL6R
rs76329326	1	222,803,199	1	9.8E-05	MIA3
rs73949680	2	74,756,242	3	1.4E-02	
rs115370220	2	87,016,830	2	1.4E-02	VAMP5
rs79904664	2	144,699,925	3	7.8E-03	ZEB2
rs9827335	3	3,840,868	1	8.8E-03	
rs13326552	3	44,948,480	5	7.1E-06	
rs144662307	3	195,481,129	3	2.1E-04	
rs142412240	4	68,925,094	1	1.7E-04	
rs73786670	5	130,766,542	1	2.1E-02	SLC22A4/A5
rs80263204	5	140,627,509	1	1.3E-02	
rs2120783	5	161,007,916	1	9.8E-04	
rs3734280	6	134,215,690	1	9.6E-03	TCF21
rs3798220	6	160,961,137	2	3.0E-44	SLC22A3 LPAL2 LPA PLG
rs10455872	6	161,010,118	3	1.8E-73	SLC22A3 LPAL2 LPA PLG
rs17847173	7	8,181,578	1	1.7E-04	
rs60659894	8	94,772,183	3	2.2E-02	
rs116930274	8	144,590,049	1	1.2E-03	
rs7126678	11	48,505,913	1	2.0E-01	
rs34796596	11	67,414,455	1	4.4E-02	
rs58168448	12	109,895,854	1	4.9E-02	SH2B3
rs200297509	12	124,835,279	1	7.5E-03	
rs41561818	12	133,220,454	1	3.2E-04	
rs144567652	14	45,667,921	1	7.6E-03	
rs150953383	15	73,994,895	1	3.3E-04	
rs16942445	16	57,935,443	1	6.5E-03	CETP
rs58981829	19	51,738,465	1	2.4E-02	

Table 32: CAD SNPs with SSV > 1% of total SSV.

SNP	Chromo- -some	GRCh37 position	# of CV folds	Average gwas p-val	Near known assoc. genes	SNP	Chromo- -some	GRCh37 position	# of CV folds	Average gwas p-val	Near known assoc. genes
rs41270291	1	15,994,251	1	2.4E-01		rs11016690	10	130,940,735	1	3.3E-01	
rs115862221	1	17,249,858	1	5.1E-02		rs138242314	11	45,955,752	1	1.2E-02	
rs11466111	1	115,829,178	1	9.9E-06		rs8187661	11	66,133,643	1	2.1E-02	
rs145467872	1	150,973,013	1	4.4E-02		rs16914280	11	88,321,724	1	1.8E-01	
rs55909005	1	156,838,432	1	2.5E-01		rs11838918	13	79,410,574	1	3.9E-01	
rs9661539	1	248,040,293	1	1.9E-01		rs7317657	13	92,171,706	1	4.0E-01	
rs45612738	2	31,602,841	1	2.6E-01		rs73296180	14	53,619,369	1	2.2E-02	
rs61731210	3	13,659,649	1	5.3E-01		rs112118955	14	73,996,947	1	1.5E-01	
rs17051692	3	40,931,175	1	1.9E-01	ULK4	rs116488414	14	74,006,004	1	6.6E-02	
rs4234213	3	123,024,733	1	9.1E-02		rs149239345	14	96,761,321	1	1.3E-01	
rs57958890	4	70,276,699	1	2.2E-01		rs6576507	15	26,288,394	1	6.4E-01	
rs143057152	4	149,075,755	1	4.9E-03	NR3C2	rs114231576	16	2,016,184	1	5.9E-02	
rs6915612	6	32,218,625	1	2.8E-01	PRRC2A	rs4889238	16	81,151,122	1	5.1E-02	
rs112460091	6	63,995,477	1	1.0E-01		rs112966915	17	80,223,554	1	1.9E-01	
rs59147126	7	15,601,393	1	2.8E-01		rs150705131	19	288,123	1	3.1E-02	
rs3735533	7	27,245,893	1	4.2E-08		rs61740630	19	2,352,964	1	1.8E-01	
rs115281203	7	123,672,179	1	1.6E-01		rs36078704	19	19,039,030	1	2.3E-01	
rs11977216	7	150,324,976	1	3.2E-01	NOS3	rs73932907	19	38,039,946	1	2.0E-01	
rs116751013	7	158,415,596	1	7.3E-02		rs201635014	19	38,379,680	1	1.2E-01	
rs10958553	8	39,290,593	1	2.9E-01		rs61760904	19	50,139,932	1	1.1E-03	
rs148545964	8	94,772,206	1	5.8E-01		rs55641738	20	60,293,875	1	2.8E-01	
rs3847170	8	140,423,147	1	4.2E-01		rs28587162	21	16,337,538	1	1.5E-01	
rs3025380	9	136,501,756	1	1.4E-03		rs6003859	22	24,057,206	1	2.7E-01	
rs73270587	10	35,929,350	1	1.9E-01		rs28915381	22	43,089,658	1	2.6E-01	

Table 33: Hypertension SNPs with SSV > 1% of total SSV.

SNP	Chromo- -some	GRCh37 position	# of CV folds	Average gwas p-val	Near known assoc. genes	SNP	Chromo- -some	GRCh37 position	# of CV folds	Average gwas p-val	Near known assoc. genes
rs17131137	1	91,172,059	2	3.2E-04		Affx-52351697	12	21,008,080	4	1.1E-39	SLCO1B3 SLCO1B1
rs4019811	1	203,822,304	4	1.8E-05		rs34691116	12	21,027,327	3	2.3E-89	SLCO1B3 SLCO1B1
rs908327	1	235,092,600	4	1.6E-02		rs76737149	12	21,235,850	2	2.0E-16	SLCO1B3 SLCO1B1
rs6755571	2	234,627,536	3	0.0E+00	UGT1A1	rs11045819	12	21,329,813	2	1.6E-28	SLCO1B3 SLCO1B1
rs34622615	2	234,652,308	4	0.0E+00	UGT1A1	rs4149056	12	21,331,549	2	7.9E-308	SLCO1B3 SLCO1B1
rs10929302	2	234,665,782	2	0.0E+00	UGT1A1	rs73117071	12	41,582,272	3	1.2E-03	
rs3755319	2	234,667,582	4	0.0E+00	UGT1A1	rs61742753	16	1,614,147	3	4.3E-05	
rs887829	2	234,668,570	5	0.0E+00	UGT1A1	rs114221795	20	42,086,724	2	1.5E-07	
rs4148323	2	234,669,144	5	6.6E-03	UGT1A1	rs8132639	21	27,012,154	5	2.7E-06	
rs2290189	3	13,677,946	1	1.2E-03		rs16989263	21	33,694,195	2	1.1E-05	
rs142626656	8	41,575,677	2	1.8E-07		rs35946782	21	40,763,754	1	1.9E-03	

Table 34: Direct bilirubin SNPs with SSV > 1% of total SSV.

SNP	Chromo- -some	GRCh37 position	# of CV folds	Average gwas p-val	Near known assoc. genes	SNP	Chromo- -some	GRCh37 position	# of CV folds	Average gwas p-val	Near known assoc. genes
rs201703264	1	3428144	2	4.2E-04		rs77620102	8	21986657	2	2.9E-03	
rs74066236	1	37919465	2	5.5E-03		rs74062407	12	11083647	2	4.9E-03	
rs62106258	2	417167	3	1.2E-70	TMEM18	rs141556486	12	132404616	1	4.3E-05	
rs73019479	2	171627219	1	8.6E-03		rs1421085	16	53800954	2	3.0E-262	FTO
rs4685689	3	3886580	3	5.3E-03		rs143242388	16	70380869	1	9.2E-03	
rs9843741	3	99568737	2	4.7E-04		rs1060250	16	87874736	4	2.5E-03	
rs2871630	3	129372880	1	6.7E-03		rs10405385	19	55856147	1	3.7E-04	
rs72976362	3	133567847	1	7.3E-03		rs112799437	19	58609112	2	7.6E-04	
rs76693682	5	180482838	2	4.1E-03		rs62224618	22	16057417	2	6.9E-01	
rs2229107	7	87138659	2	1.1E-03		rs34379045	22	19511476	3	6.0E-03	

Table 35: BMI SNPs with SSV > 1% of total SSV.

SNP	Chromo- -some	GRCh37 position	# of CV folds	Average gwas p-val	Near known assoc. genes	SNP	Chromo- -some	GRCh37 position	# of CV folds	Average gwas p-val	Near known assoc. genes
rs201823506	1	51,871,568	1	1.4E-03	ORC1	rs35796392	9	129,595,583	2	1.8E-05	
rs10019684	4	69,383,874	1	2.5E-03		rs10987622	9	130,133,619	1	2.3E-03	
rs142228984	5	32,786,413	1	8.9E-09		rs28407189	15	89,400,680	2	3.0E-135	ACAN
rs148833559	5	172,755,066	2	4.9E-29		rs141308595	15	89,424,870	4	9.2E-30	ACAN
rs41271299	6	19,839,415	1	1.6E-157		rs1362317	16	6,250,379	2	8.3E-05	
rs74841643	6	34,163,292	2	3.4E-37	COL11A2 FANCE	rs4889238	16	81,151,122	3	2.9E-04	
rs9470004	6	35,341,850	1	2.1E-07	FANCE	rs202127176	16	88,782,205	1	5.7E-15	ANKRD11 CDK10 CDT1 FANCA GALNS RPL13
Affx-29864875	7	142,331,582	1	4.7E-04	BRAF						
rs112892337	8	135,614,553	1	8.7E-49		rs58680048	19	51,870,706	1	1.2E-03	

Table 36: Height SNPs with SSV > 1% of total SSV.

SNP	Chromo- -some	GRCh37 position	# of CV folds	Average gwas p-val	Near known assoc. genes	SNP	Chromo- -some	GRCh37 position	# of CV folds	Average gwas p-val	Near known assoc. genes
rs146534110	6	160,578,069	2	0.0E+00	LPA LPAL2 SLC22A3	rs74617384	6	160,997,118	2	0.0E+00	LPA LPAL2 SLC22A3
rs16891156	6	160,608,804	4	0.0E+00	LPA LPAL2 SLC22A3	rs41272114	6	161,006,077	1	3.0E-209	LPA LPAL2 SLC22A3
rs8177505	6	160,679,656	2	0.0E+00	LPA LPAL2 SLC22A3	rs41272112	6	161,006,105	1	1.4E-31	LPA LPAL2 SLC22A3
rs10080815	6	160,687,412	1	0.0E+00	LPA LPAL2 SLC22A3	rs147936725	6	161,007,619	3	1.3E-13	LPA LPAL2 SLC22A3
rs421913	6	160,742,369	1	1.3E-09	LPA LPAL2 SLC22A3	rs10455872	6	161,010,118	5	0.0E+00	LPA LPAL2 SLC22A3
rs540713	6	160,766,321	3	2.2E-117	LPA LPAL2 SLC22A3	rs41272078	6	161,010,150	2	1.3E-89	LPA LPAL2 SLC22A3
rs3918291	6	160,828,142	2	0.0E+00	LPA LPAL2 SLC22A3	rs140306630	6	161,013,826	3	6.4E-09	LPA LPAL2 SLC22A3
rs12214416	6	160,910,517	1	0.0E+00	LPA LPAL2 SLC22A3	rs73596816	6	161,017,363	5	0.0E+00	LPA LPAL2 SLC22A3
rs72501790	6	160,915,856	3	1.6E-18	LPA LPAL2 SLC22A3	rs41259144	6	161,022,107	2	6.9E-62	LPA LPAL2 SLC22A3
rs41267807	6	160,952,816	1	3.0E-149	LPA LPAL2 SLC22A3	rs143079629	6	161,128,812	1	1.1E-33	LPA LPAL2 SLC22A3
rs3798220	6	160,961,137	5	0.0E+00	LPA LPAL2 SLC22A3	rs4252128	6	161,152,819	3	1.6E-11	LPA LPAL2 SLC22A3
rs116089584	6	160,962,370	1	6.7E-65	LPA LPAL2 SLC22A3	rs4252152	6	161,159,366	5	0.0E+00	LPA LPAL2 SLC22A3
rs7767084	6	160,962,503	1	1.8E-76	LPA LPAL2 SLC22A3						

Table 37: Lipoprotein A SNPs with SSV > 1% of total SSV.

7 Predictor variance

The main text details various ways to analyze the variance described by sparse PGS: how it relates to phenotypic variance explained, the fraction of variance described by single SNP variance (SSV), and the distribution of SSV as a function of training size and cross-validation fold. Here we compile the results for the phenotypes not presented in the main text. **Figure 17-Figure 22** demonstrate the fraction of phenotypic variance explained by PGS variance. **Figure 23-Figure 28** compare the total PGS variance to the SSV. **Figure 31-Figure 56** demonstrate the SSV, and beta size and distribution, as a function of training size and cross-validation fold. In **Figure 57-Figure 62** we show the sparsity as a function of SSV cut, i.e. if we sort SNPs by SSV and then count the number that account for a particular fraction of SSV. Finally in **Figure 64-Figure 68** we show how SSV is demonstrated throughout the autosome.

7.1 Fraction of variance explained

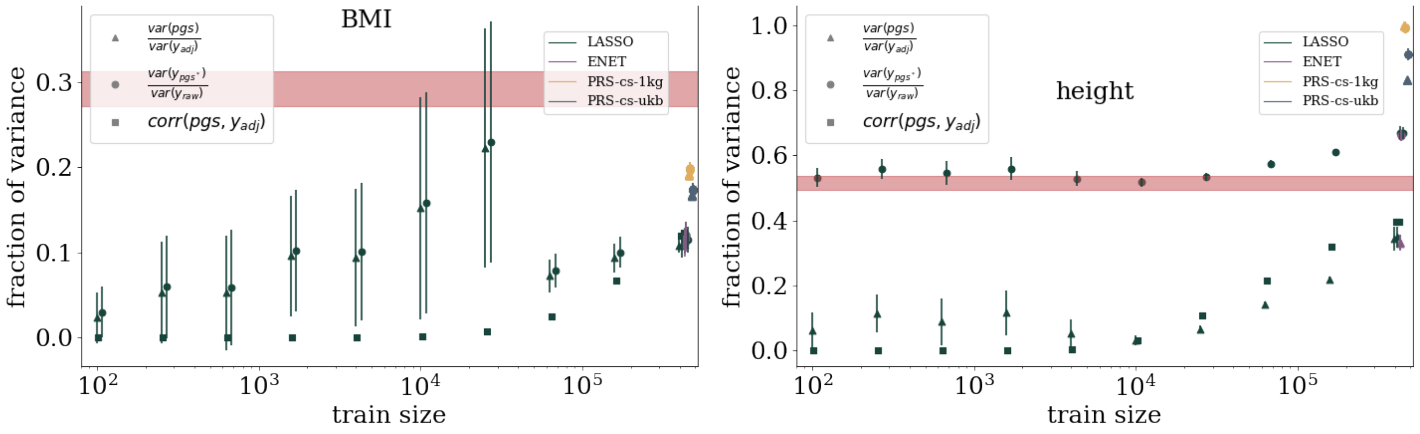

Figure 17: Fraction of variance explained estimates for BMI and height. Red band is the result from using GCTA.

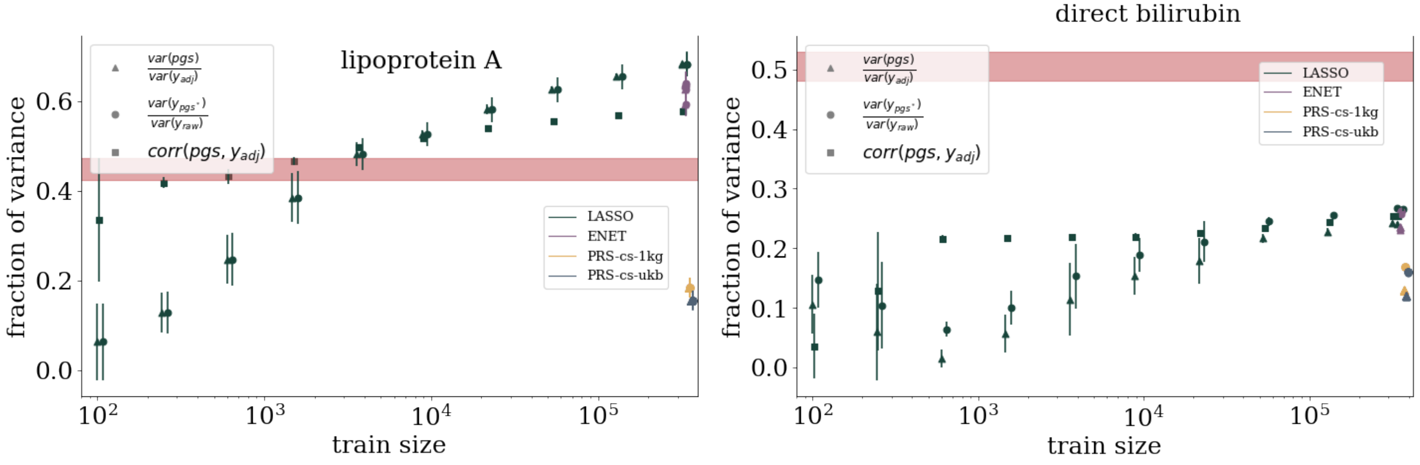

Figure 18: Fraction of variance explained estimates for lipoprotein A and direct bilirubin. Red band is the result from using GCTA.

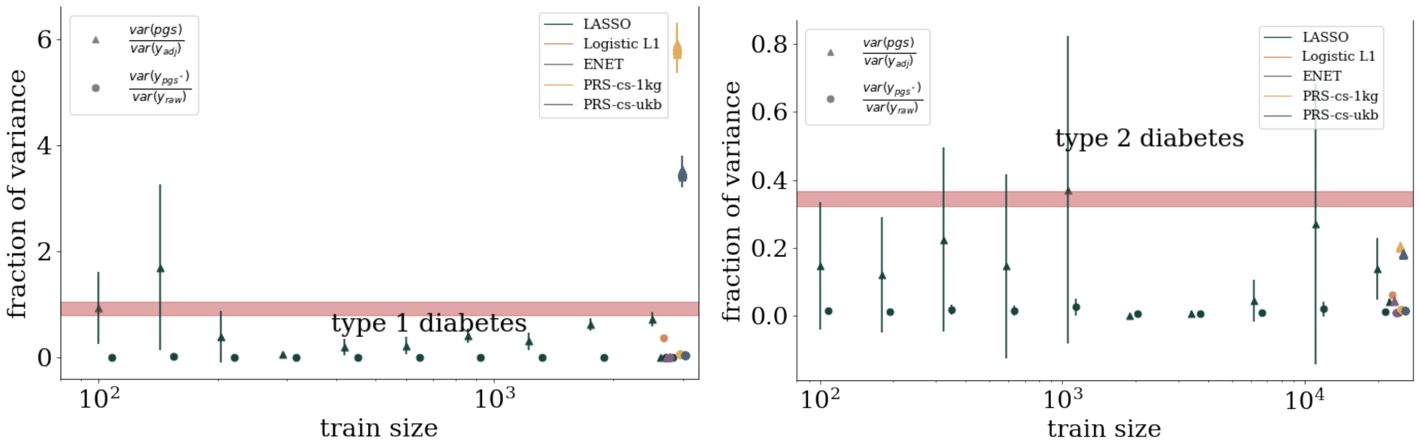

Figure 19: Fraction of variance explained estimates for diabetes. Red band is the result from using GCTA.

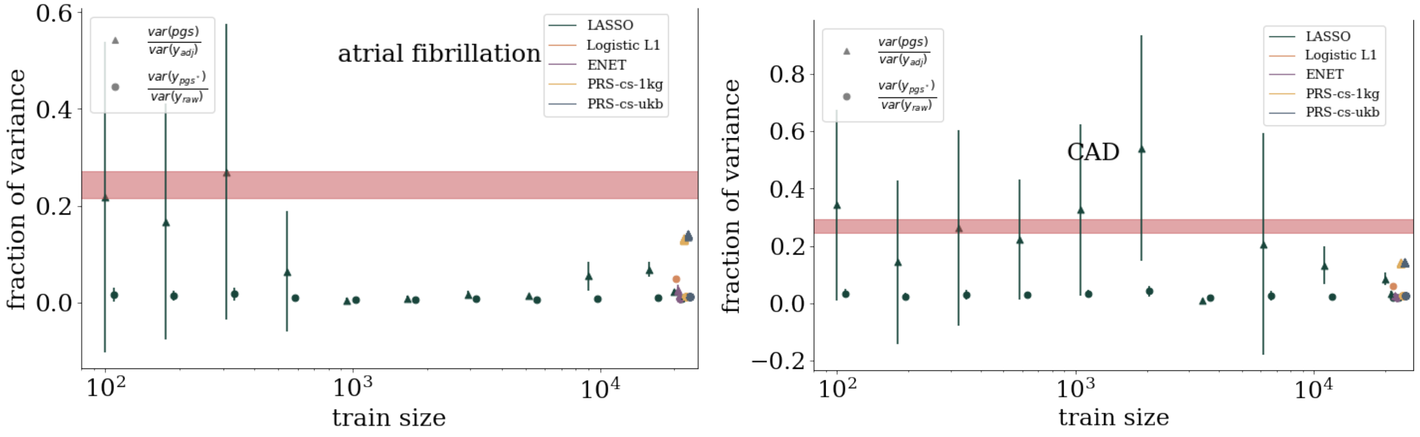

Figure 20: Fraction of variance explained estimates for atrial fibrillation and CAD. Red band is the result from using GCTA.

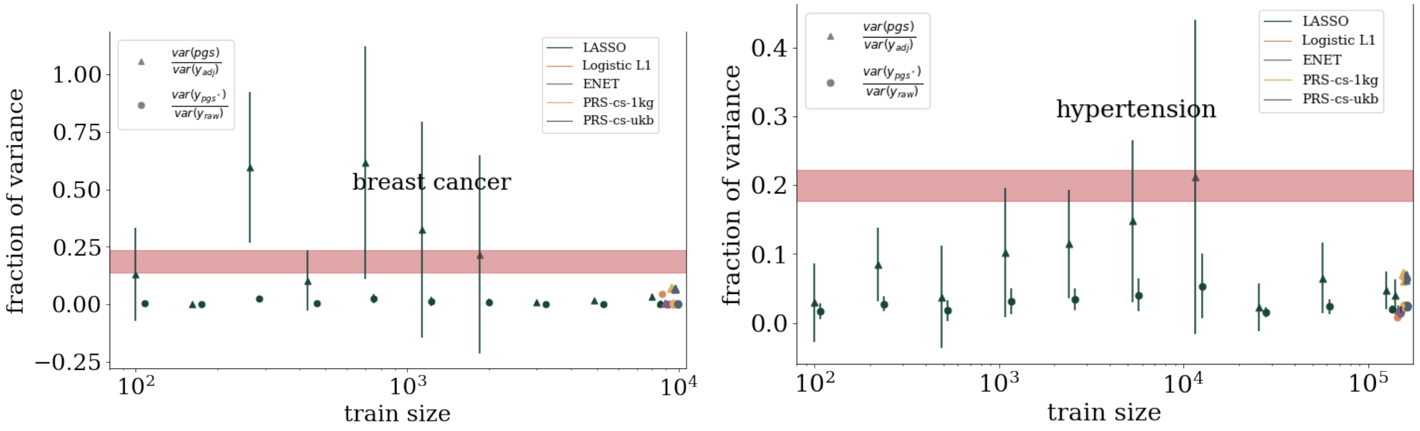

Figure 21: Fraction of variance explained estimates for breast cancer and hypertension. Red band is the result from using GCTA.

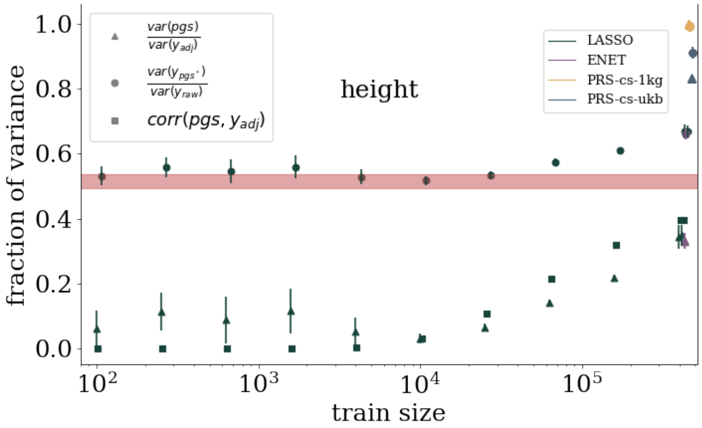

Figure 22: Fraction of variance explained estimates for height. Red band is the result from using GCTA.

7.2 SSV vs total variance

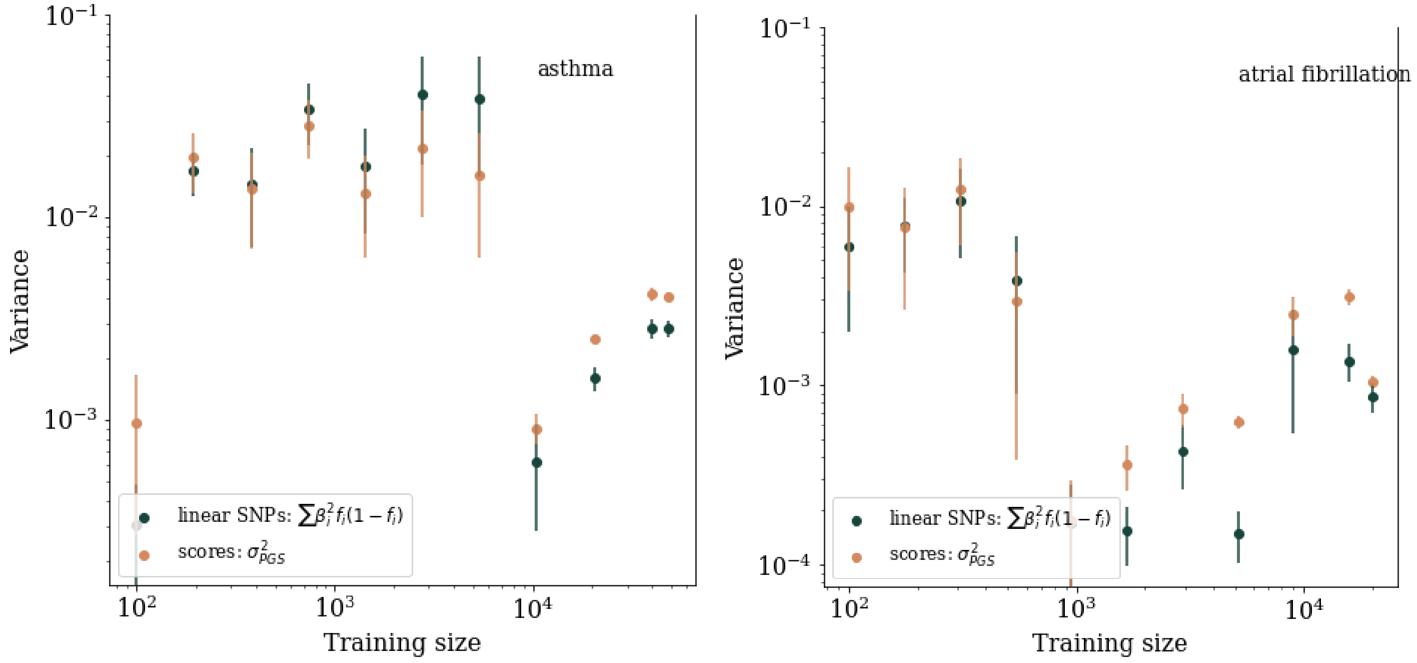

Figure 23: Comparison of SSV to total LASSO predictor variance for asthma and atrial fibrillation.

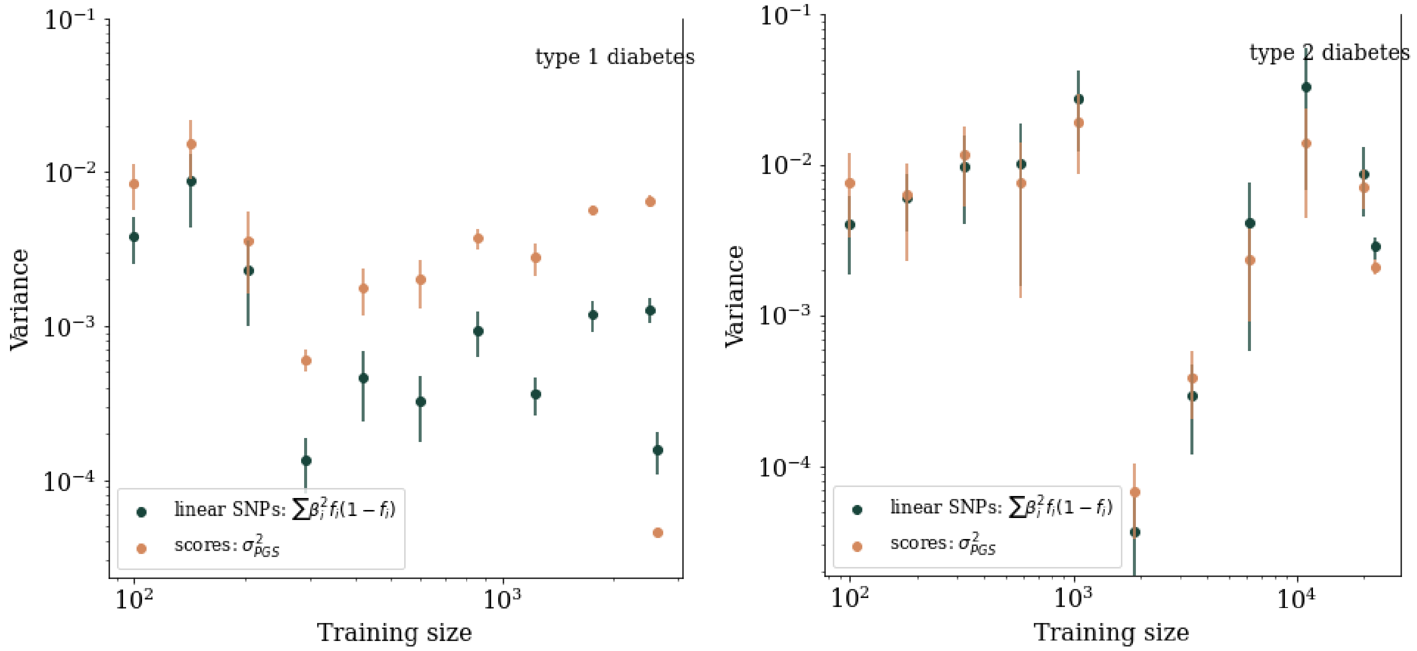

Figure 24: Comparison of SSV to total LASSO predictor variance for type 1 and 2 diabetes.

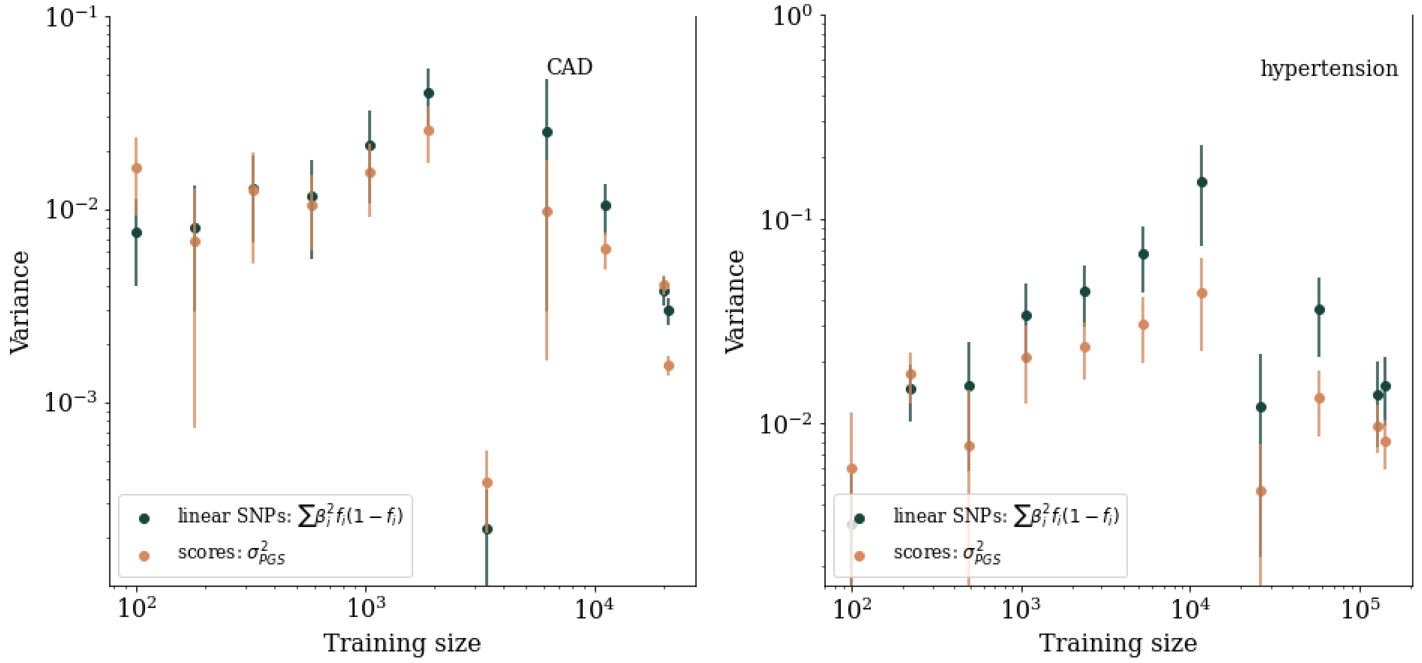

Figure 25: Comparison of SSV to total LASSO predictor variance for CAD and hypertension.

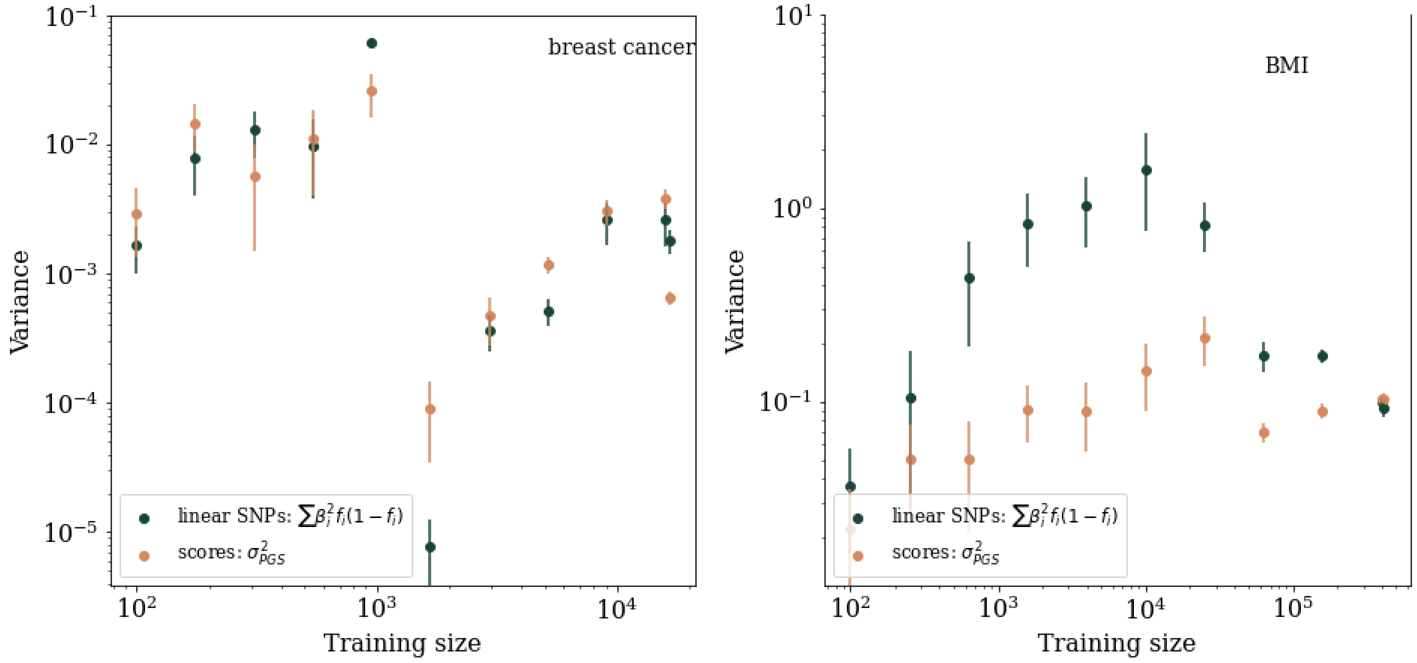

Figure 26: Comparison of SSV to total LASSO predictor variance for breast cancer and BMI.

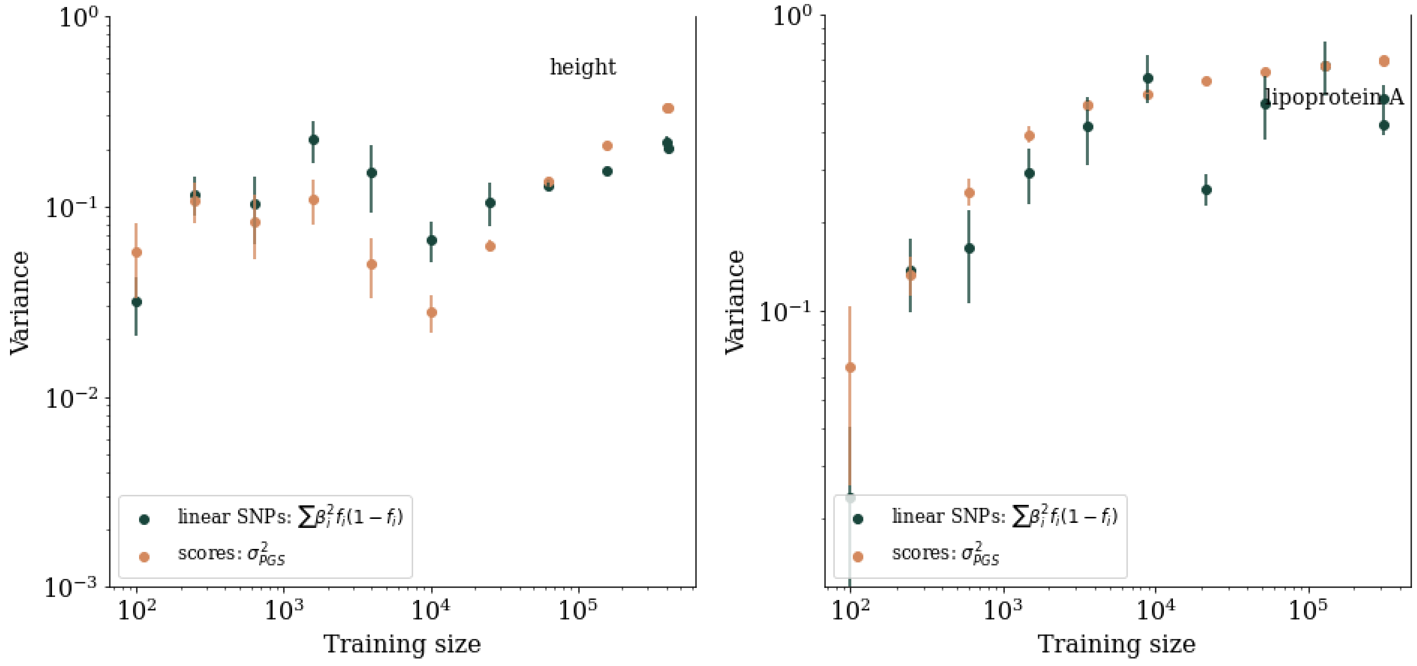

Figure 27: Comparison of SSV to total LASSO predictor variance for height and lipoprotein A.

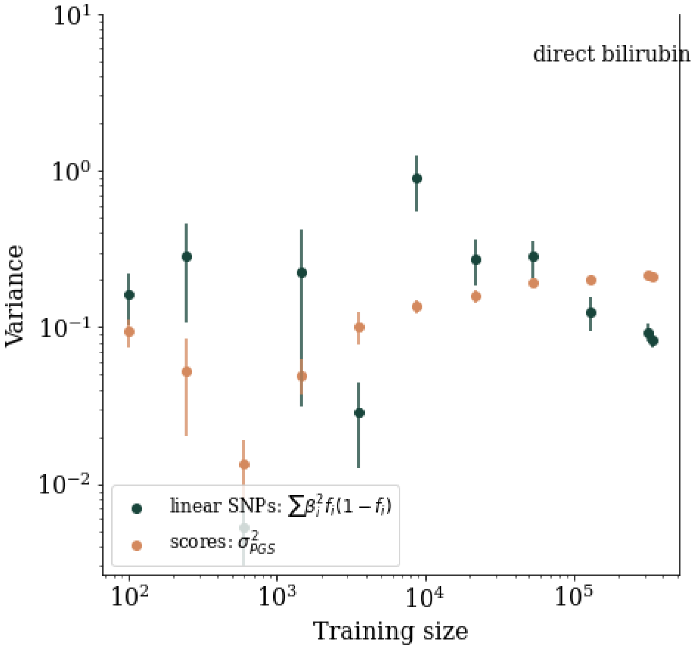

Figure 28: Comparison of SSV to total LASSO predictor variance for direct bilirubin.

7.3 Predictor impact regions

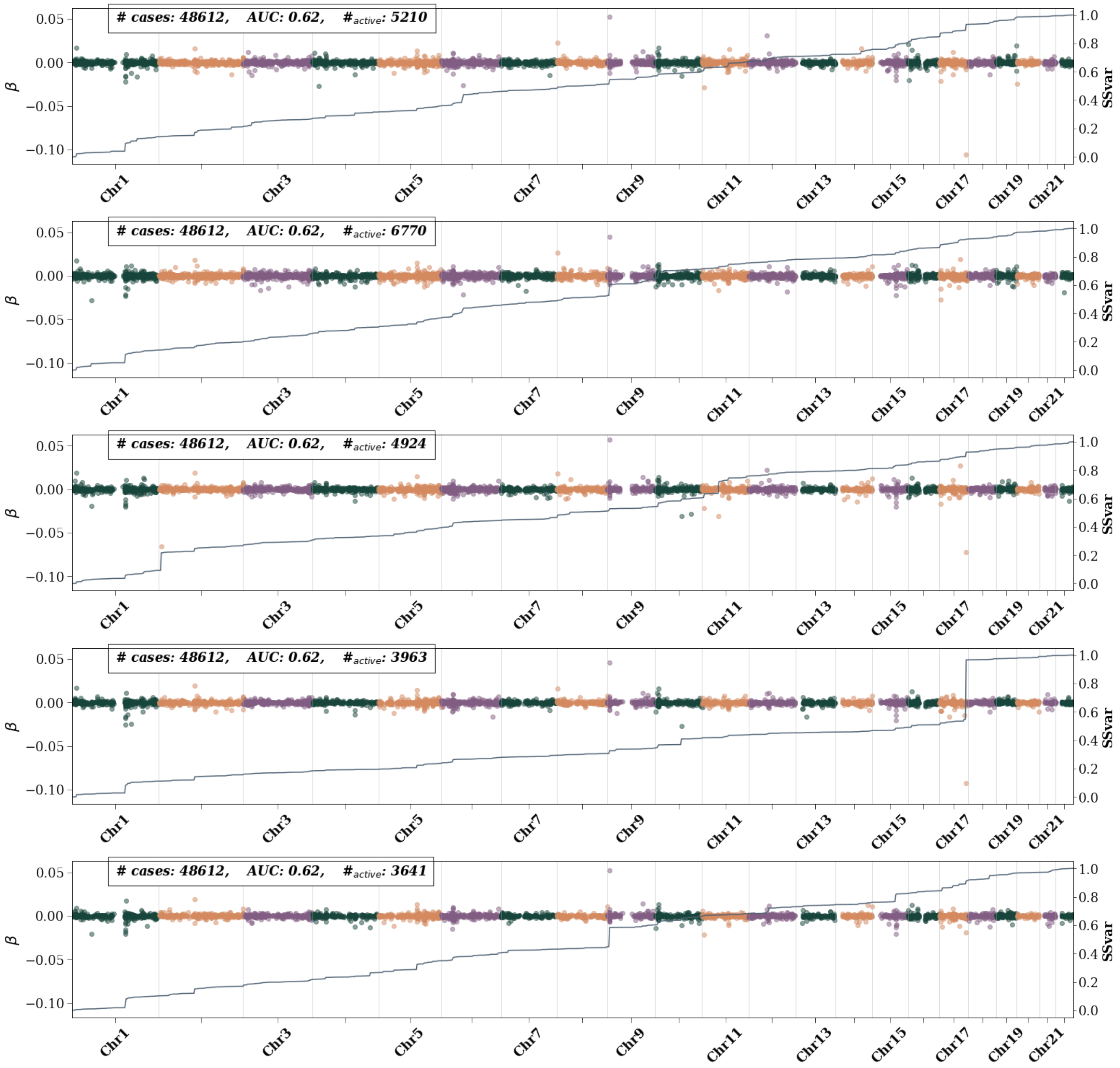

Figure 29: Asthma active SNPs – i.e., SNPs with non-zero β weights– for 5 CV folds at maximum training size. Left axis shows the β value and is represented by colored dots. Different colors are used to differentiate chromosomes. The right axis represents the single SNP variance (SSV) normalized to the total SSV. The “training” label represents the number of cases used in training. All possible controls were used in each fold. While features generally appear consistent across folds, i.e., the presence of a bump in the SSV line, the size of the bump varies.

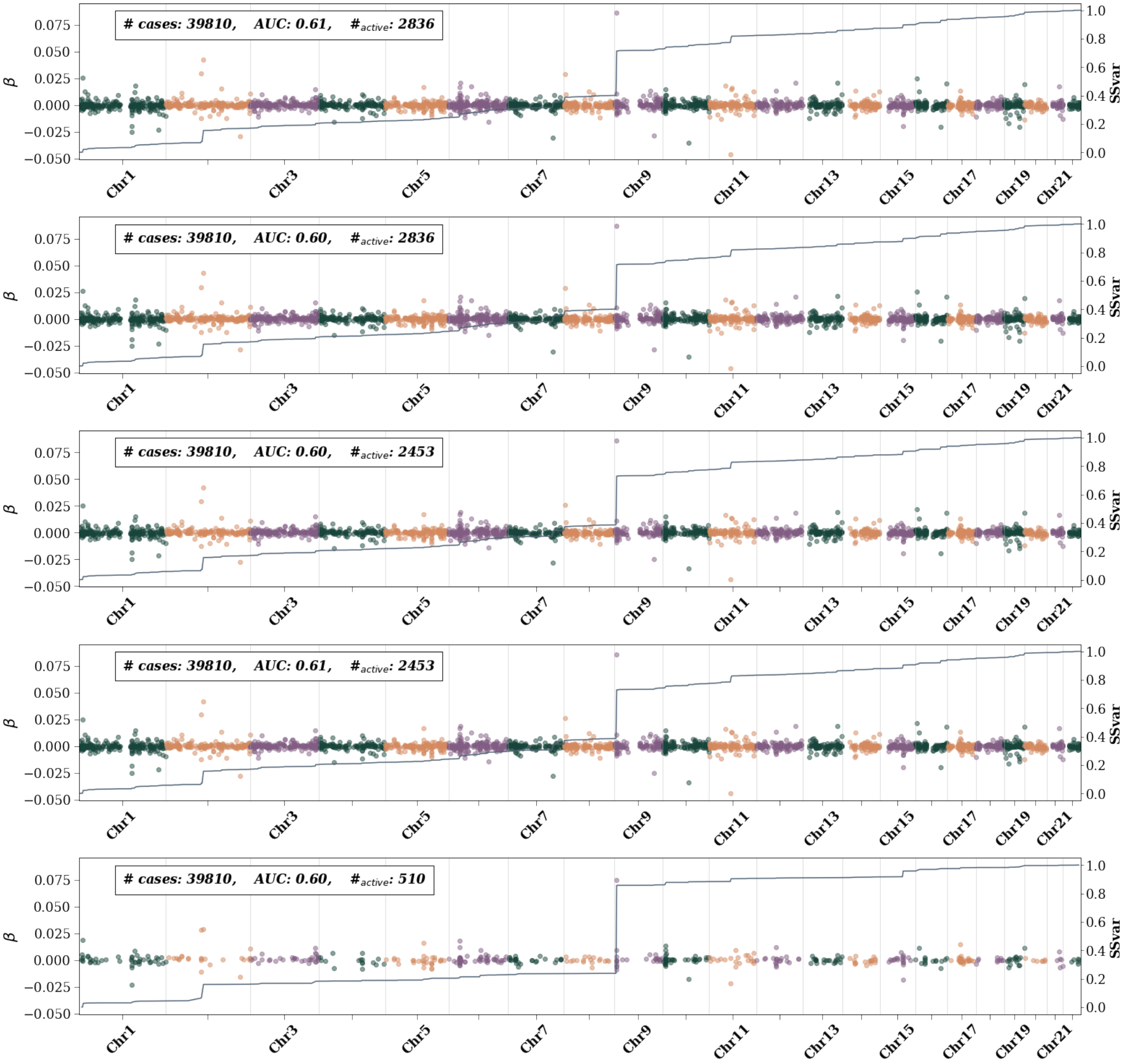

Figure 30: Asthma active SNPs – i.e., SNPs with non-zero β weights– for 5 CV folds at near-maximum training size, but with equal cases and controls. Left axis shows the β value and is represented by colored dots. Different colors are used to differentiate chromosomes. The right axis represents the single SNP variance (SSV) normalized to the total SSV. The "training" label represents the number of cases used in training (an equal number of controls also used). Compared to maximal training in **Figure 29**, the features here are much more consistent across folds.

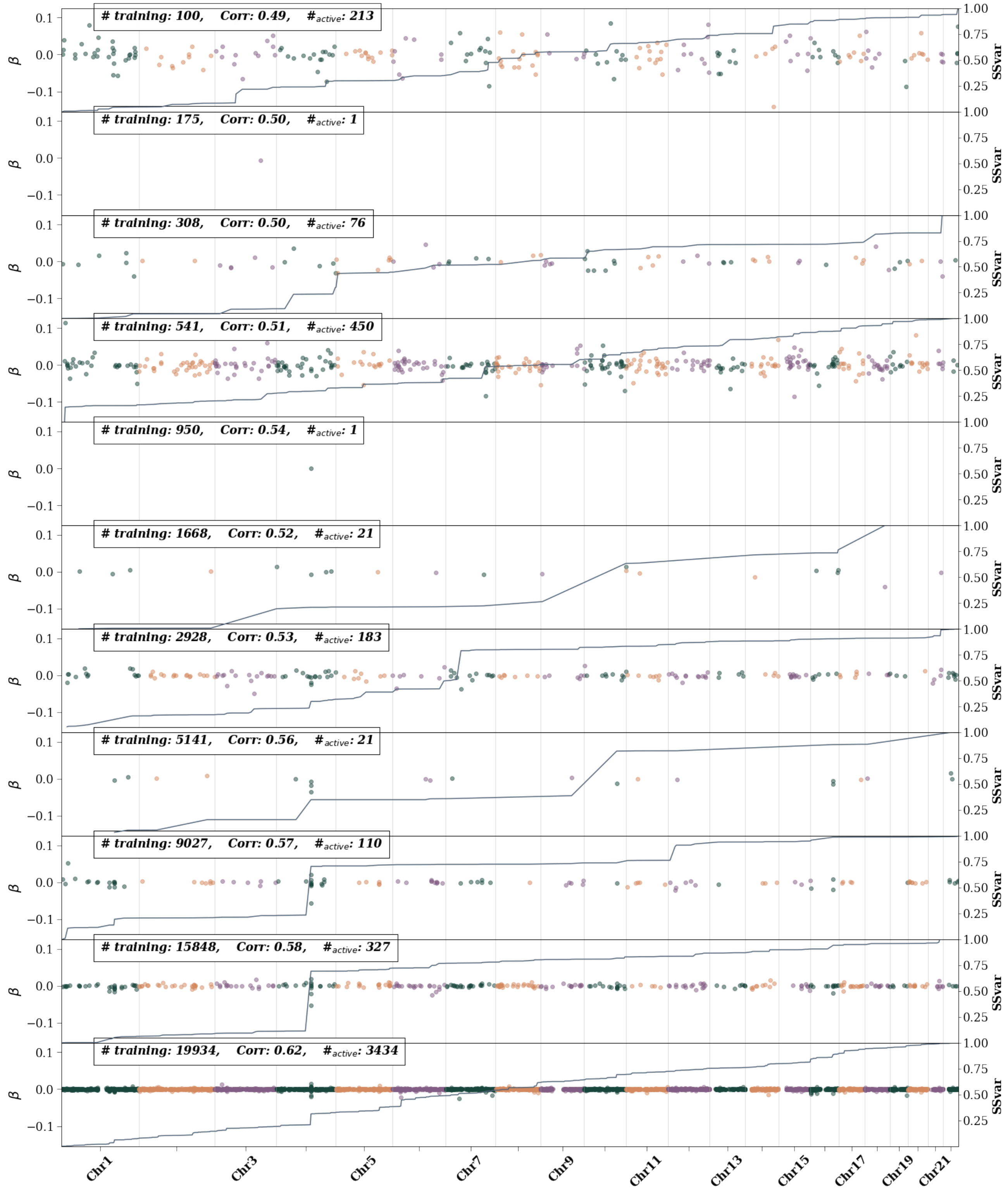

Figure 31: Atrial fibrillation active SNPs – i.e., SNPs with non-zero β weights– as training size is increased. The left axis shows the β value and is represented by colored dots. Different colors are used to differentiate chromosomes. The right axis represents the single SNP variance (SSV) normalized to the total SSV. The solid line showes the cumulative SSV. The "training" label represents the number of cases used in training. The first 10 (from the top) training sizes use equal number of cases and controls. The final training size uses all possible remaining controls.

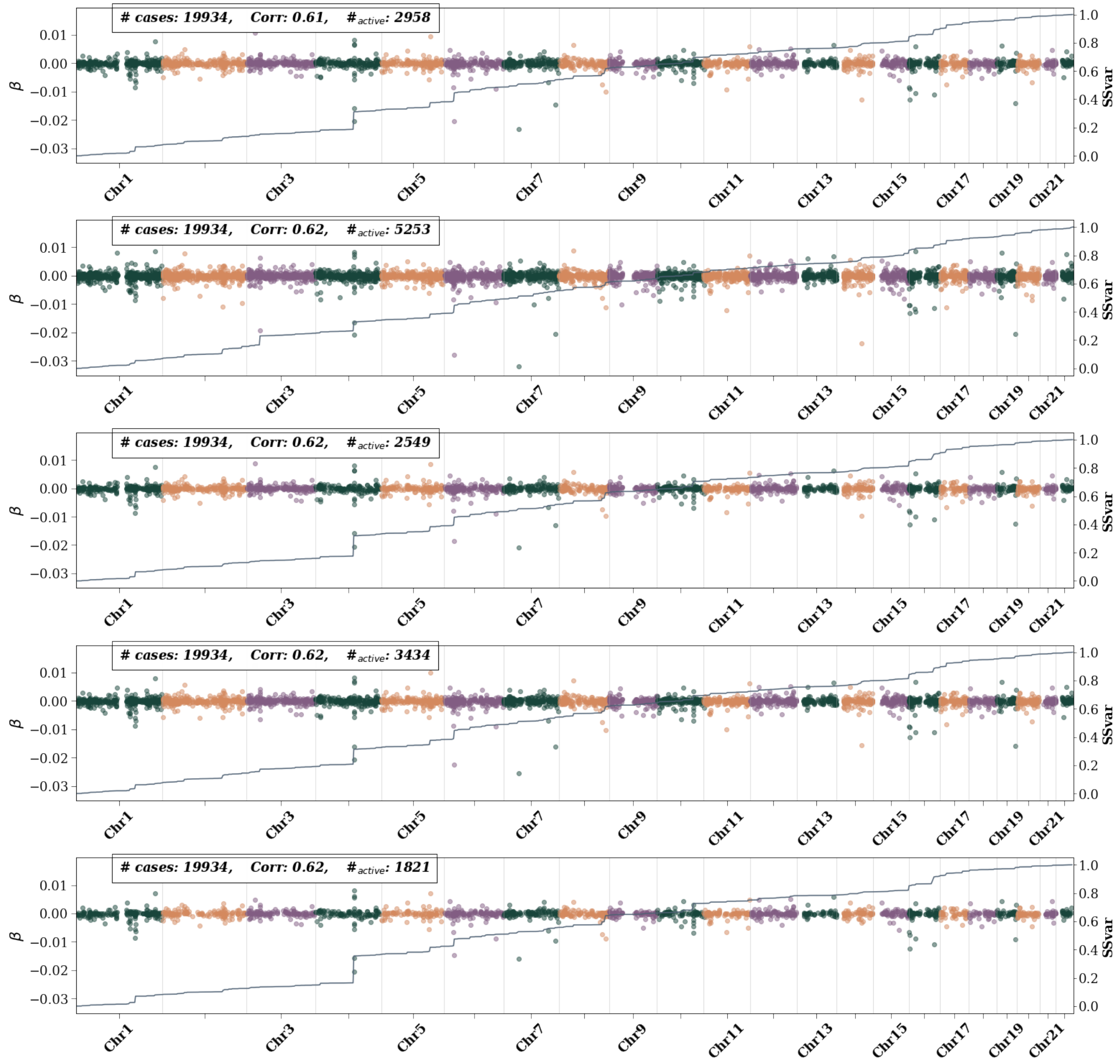

Figure 32: Atrial fibrillation active SNPs – i.e., SNPs with non-zero β weights– for 5 CV folds at maximum training size. Left axis shows the β value and is represented by colored dots. Different colors are used to differentiate chromosomes. The right axis represents the single SNP variance (SSV) normalized to the total SSV. The "training" label represents the number of cases used in training. All possible controls were used in each fold. While features generally appear consistent across folds, i.e., the presence of a bump in the SSV line, the size of the bump varies.

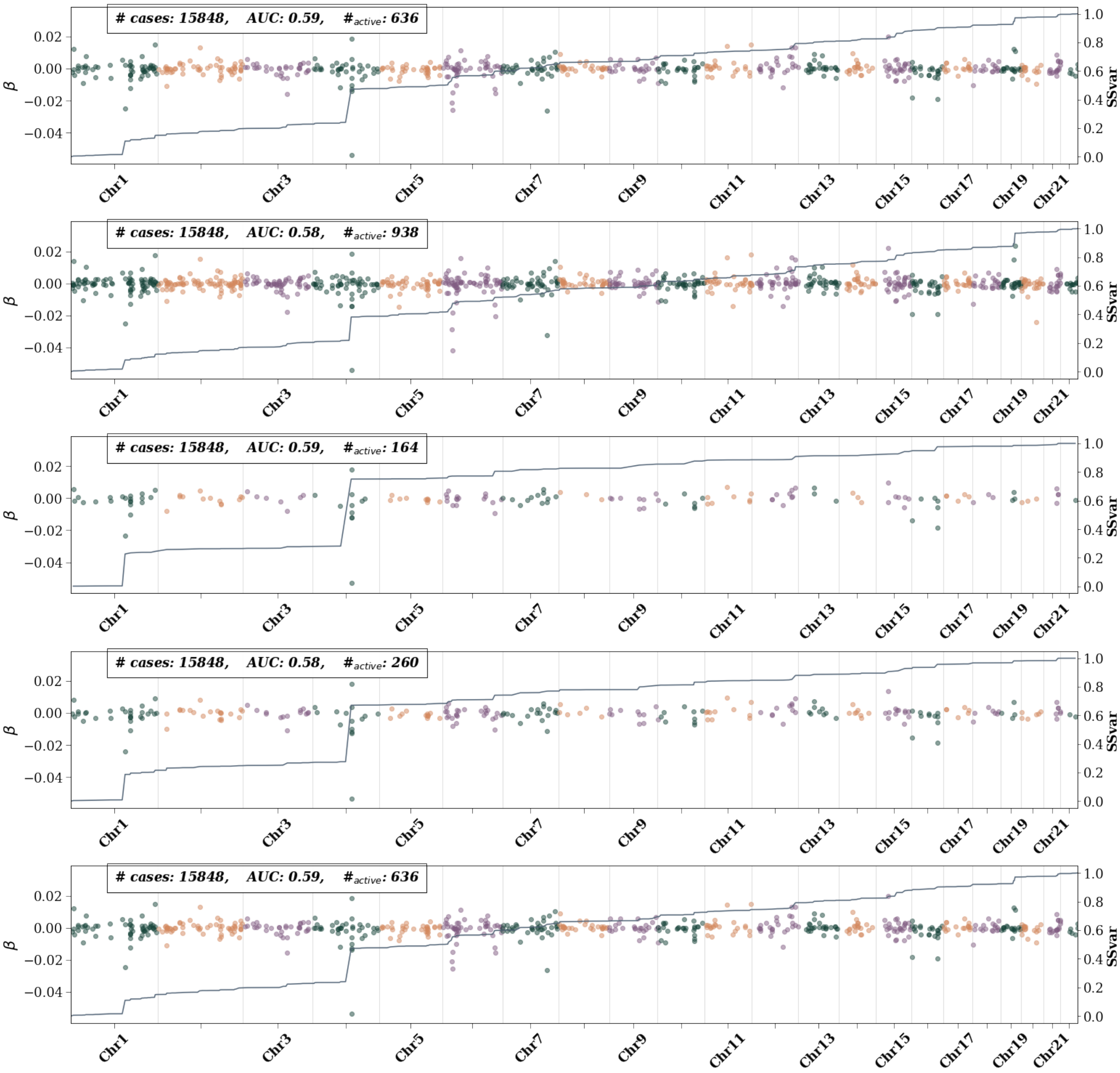

Figure 33: Atrial fibrillation active SNPs – i.e., SNPs with non-zero β weights– for 5 CV folds at near-maximum training size, but with equal cases and controls. Left axis shows the β value and is represented by colored dots. Different colors are used to differentiate chromosomes. The right axis represents the single SNP variance (SSV) normalized to the total SSV. The "training" label represents the number of cases used in training (an equal number of controls also used). Compared to maximal training in **Figure 32**, the features here are much more consistent across folds.

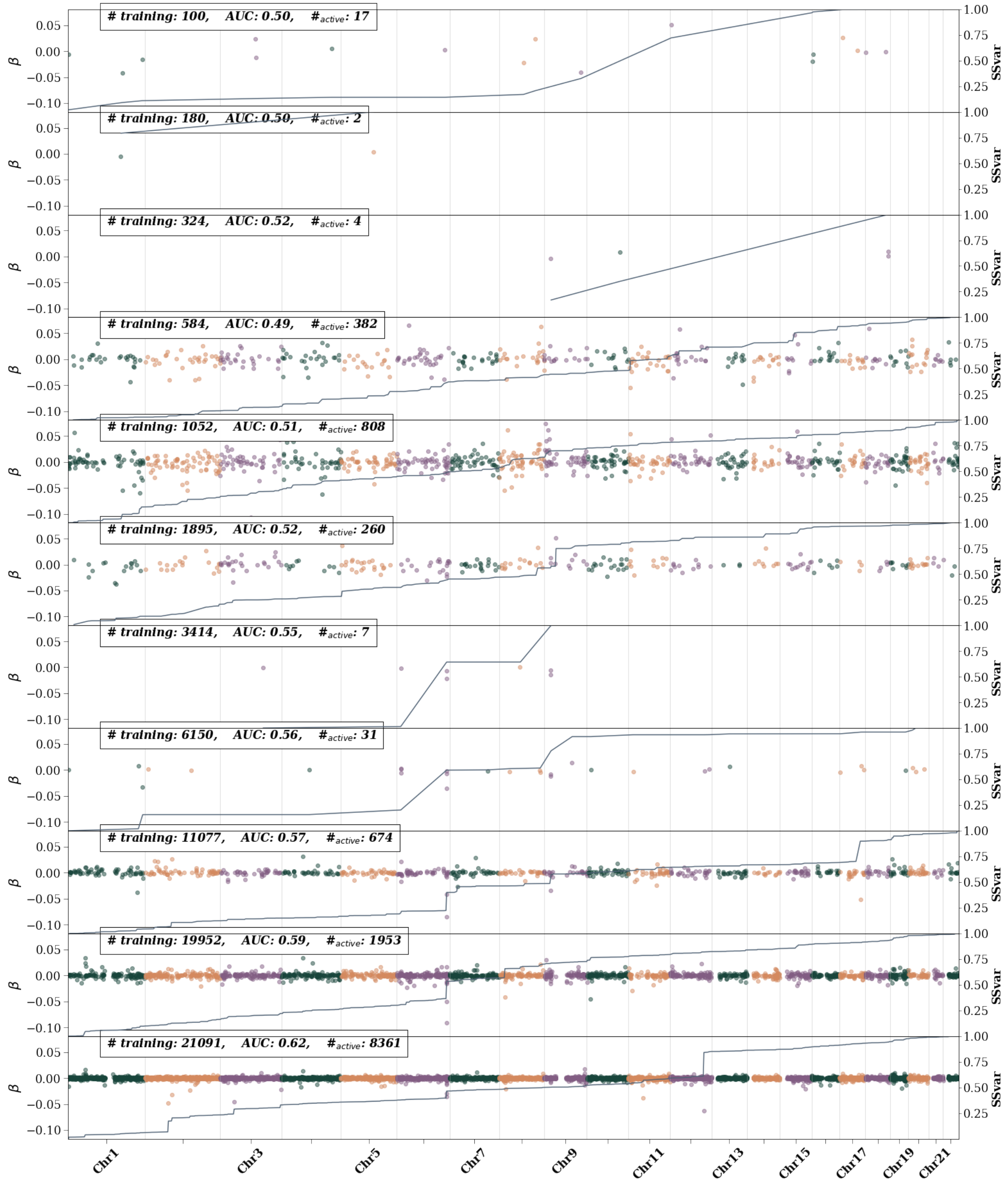

Figure 34: Coronary artery disease active SNPs – i.e., SNPs with non-zero β weights– as training size is increased. The left axis shows the β value and is represented by colored dots. Different colors are used to differentiate chromosomes. The right axis represents the single SNP variance (SSV) normalized to the total SSV. The solid line showes the cumulative SSV. The "training" label represents the number of cases used in training. The first 10 (from the top) training sizes use equal number of cases and controls. The final training size uses all possible remaining controls

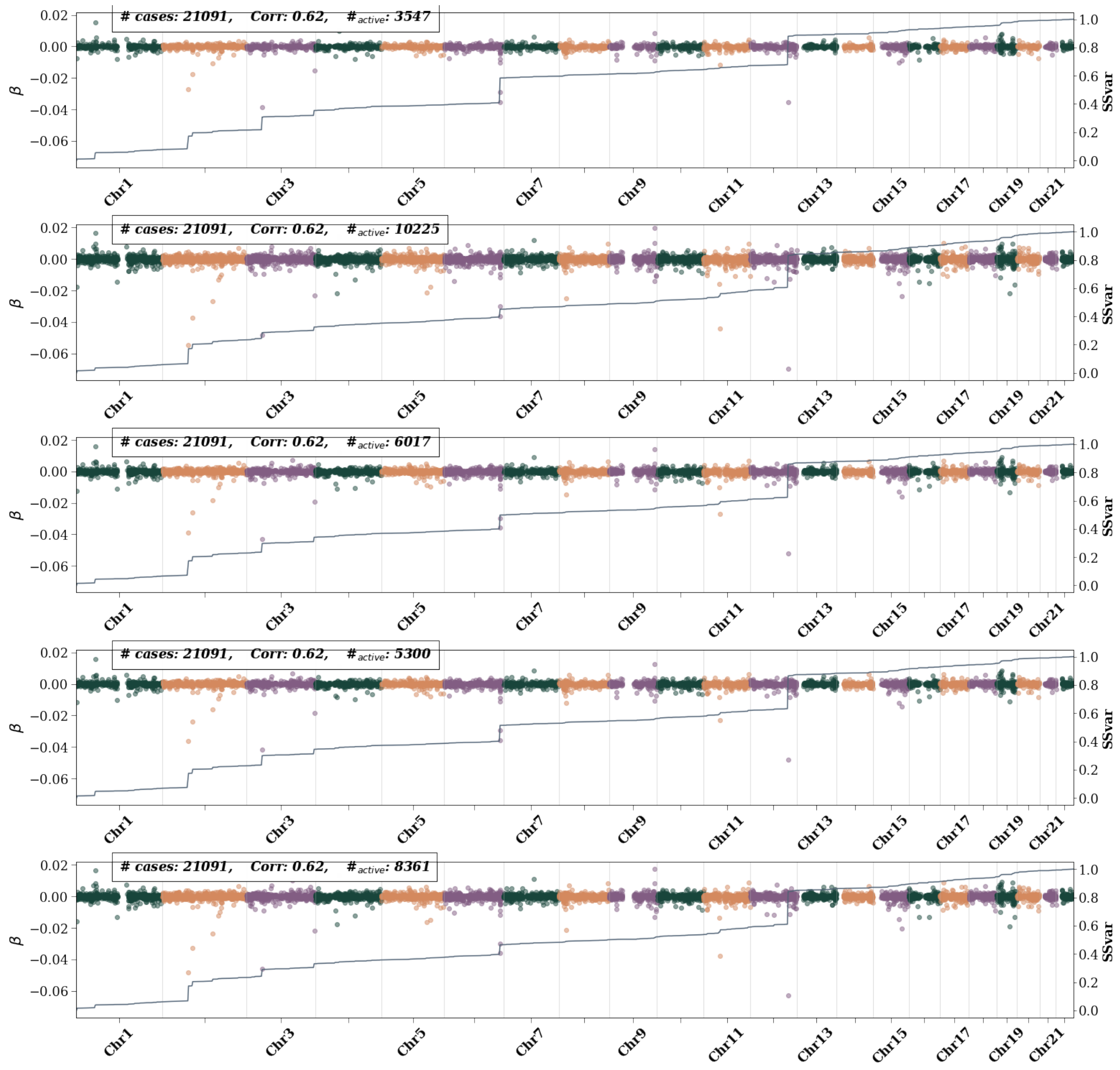

Figure 35: CAD active SNPs – i.e., SNPs with non-zero β weights– for 5 CV folds at maximum training size. Left axis shows the β value and is represented by colored dots. Different colors are used to differentiate chromosomes. The right axis represents the single SNP variance (SSV) normalized to the total SSV. The "training" label represents the number of cases used in training. All possible controls were used in each fold. While features generally appear consistent across folds, i.e., the presence of a bump in the SSV line, the size of the bump varies.

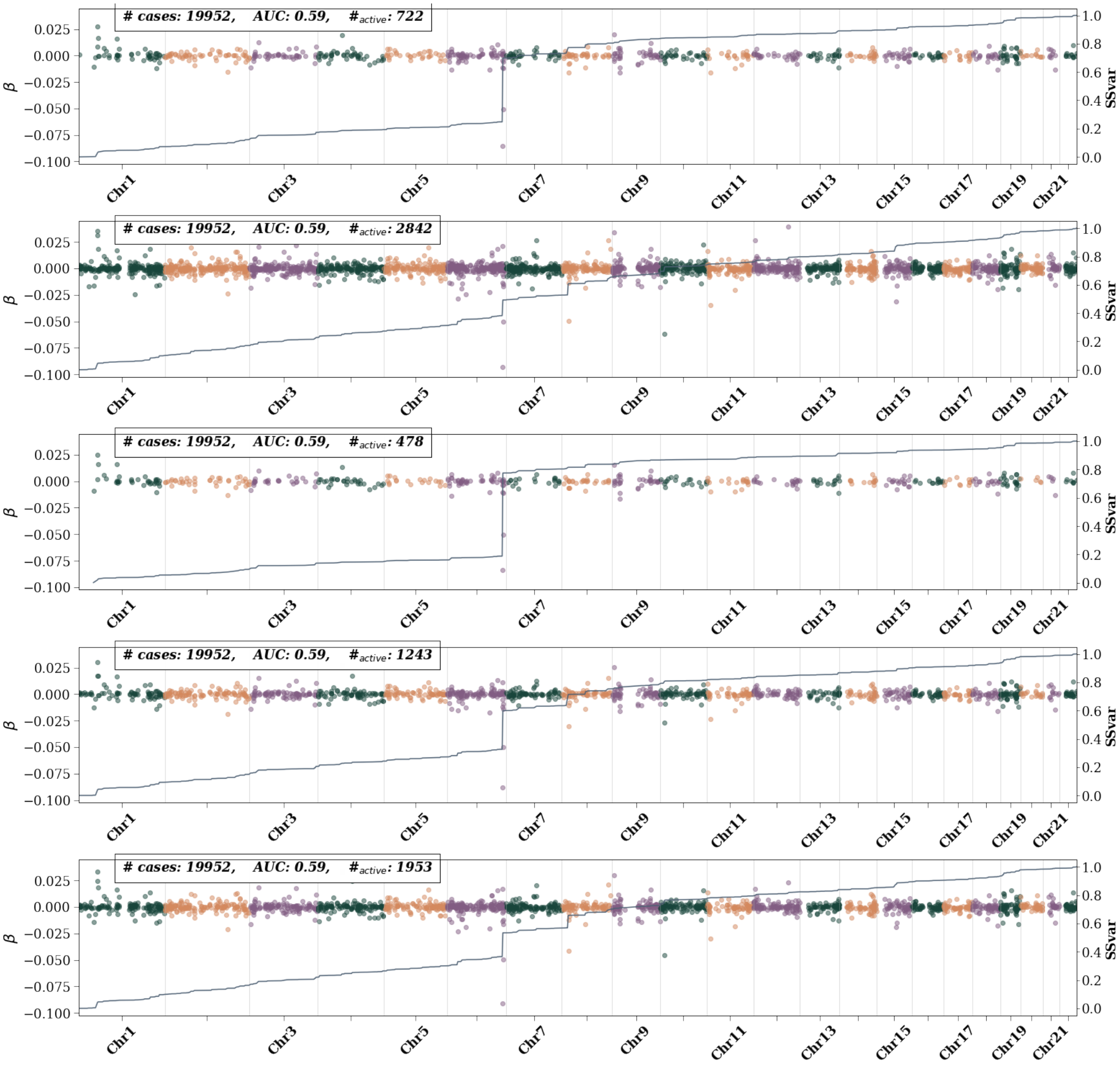

Figure 36: Atrial fibrillation active SNPs – i.e., SNPs with non-zero β weights– for 5 CV folds at near-maximum training size, but with equal cases and controls. Left axis shows the β value and is represented by colored dots. Different colors are used to differentiate chromosomes. The right axis represents the single SNP variance (SSV) normalized to the total SSV. The "training" label represents the number of cases used in training (an equal number of controls also used). Compared to maximal training in **Figure 35**, the features here are much more consistent across folds.

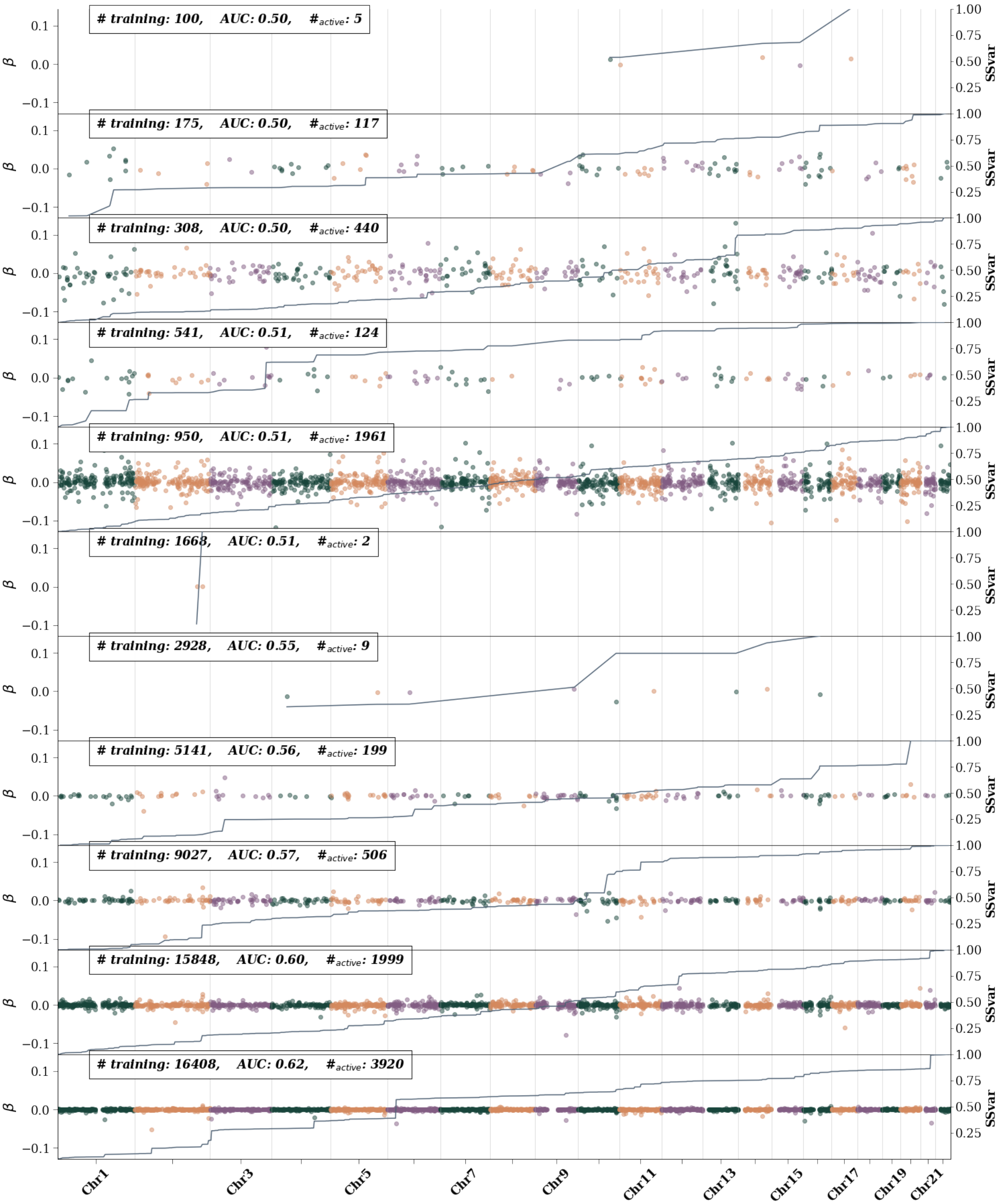

Figure 37: Breast cancer active SNPs – i.e., SNPs with non-zero β weights– as training size is increased. The left axis shows the β value and is represented by colored dots. Different colors are used to differentiate chromosomes. The right axis represents the single SNP variance (SSV) normalized to the total SSV. The solid line showes the cumulative SSV. The "training" label represents the number of cases used in training. The first 10 (from the top) training sizes use equal number of cases and controls. The final training size uses all possible remaining controls

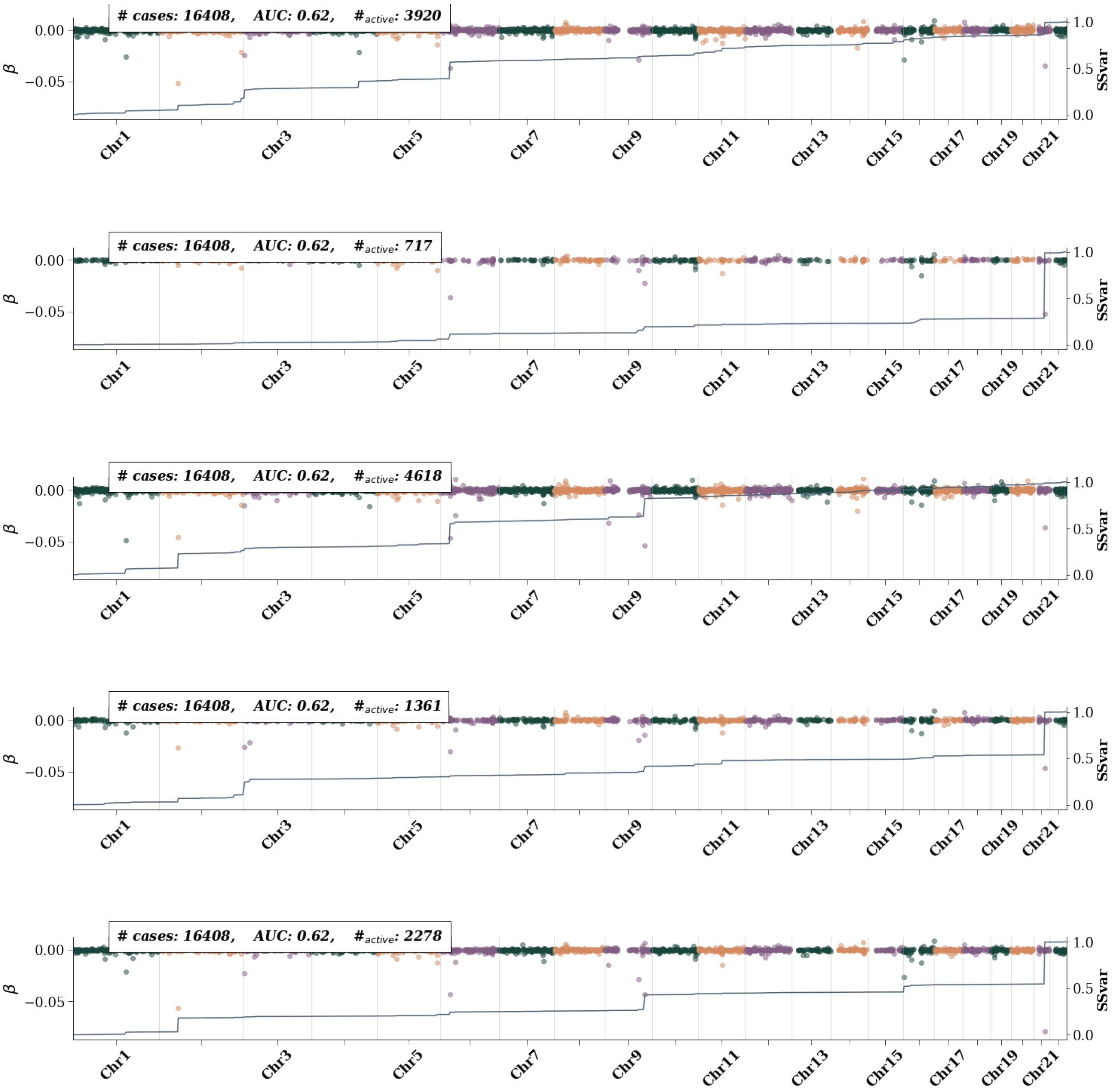

Figure 38: Breast cancer active SNPs – i.e., SNPs with non-zero β weights– for 5 CV folds at maximum training size. Left axis shows the β value and is represented by colored dots. Different colors are used to differentiate chromosomes. The right axis represents the single SNP variance (SSV) normalized to the total SSV. The "training" label represents the number of cases used in training. All possible controls were used in each fold. While features generally appear consistent across folds, i.e., the presence of a bump in the SSV line, the size of the bump varies.

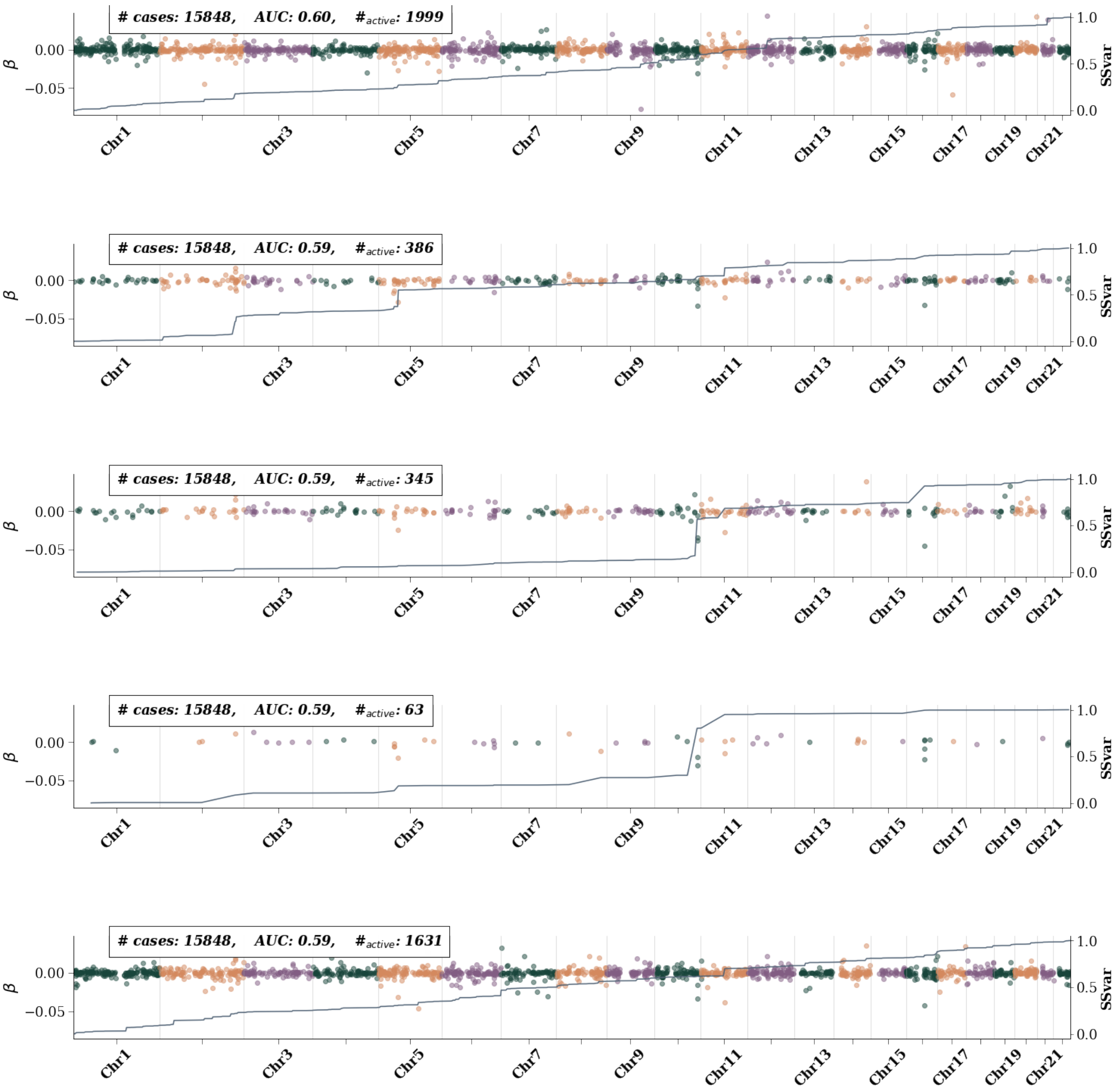

Figure 39: Atrial fibrillation active SNPs – i.e., SNPs with non-zero β weights– for 5 CV folds at near-maximum training size, but with equal cases and controls. Left axis shows the β value and is represented by colored dots. Different colors are used to differentiate chromosomes. The right axis represents the single SNP variance (SSV) normalized to the total SSV. The "training" label represents the number of cases used in training (an equal number of controls also used). Compared to maximal training in **Figure 38**, the features here are much more consistent across folds.

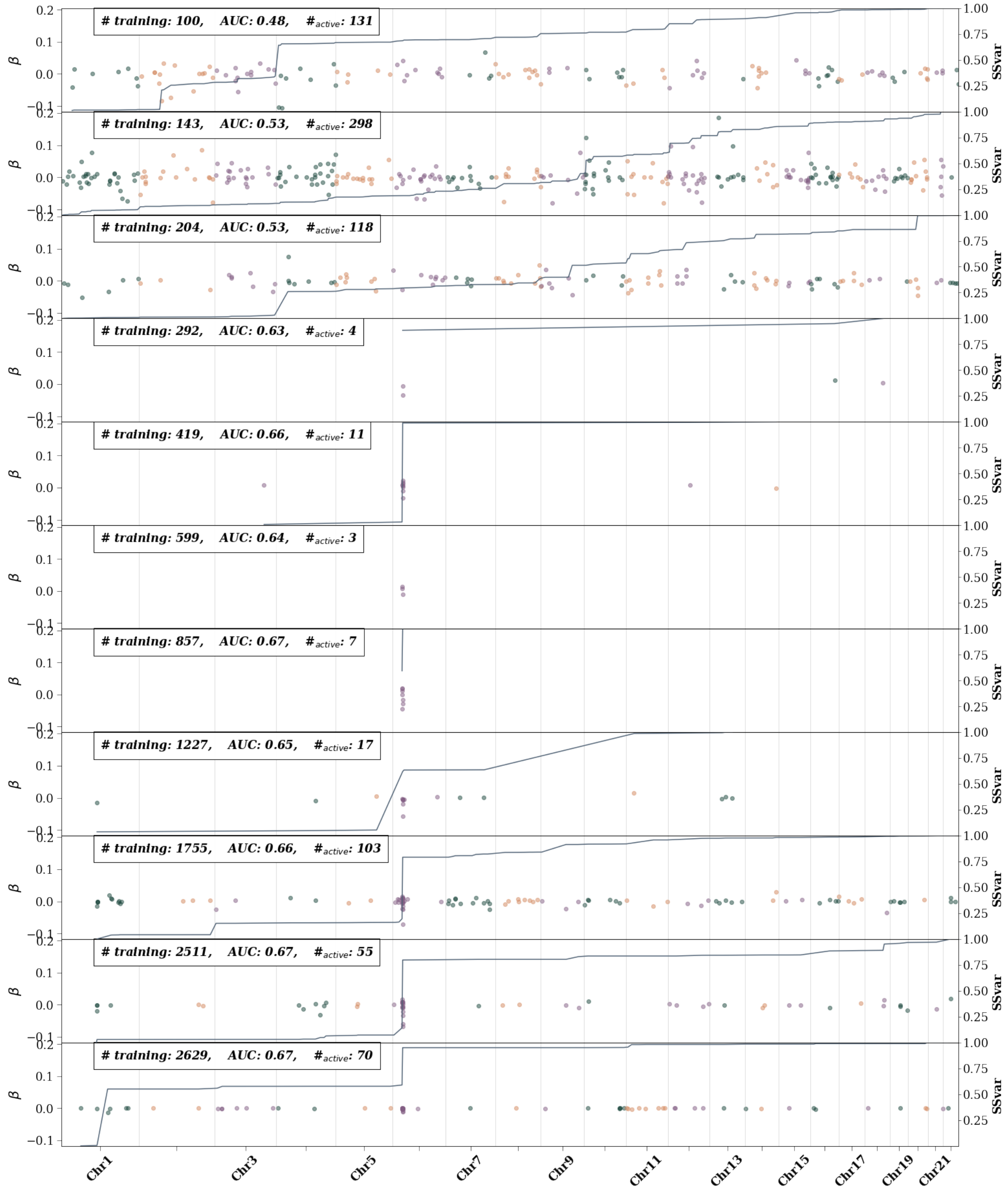

Figure 40: Type 1 diabetes active SNPs – i.e., SNPs with non-zero β weights– as training size is increased. The left axis shows the β value and is represented by colored dots. Different colors are used to differentiate chromosomes. The right axis represents the single SNP variance (SSV) normalized to the total SSV. The solid line showes the cumulative SSV. The "training" label represents the number of cases used in training. The first 10 (from the top) training sizes use equal number of cases and controls. The final training size uses all possible remaining controls

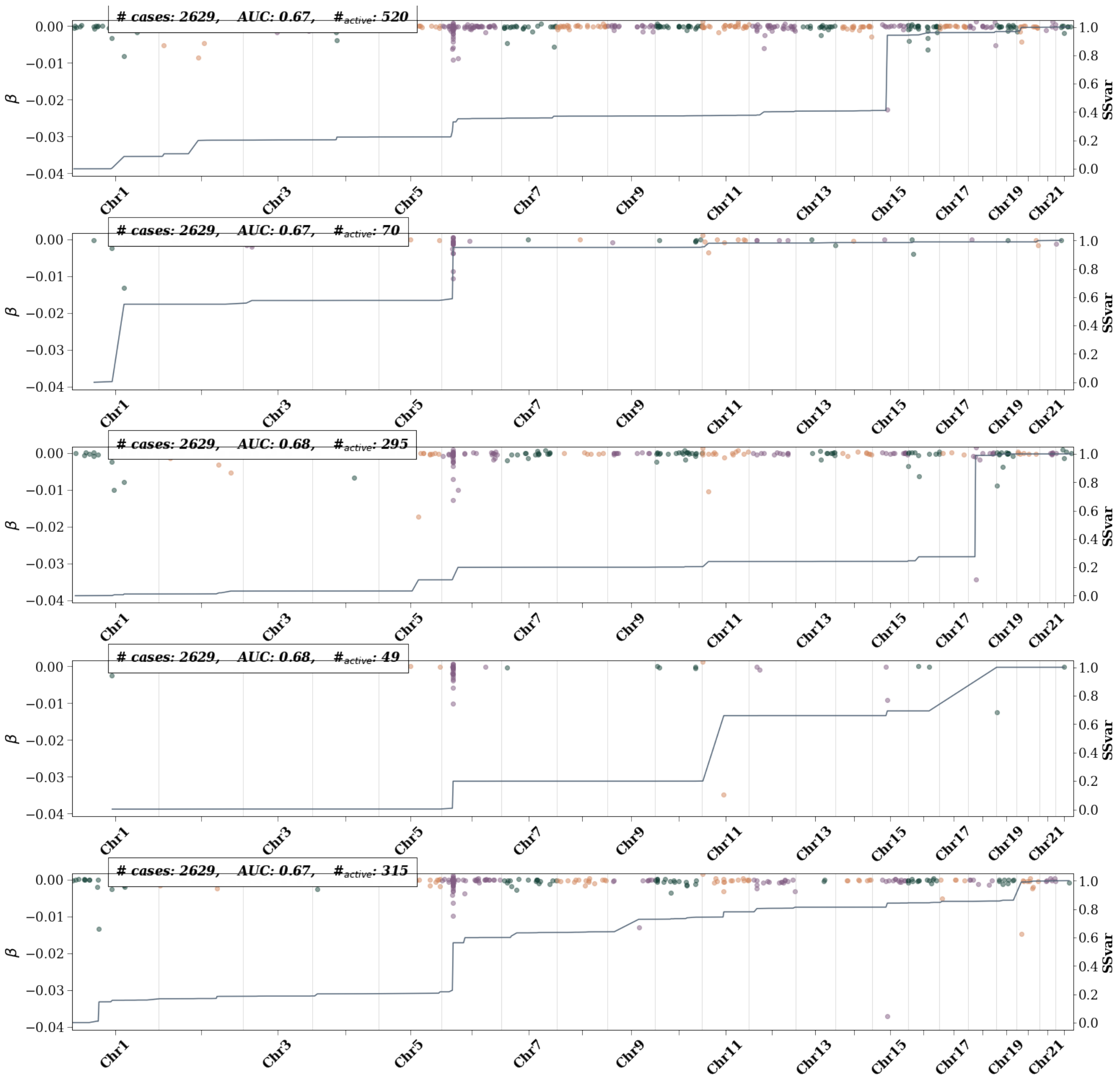

Figure 41: Type 1 diabetes active SNPs – i.e., SNPs with non-zero β weights– for 5 CV folds at maximum training size. Left axis shows the β value and is represented by colored dots. Different colors are used to differentiate chromosomes. The right axis represents the single SNP variance (SSV) normalized to the total SSV. The "training" label represents the number of cases used in training. All possible controls were used in each fold. While features generally appear consistent across folds, i.e., the presence of a bump in the SSV line, the size of the bump varies.

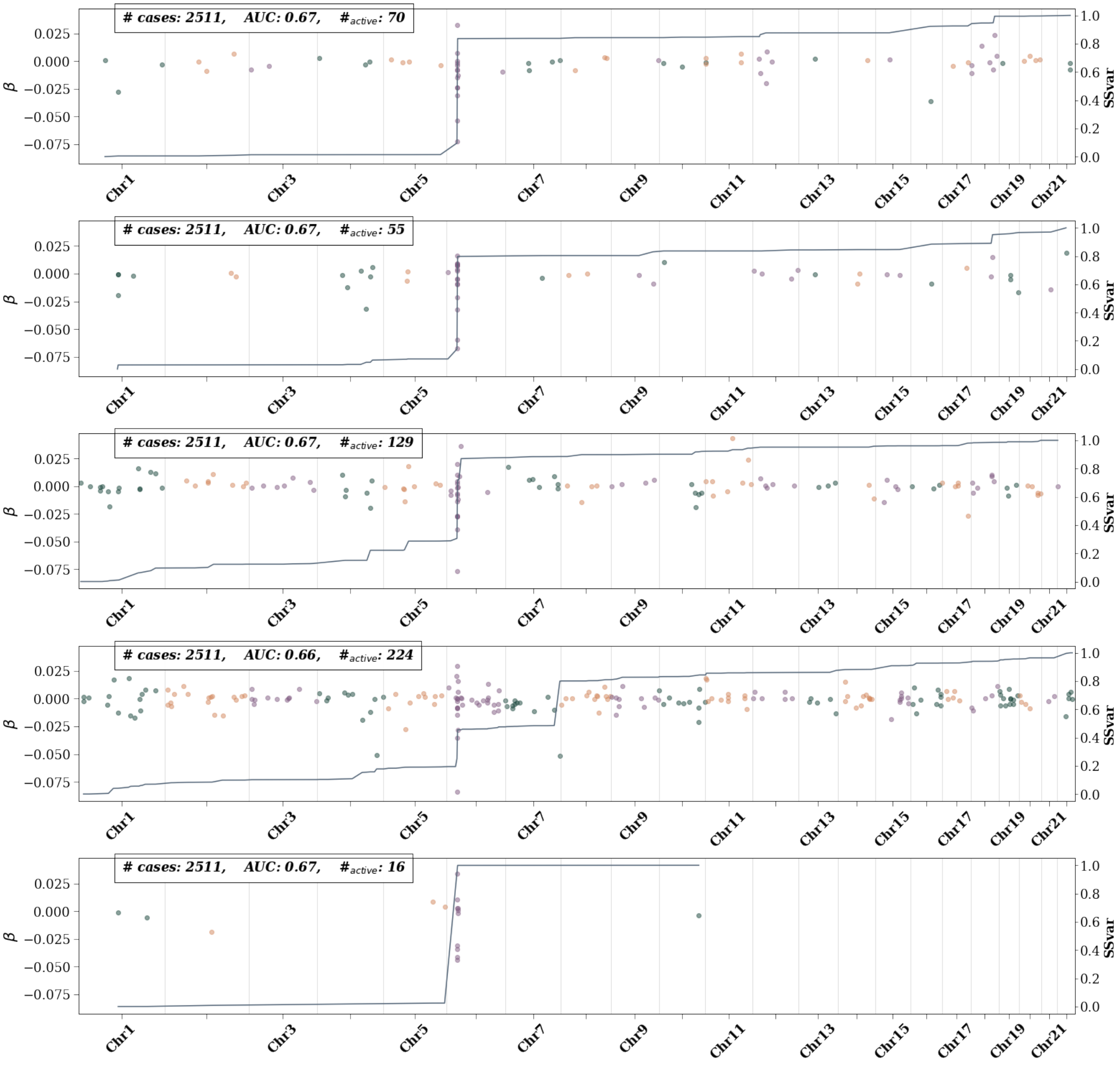

Figure 42: Atrial fibrillation active SNPs – i.e., SNPs with non-zero β weights– for 5 CV folds at near-maximum training size, but with equal cases and controls. Left axis shows the β value and is represented by colored dots. Different colors are used to differentiate chromosomes. The right axis represents the single SNP variance (SSV) normalized to the total SSV. The "training" label represents the number of cases used in training (an equal number of controls also used). Compared to maximal training in **Figure 41**, the features here are much more consistent across folds.

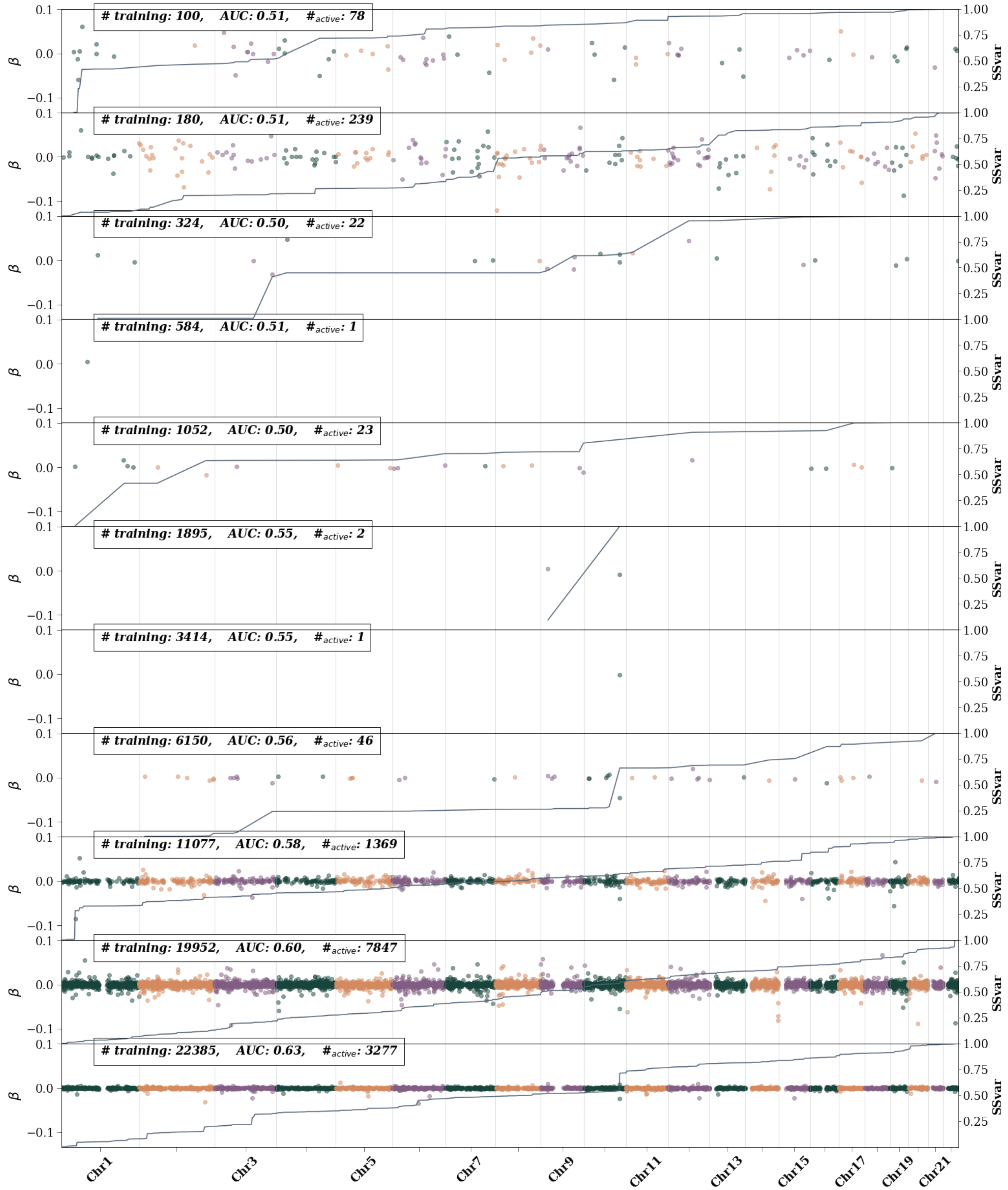

Figure 43: Type 2 diabetes active SNPs – i.e., SNPs with non-zero β weights– as training size is increased. The left axis shows the β value and is represented by colored dots. Different colors are used to differentiate chromosomes. The right axis represents the single SNP variance (SSV) normalized to the total SSV. The solid line showes the cumulative SSV. The "training" label represents the number of cases used in training. The first 10 (from the top) training sizes use equal number of cases and controls. The final training size uses all possible remaining controls

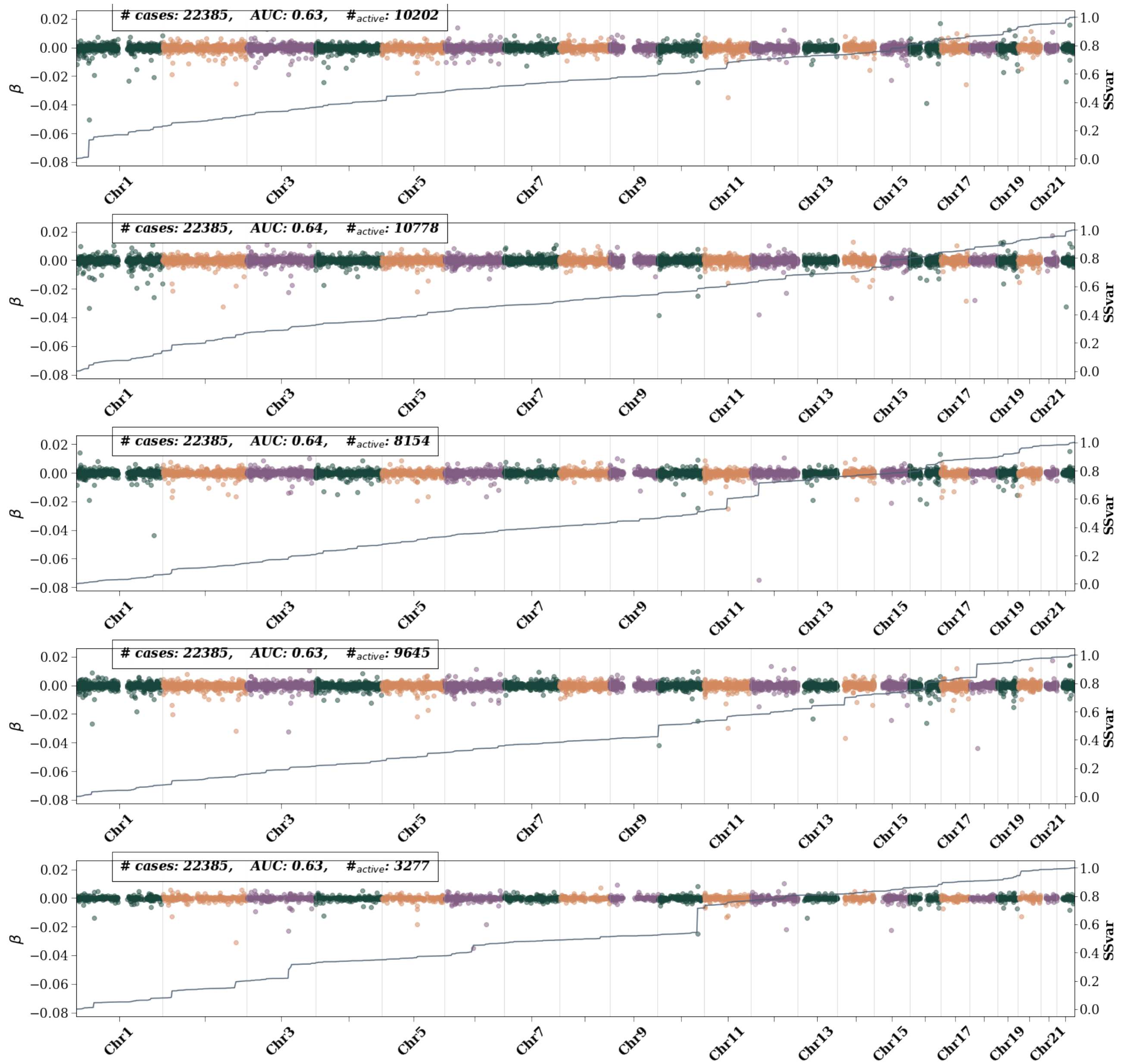

Figure 44: Type 2 diabetes active SNPs – i.e., SNPs with non-zero β weights– for 5 CV folds at maximum training size. Left axis shows the β value and is represented by colored dots. Different colors are used to differentiate chromosomes. The right axis represents the single SNP variance (SSV) normalized to the total SSV. The "training" label represents the number of cases used in training. All possible controls were used in each fold. While features generally appear consistent across folds, i.e., the presence of a bump in the SSV line, the size of the bump varies.

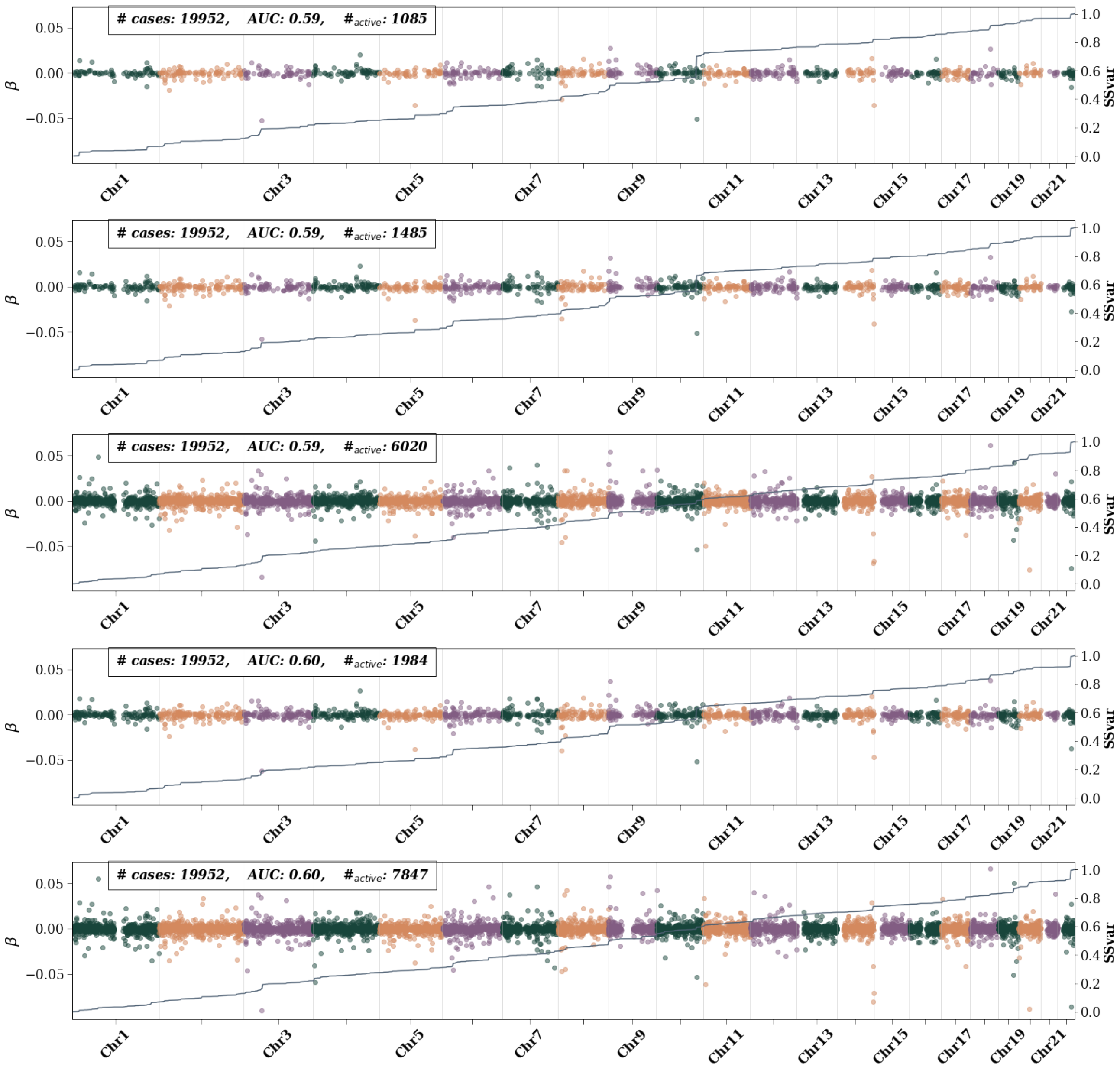

Figure 45: Atrial fibrillation active SNPs – i.e., SNPs with non-zero β weights– for 5 CV folds at near-maximum training size, but with equal cases and controls. Left axis shows the β value and is represented by colored dots. Different colors are used to differentiate chromosomes. The right axis represents the single SNP variance (SSV) normalized to the total SSV. The "training" label represents the number of cases used in training (an equal number of controls also used). Compared to maximal training in **Figure 44**, the features here are much more consistent across folds.

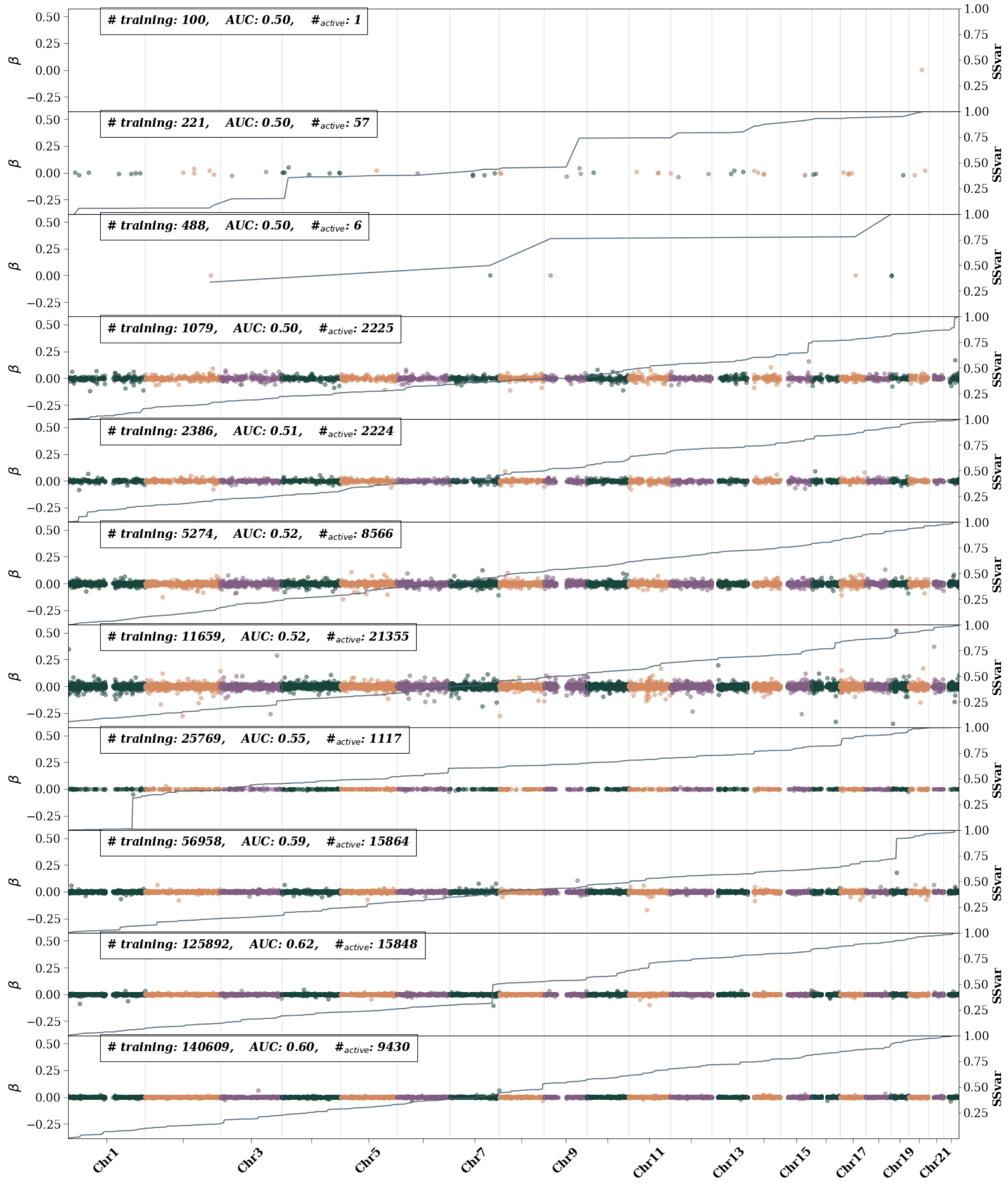

Figure 46: Hypertension active SNPs – i.e., SNPs with non-zero β weights– as training size is increased. The left axis shows the β value and is represented by colored dots. Different colors are used to differentiate chromosomes. The right axis represents the single SNP variance (SSV) normalized to the total SSV. The solid line showes the cumulative SSV. The "training" label represents the number of cases used in training. The first 10 (from the top) training sizes use equal number of cases and controls. The final training size uses all possible remaining controlse

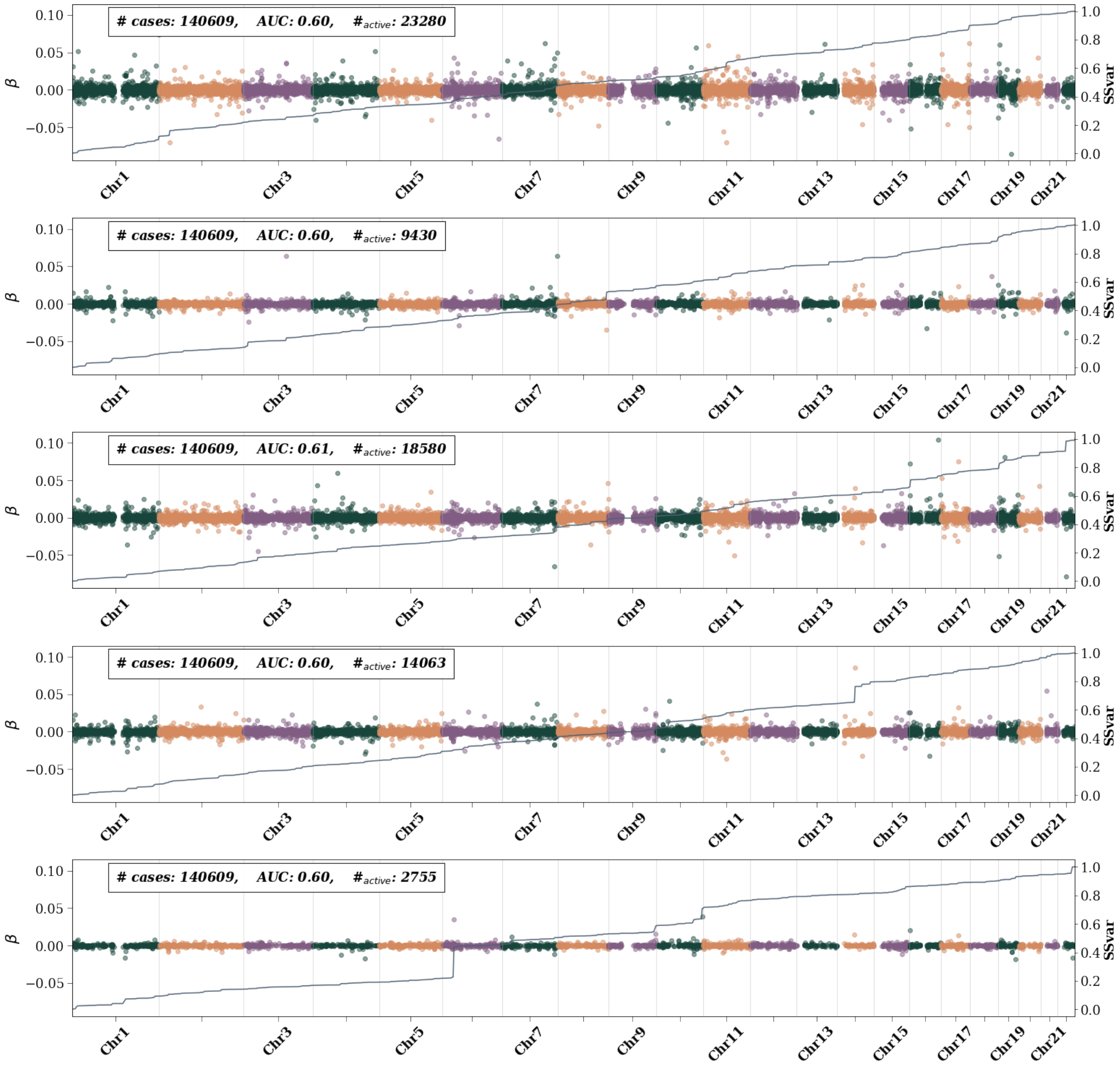

Figure 47: Hypertension active SNPs – i.e., SNPs with non-zero β weights– for 5 CV folds at maximum training size. Left axis shows the β value and is represented by colored dots. Different colors are used to differentiate chromosomes. The right axis represents the single SNP variance (SSV) normalized to the total SSV. The “training” label represents the number of cases used in training. All possible controls were used in each fold. While features generally appear consistent across folds, i.e., the presence of a bump in the SSV line, the size of the bump varies.

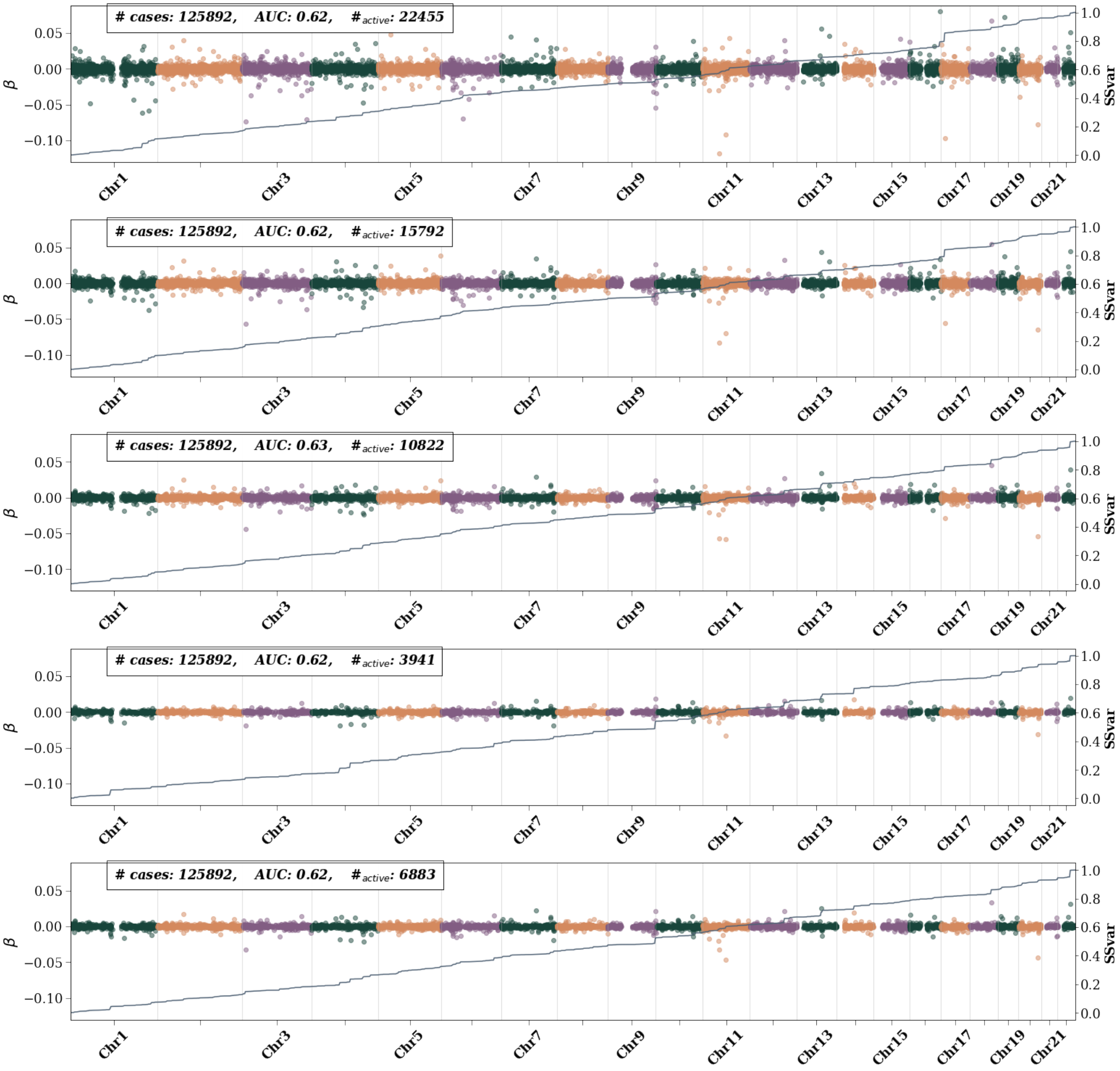

Figure 48: Atrial fibrillation active SNPs – i.e., SNPs with non-zero β weights– for 5 CV folds at near-maximum training size, but with equal cases and controls. Left axis shows the β value and is represented by colored dots. Different colors are used to differentiate chromosomes. The right axis represents the single SNP variance (SSV) normalized to the total SSV. The "training" label represents the number of cases used in training (an equal number of controls also used). Compared to maximal training in **Figure 47**, the features here are much more consistent across folds.

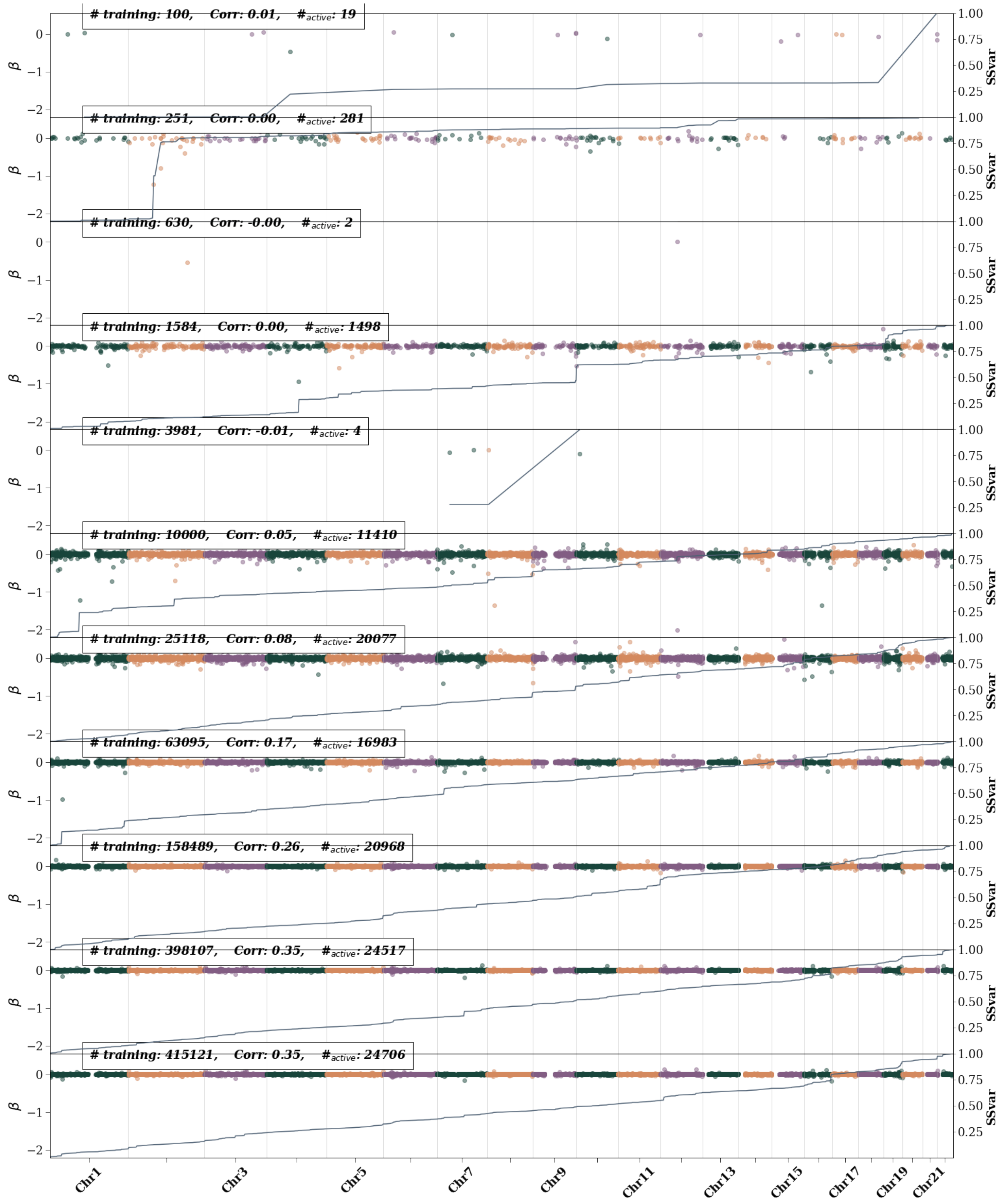

Figure 49: BMI active SNPs – i.e., SNPs with non-zero β weights– as training size is increased. The left axis shows the β value and is represented by colored dots. Different colors are used to differentiate chromosomes. The right axis represents the single SNP variance (SSV) normalized to the total SSV. The solid line showes the cumulative SSV. The "training" label represents the number of cases used in training. The first 10 (from the top) training sizes use equal number of cases and controls. The final training size uses all possible remaining controlse

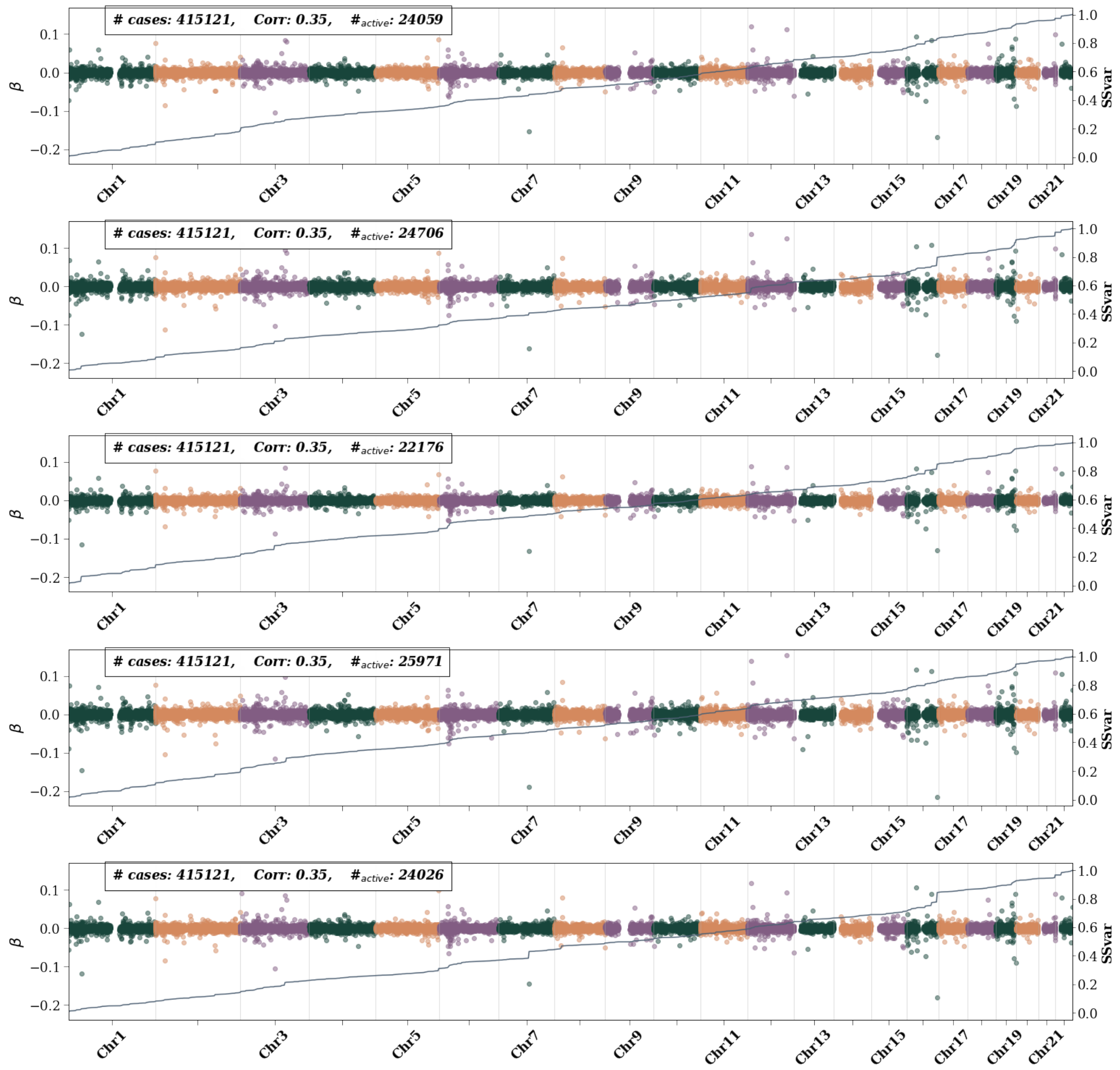

Figure 50: BMI active SNPs – i.e., SNPs with non-zero β weights– for 5 CV folds at maximum training size. Left axis shows the β value and is represented by colored dots. Different colors are used to differentiate chromosomes. The right axis represents the single SNP variance (SSV) normalized to the total SSV. The "training" label represents the number of cases used in training. All possible controls were used in each fold. While features generally appear consistent across folds, i.e., the presence of a bump in the SSV line, the size of the bump varies.

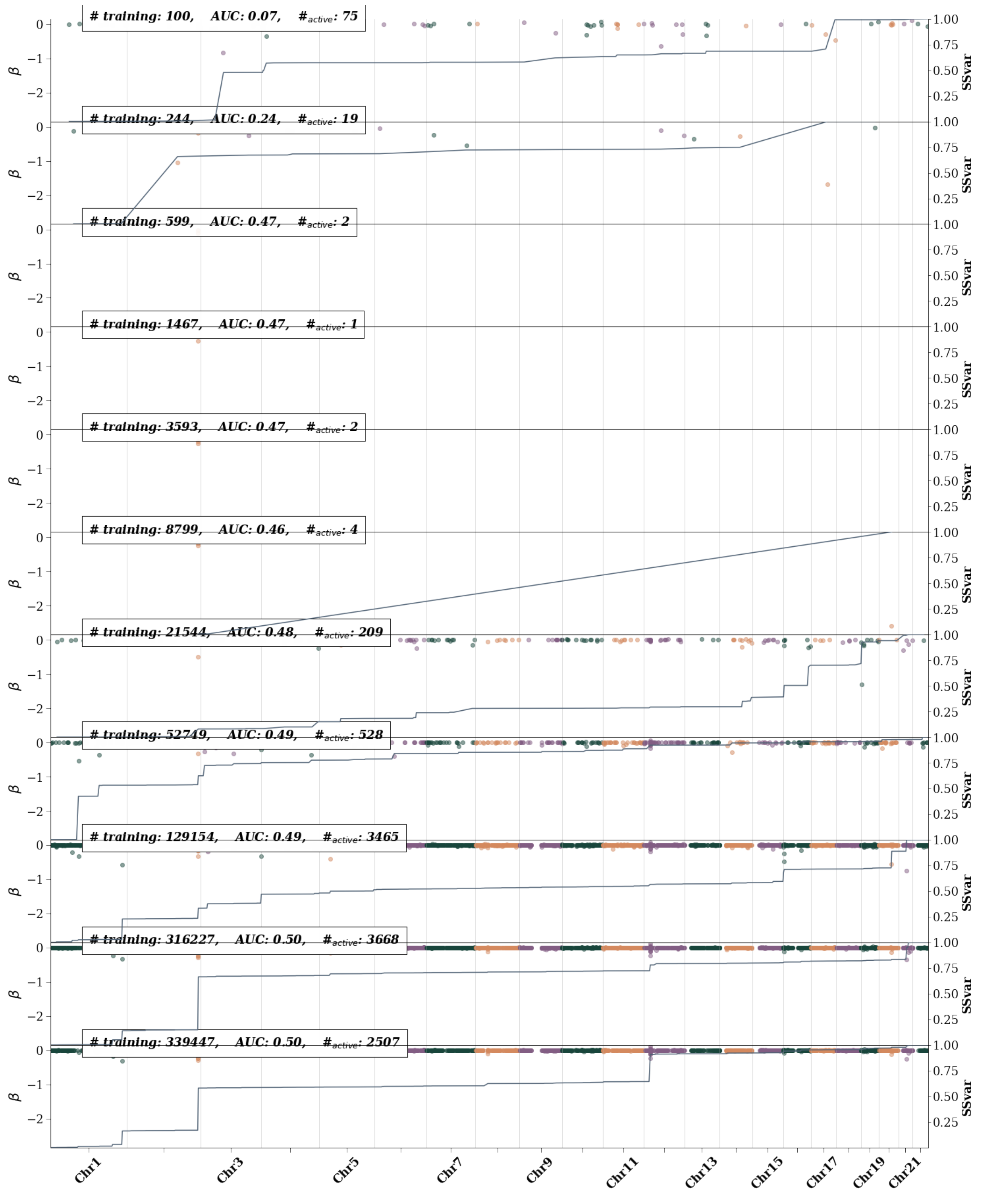

Figure 51: Direct bilirubin active SNPs – i.e., SNPs with non-zero β weights– as training size is increased. The left axis shows the β value and is represented by colored dots. Different colors are used to differentiate chromosomes. The right axis represents the single SNP variance (SSV) normalized to the total SSV. The solid line showes the cumulative SSV. The "training" label represents the number of cases used in training. The first 10 (from the top) training sizes use equal number of cases and controls. The final training size uses all possible remaining controls

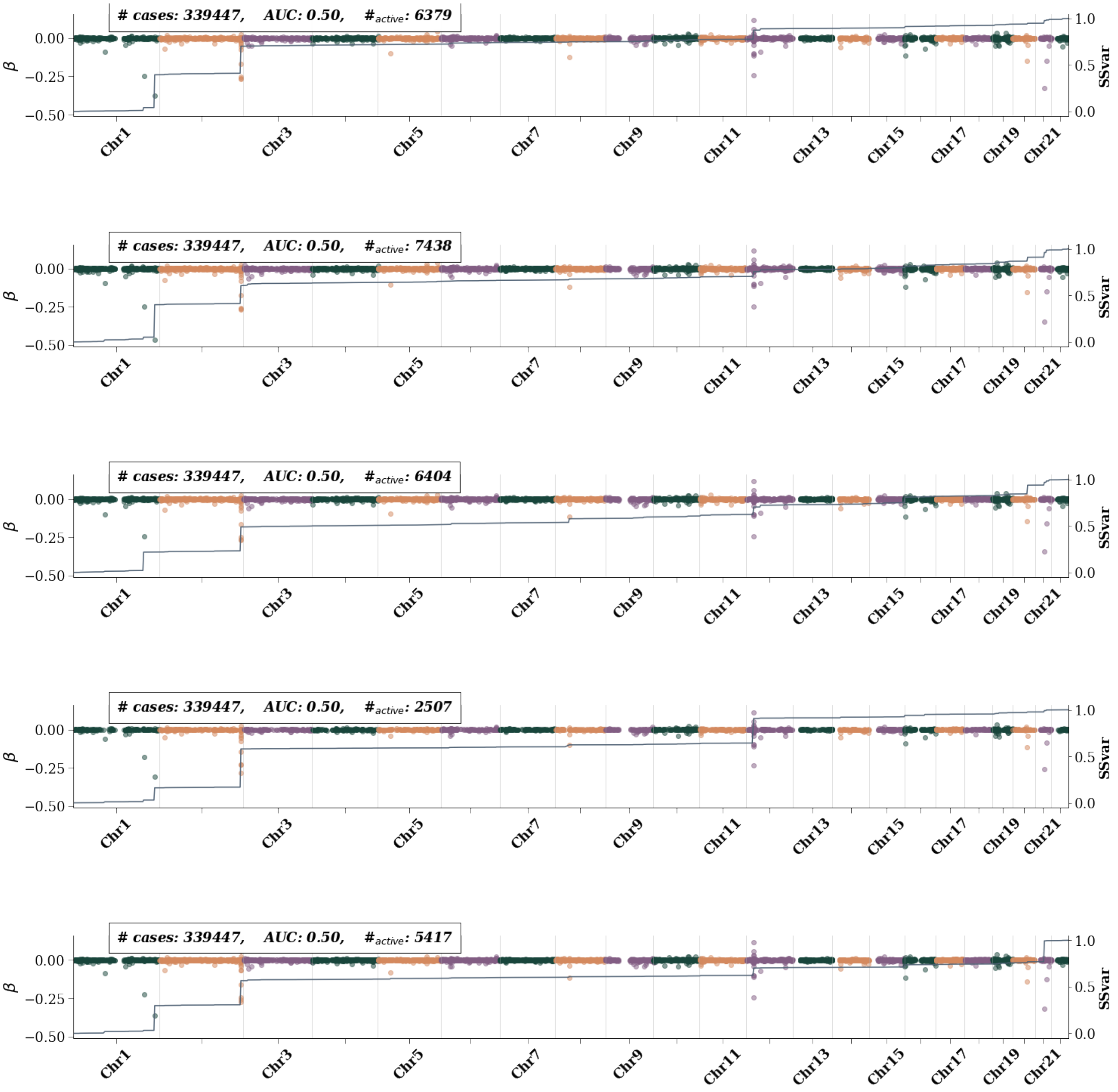

Figure 52: Direct bilirubin active SNPs – i.e., SNPs with non-zero β weights– for 5 CV folds at maximum training size. Left axis shows the β value and is represented by colored dots. Different colors are used to differentiate chromosomes. The right axis represents the single SNP variance (SSV) normalized to the total SSV. The "training" label represents the number of cases used in training. All possible controls were used in each fold. While features generally appear consistent across folds, i.e., the presence of a bump in the SSV line, the size of the bump varies.

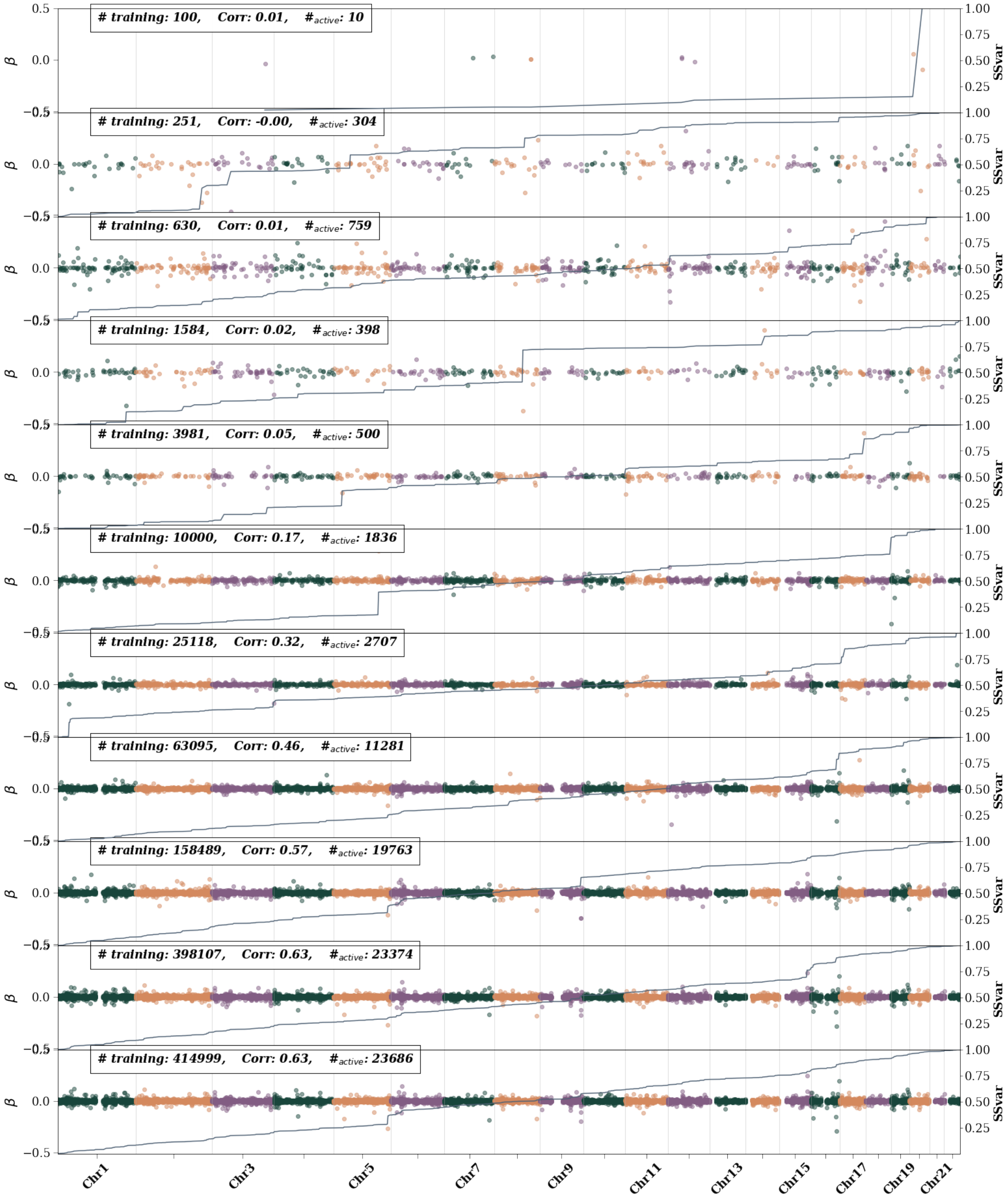

Figure 53: Height active SNPs – i.e., SNPs with non-zero β weights– as training size is increased. The left axis shows the β value and is represented by colored dots. Different colors are used to differentiate chromosomes. The right axis represents the single SNP variance (SSV) normalized to the total SSV. The solid line showes the cumulative SSV. The "training" label represents the number of cases used in training. The first 10 (from the top) training sizes use equal number of cases and controls. The final training size uses all possible remaining controls

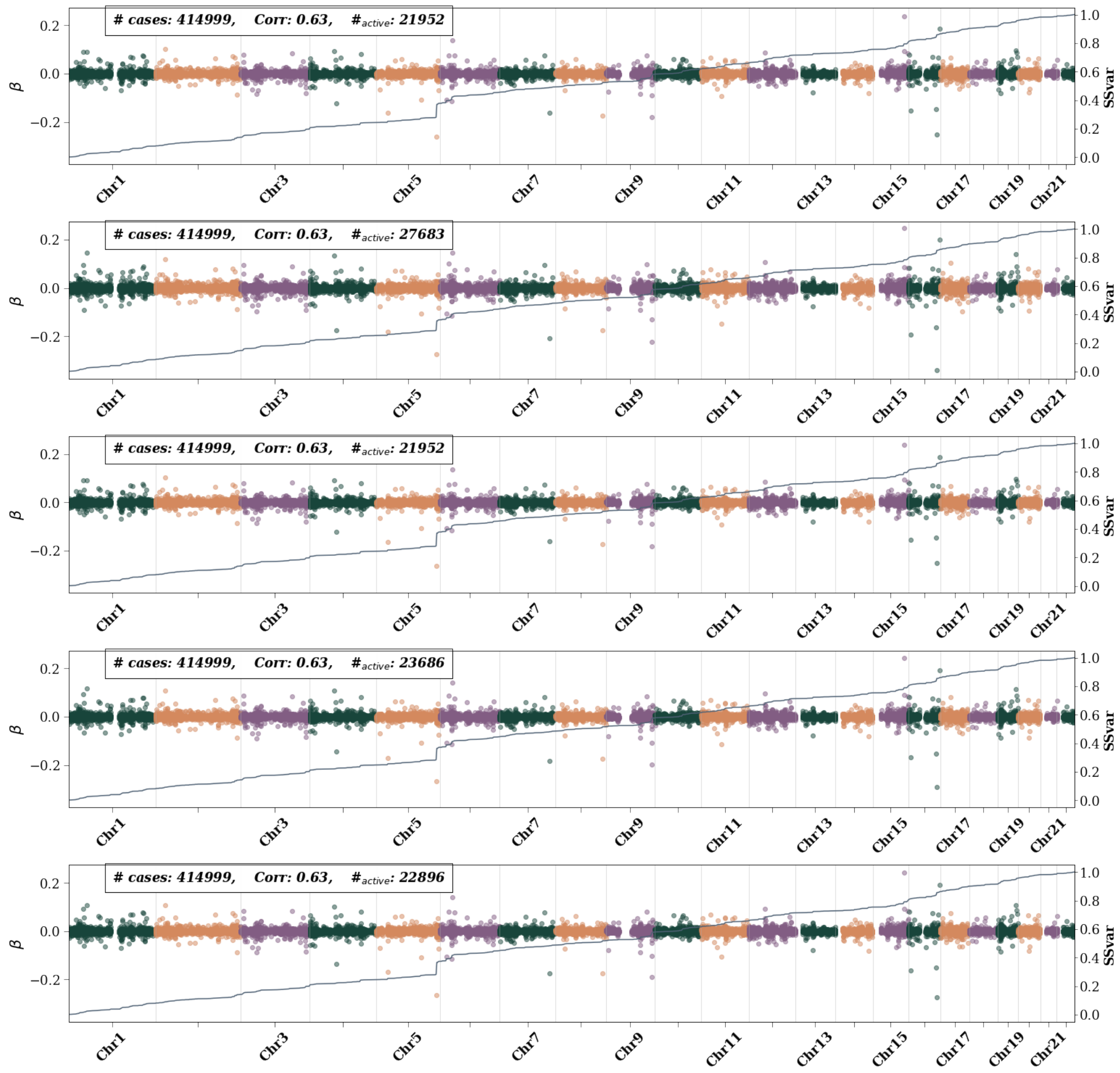

Figure 54: Height active SNPs – i.e., SNPs with non-zero β weights– for 5 CV folds at maximum training size. Left axis shows the β value and is represented by colored dots. Different colors are used to differentiate chromosomes. The right axis represents the single SNP variance (SSV) normalized to the total SSV. The “training” label represents the number of cases used in training. All possible controls were used in each fold. While features generally appear consistent across folds, i.e., the presence of a bump in the SSV line, the size of the bump varies.

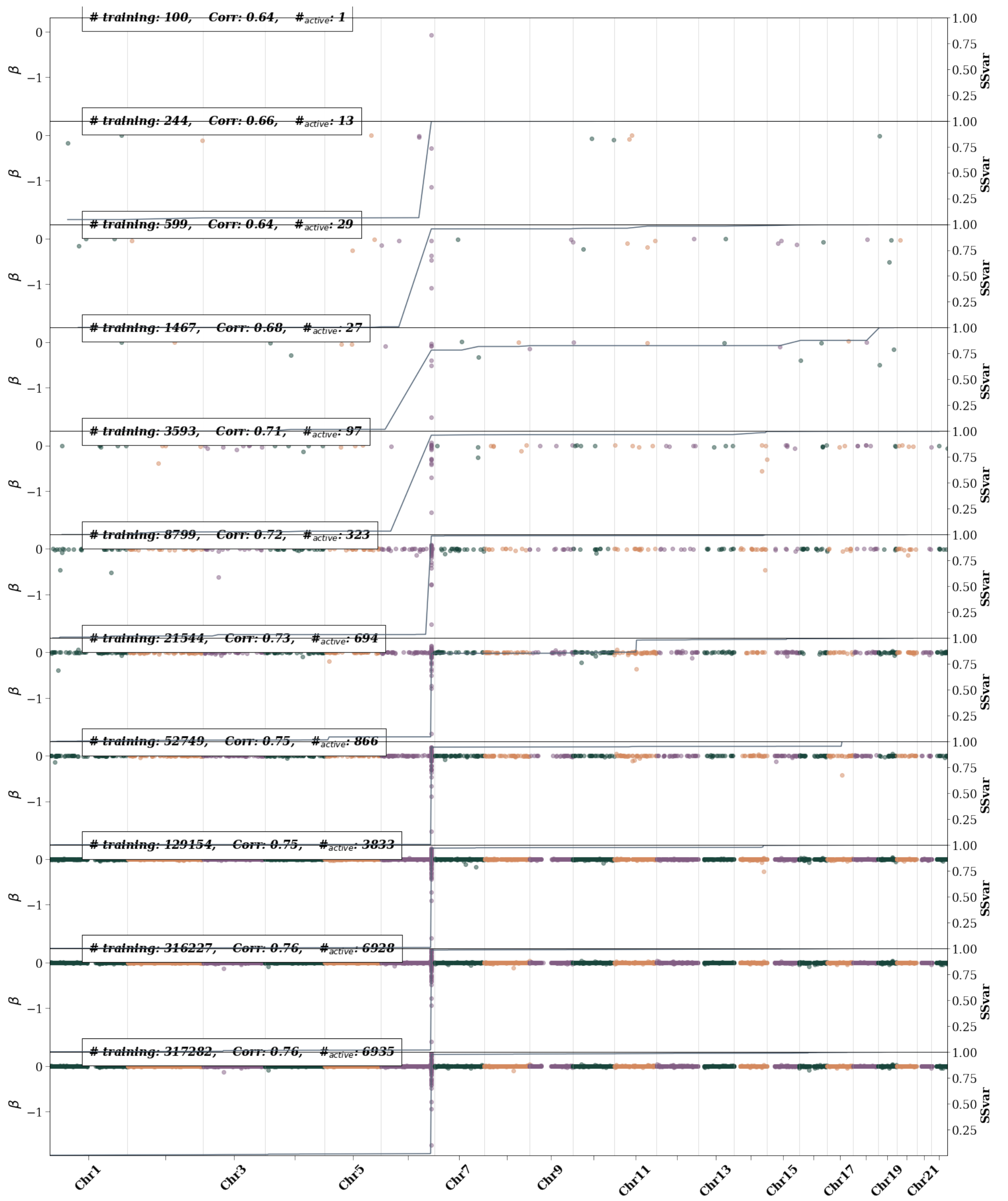

Figure 55: Lipoprotein A active SNPs – i.e., SNPs with non-zero β weights– as training size is increased. The left axis shows the β value and is represented by colored dots. Different colors are used to differentiate chromosomes. The right axis represents the single SNP variance (SSV) normalized to the total SSV. The solid line showes the cumulative SSV. The "training" label represents the number of cases used in training. The first 10 (from the top) training sizes use equal number of cases and controls. The final training size uses all possible remaining controls

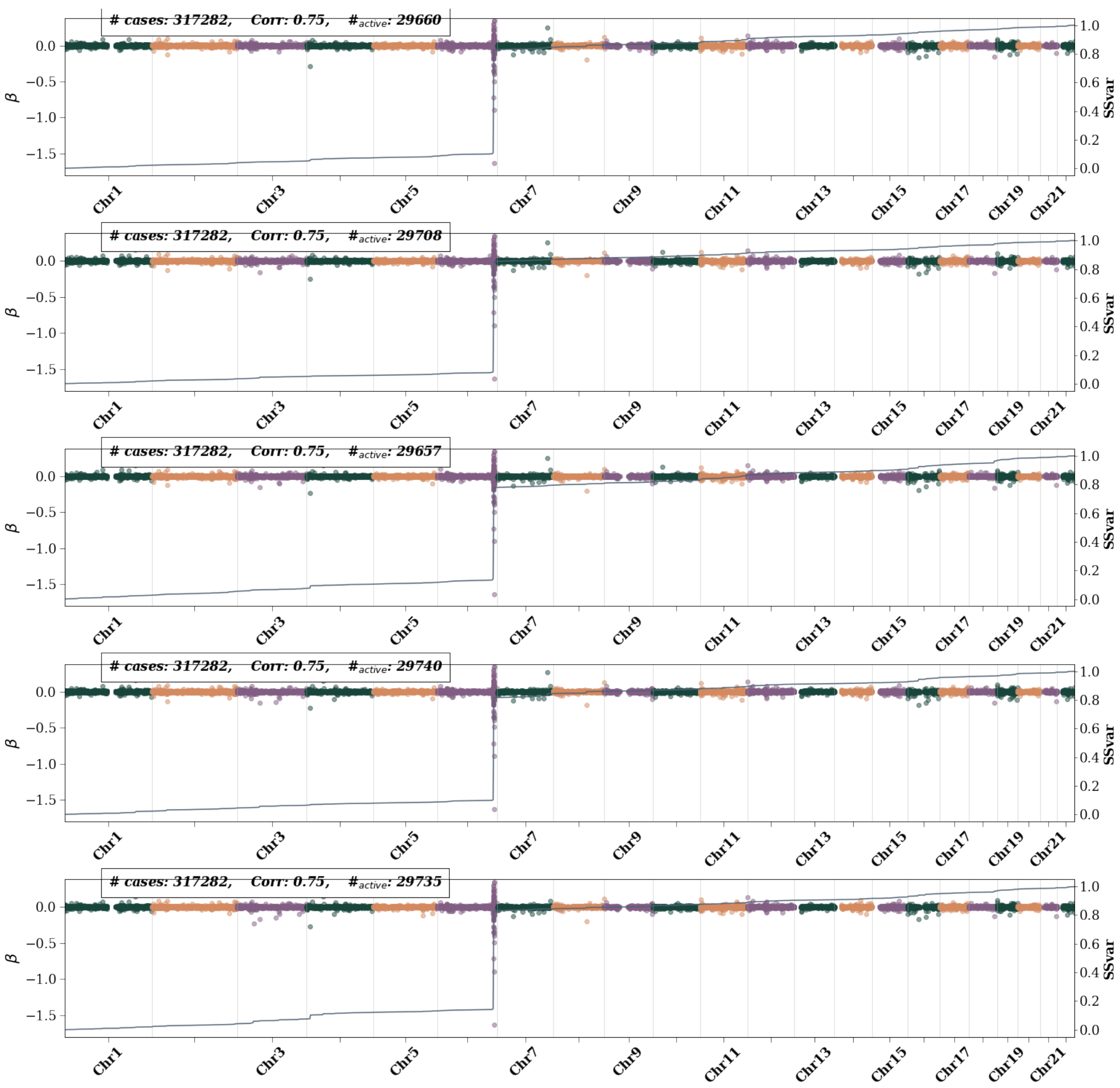

Figure 56: Lipoprotein A active SNPs – i.e., SNPs with non-zero β weights– for 5 CV folds at maximum training size. Left axis shows the β value and is represented by colored dots. Different colors are used to differentiate chromosomes. The right axis represents the single SNP variance (SSV) normalized to the total SSV. The “training” label represents the number of cases used in training. All possible controls were used in each fold. While features generally appear consistent across folds, i.e., the presence of a bump in the SSV line, the size of the bump varies.

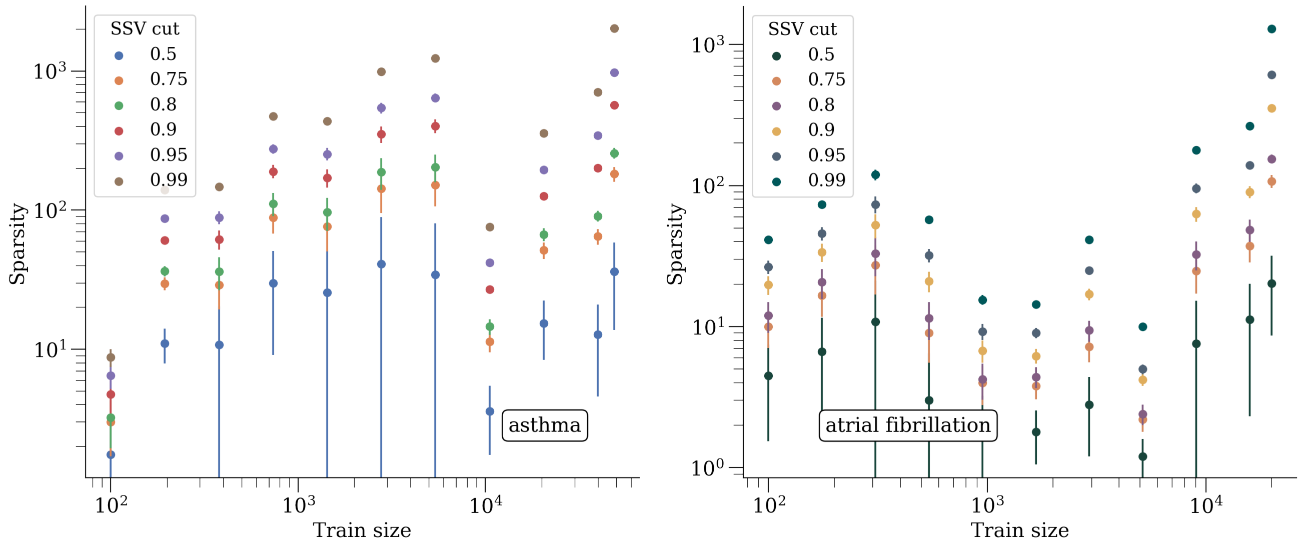

Figure 57: Sparsity as a function of training size, after keeping SNPs that account for 50% - 99% of SSV.

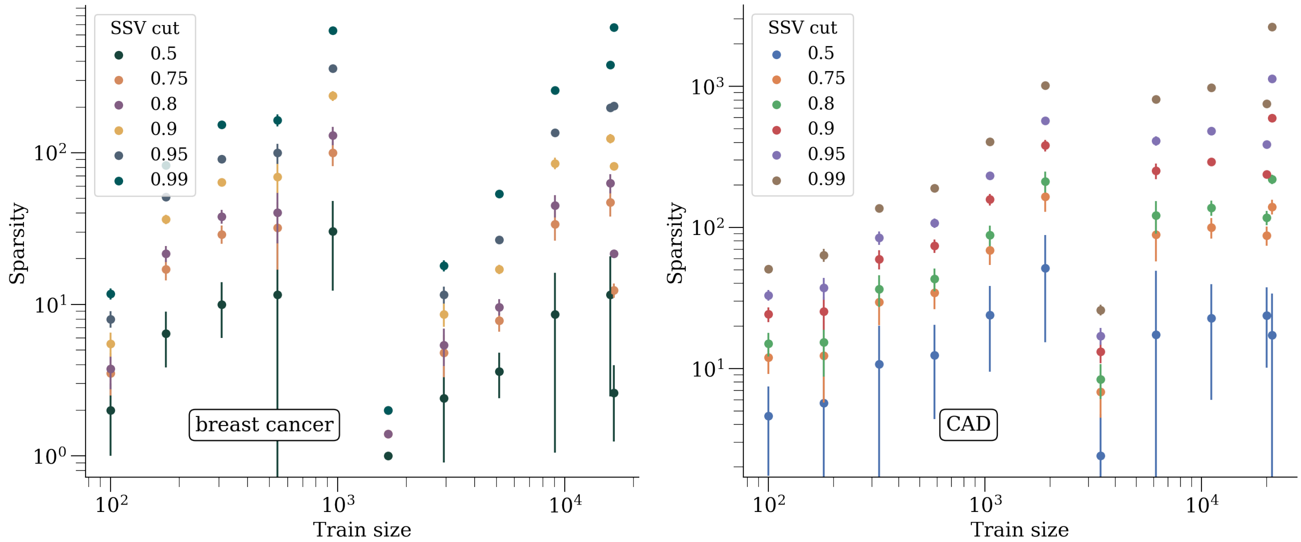

Figure 58: Sparsity as a function of training size, after keeping SNPs that account for 50% - 99% of SSV.

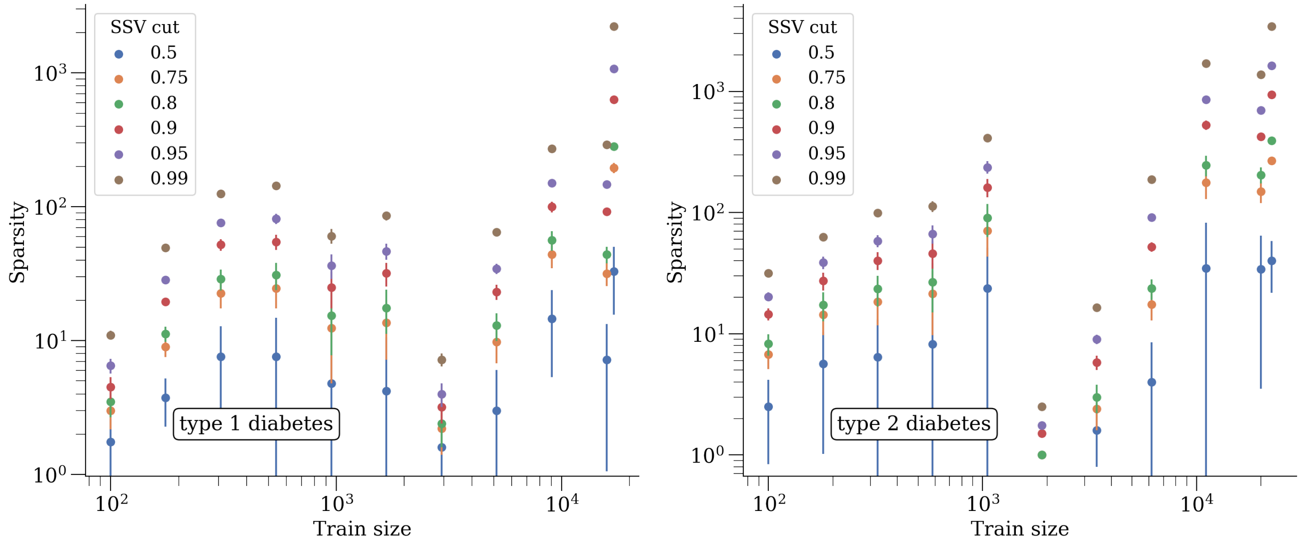

Figure 59: Sparsity as a function of training size, after keeping SNPs that account for 50% - 99% of SSV.

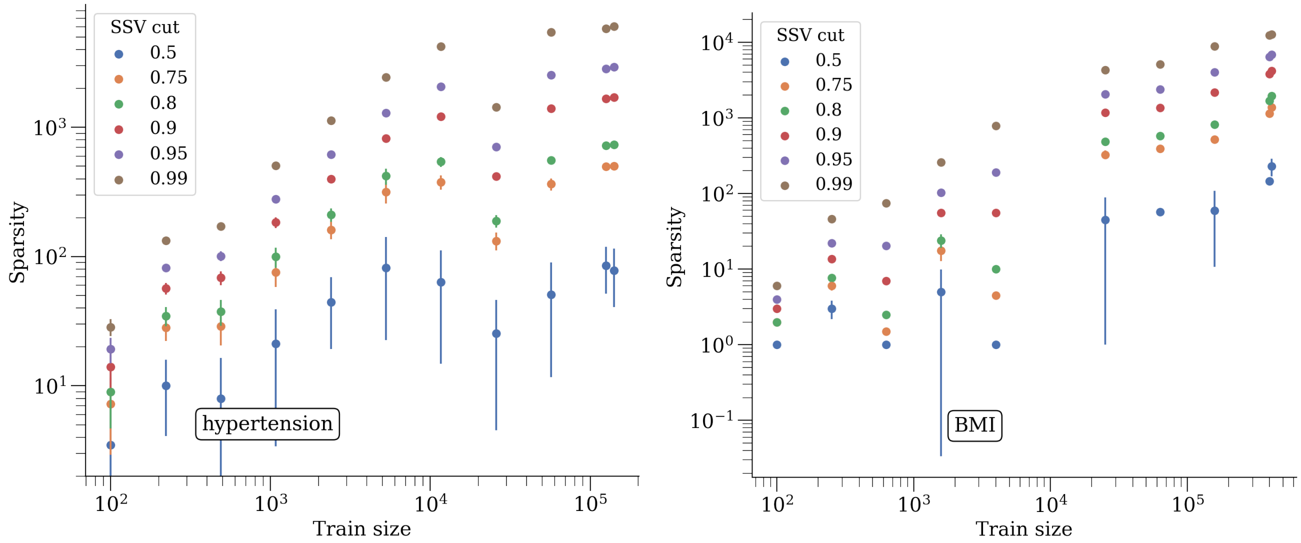

Figure 60: Sparsity as a function of training size, after keeping SNPs that account for 50% - 99% of SSV.

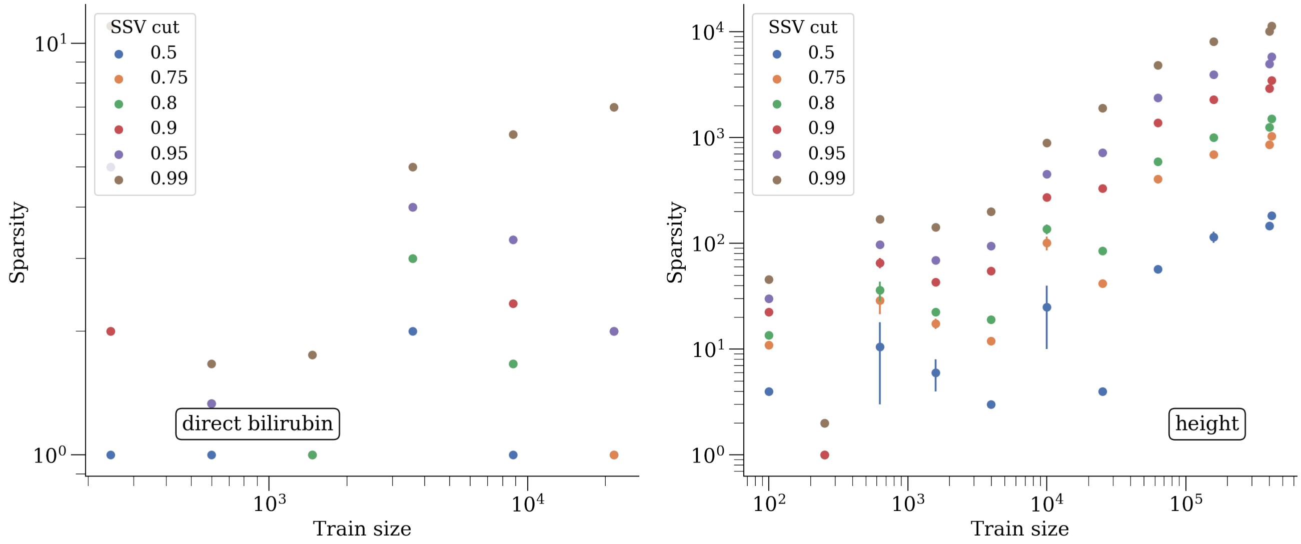

Figure 61: Sparsity as a function of training size, after keeping SNPs that account for 50% - 99% of SSV.

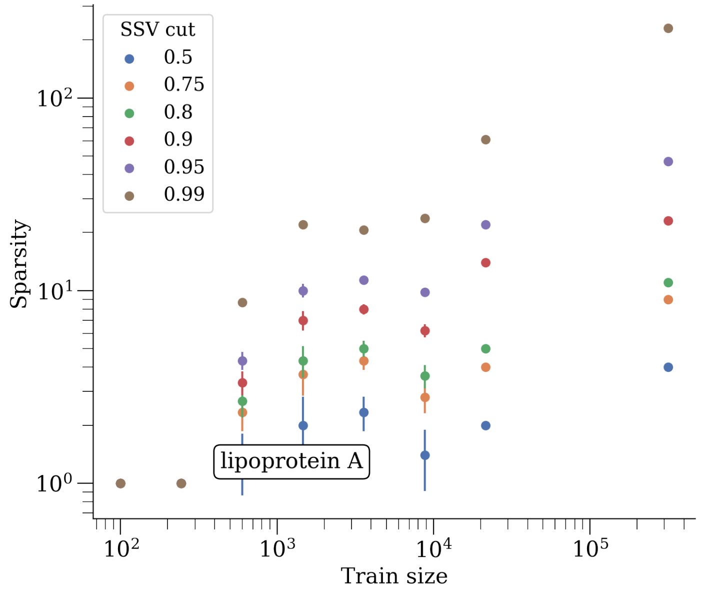

Figure 62: Sparsity as a function of training size, after keeping SNPs that account for 50% - 99% of SSV.

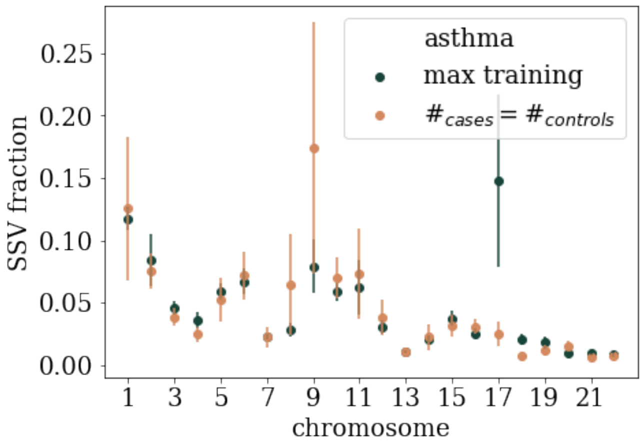

Figure 63: Average SSV per chromosome for asthma. Uncertainty comes from averaging over 5 fold cross validation. Max training refers to using the maximum number of cases and all possible controls. $\#_{cases} = \#_{controls}$ uses near maximal number of cases and an equal number of controls. Both types of training find generally similar SSV distribution, but max training finds a much larger signal on chromosome 17 and a smaller signal on chromosome 9.

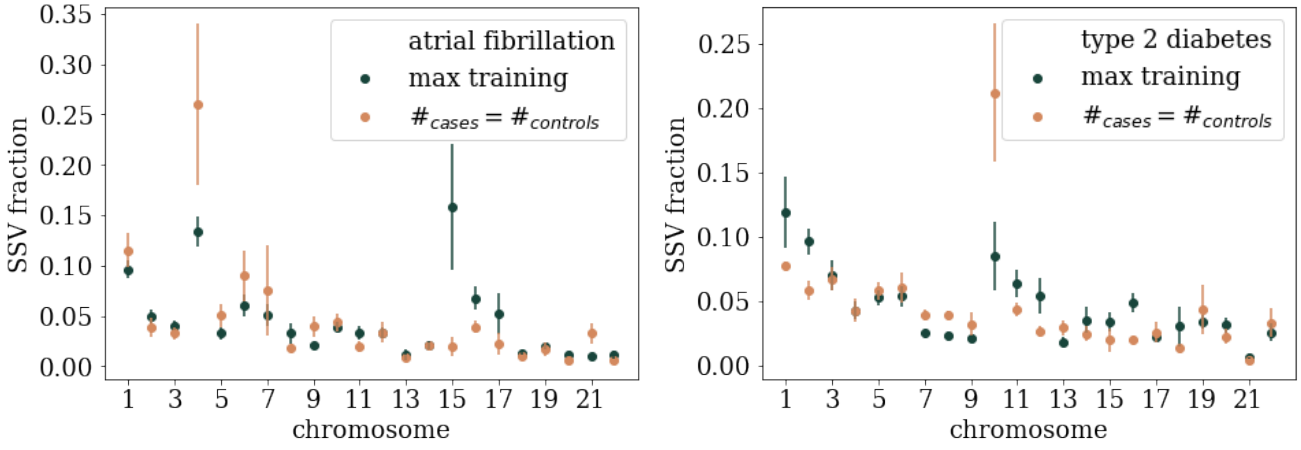

Figure 64: Fraction of SSV per chromosome for max training and near-max number of cases and equal number of controls.

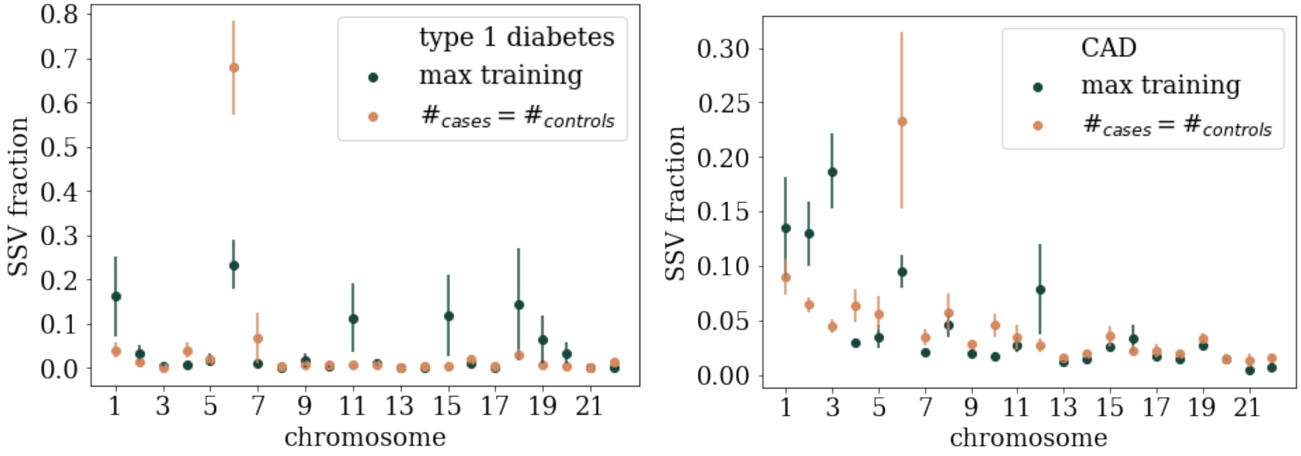

Figure 65: Fraction of SSV per chromosome for max training and near-max number of cases and equal number of controls.

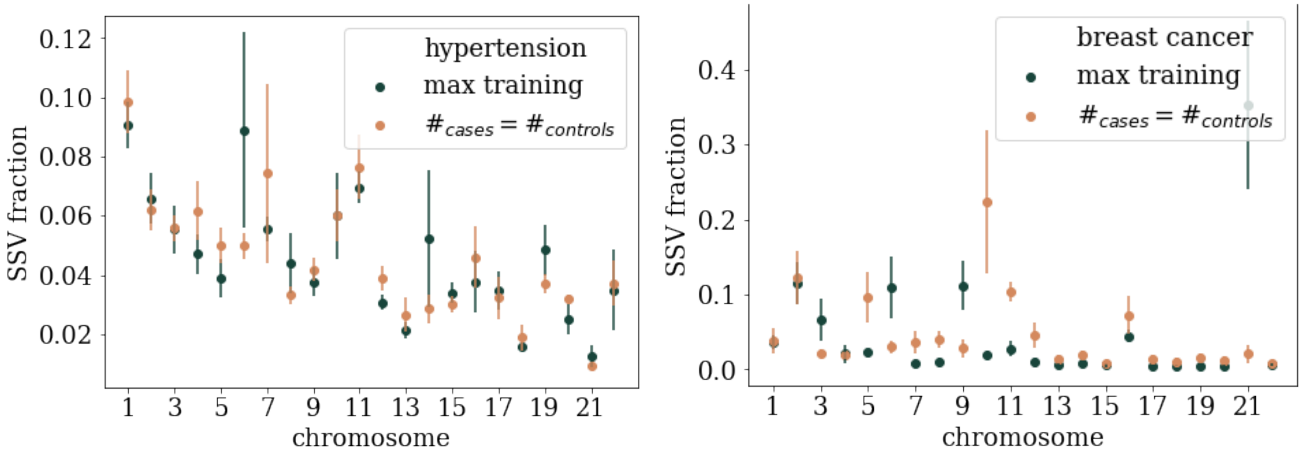

Figure 66: Fraction of SSV per chromosome for max training and near-max number of cases and equal number of controls.

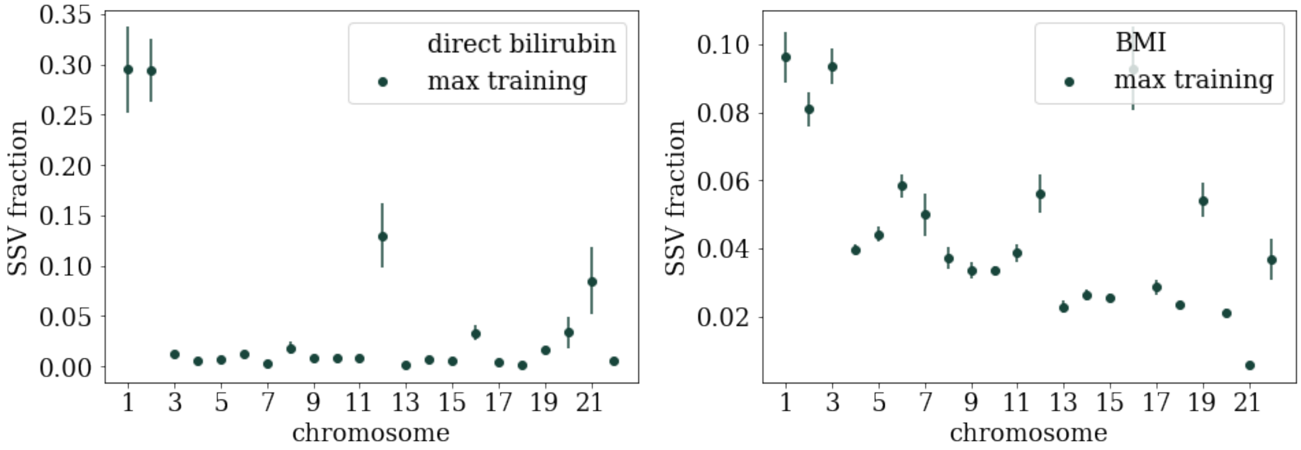

Figure 67: Fraction of SSV per chromosome for max training.

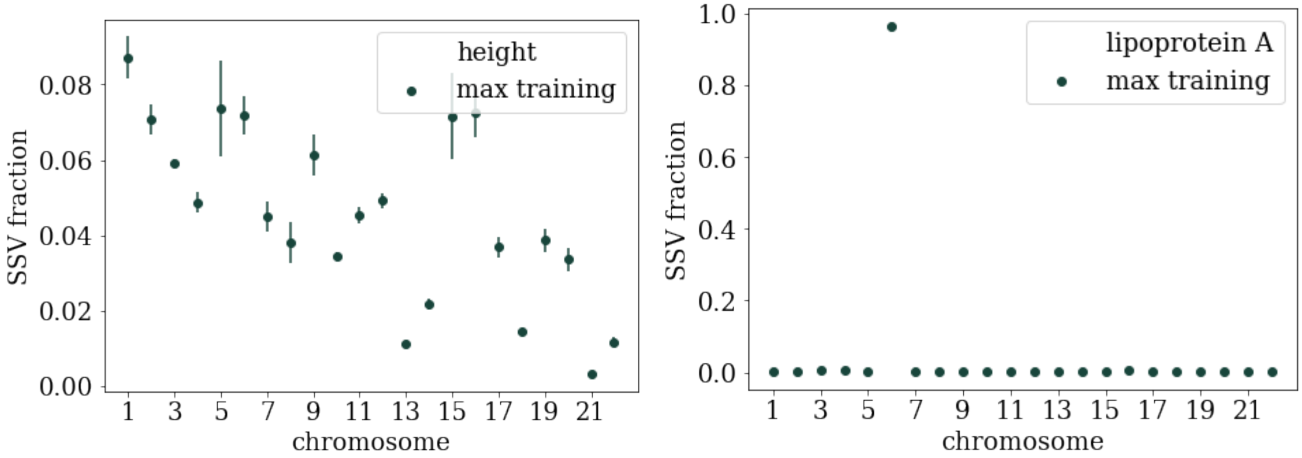

Figure 68: Fraction of SSV per chromosome for max training.

8 Odds ratios

In the main text we detail how to compute an inclusive odds ratio and demonstrate using asthma. In **Figure 69-Figure 71** we show the odds ratios for the remaining case-control phenotypes.

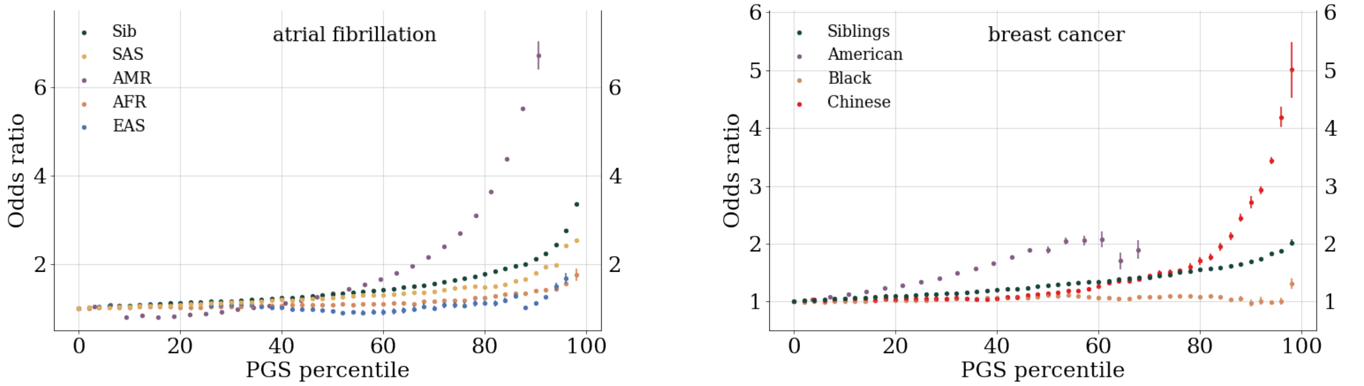

Figure 69: Odds ratio for atrial fibrillation and breast cancer.

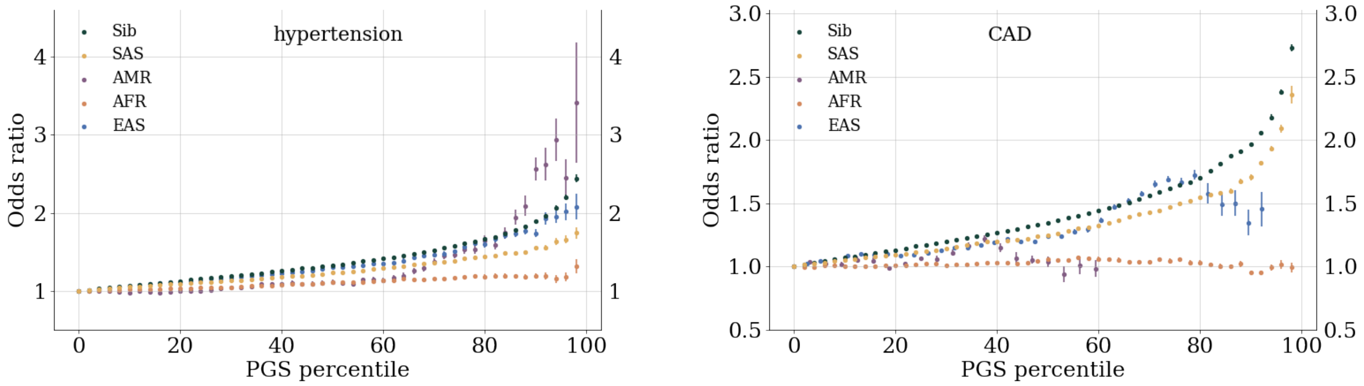

Figure 70: Odds ratio for hypertension and CAD.

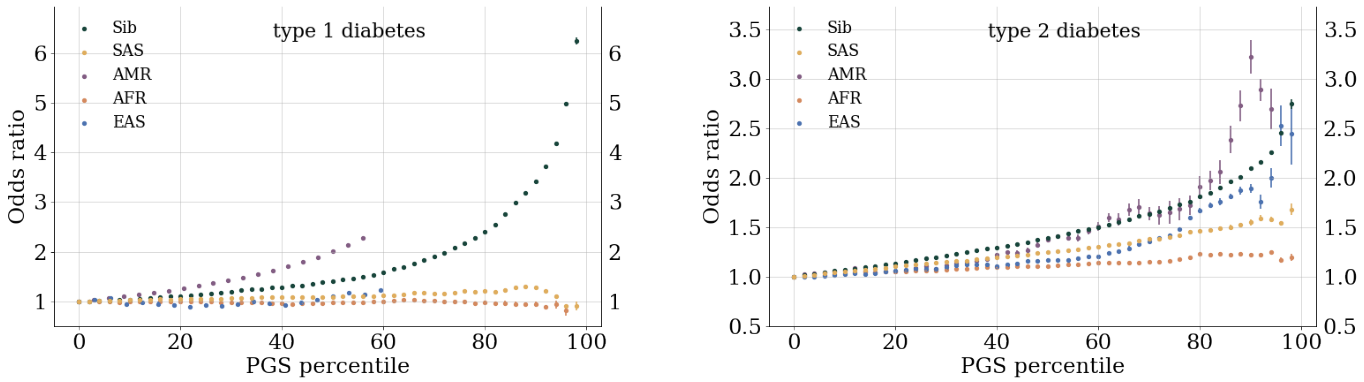

Figure 71: Odds ratio for type 1 and 2 diabetes.

9 Computing details

Here we collect plots of the computing performance for the LASSO computations used in this project as an example of how computationally intense this project is. Computing was all done on a cluster using SLURM workload manager. Jobs were monitored using the `seff` command after they finished running. On the right hand side of **Figure 72** there is a slightly odd linear cluster of points around 350GB. This appear to be an artifact of cluster maintenance setting some job metadata to this default value. These data points were included for completeness.

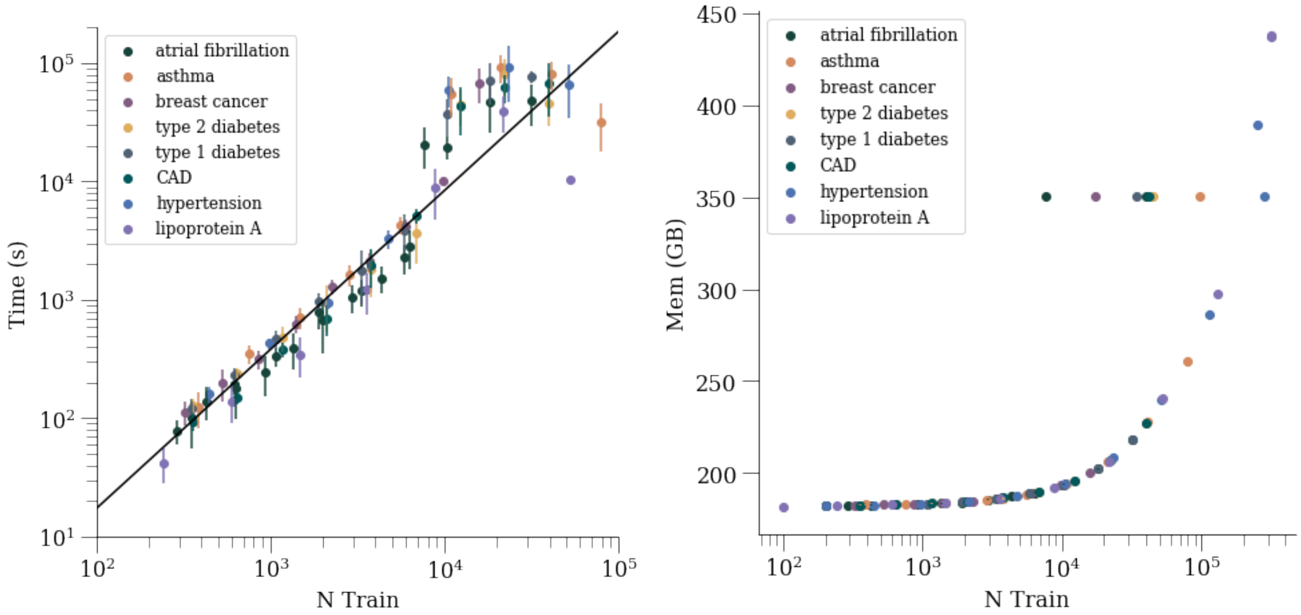

Figure 72: Time and memory usage as a function of training size for LASSO training.

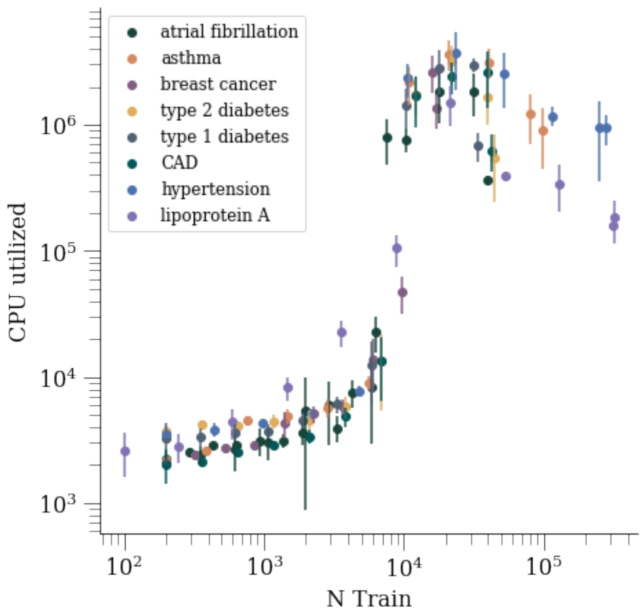

Figure 73: CPU utilization as a function of training size for LASSO training.

References

1. Alexander, D. H., Novembre, J. & Lange, K. Fast model-based estimation of ancestry in unrelated individuals. *Genome research* **19,** 1655–1664 (2009) (cit. on p. 1).
2. Manichaikul, A. *et al.* Robust relationship inference in genome-wide association studies. *Bioinformatics* **26,** 2867–2873. ISSN: 1367-4803. eprint: `https://academic.oup.com/bioinformatics/article-pdf/26/22/2867/48853490/bioinformatics\_26\_22\_2867.pdf`. `https://doi.org/10.1093/bioinformatics/btq559` (Oct. 2010) (cit. on p. 1).
3. Bycroft, C., Freeman, C. & Petkova, D. The UK Biobank resource with deep phenotyping and genomic data. *Nature* **562,** 203–209 (cit. on p. 1).
4. *Current Asthma Demographics* `https://www.lung.org/research/trends-in-lung-disease/asthma-trends-brief/current-demographics`. Accessed: 2022-08-23 (cit. on p. 2).
5. *Asthma Prevalence, Health Care Use and Mortality: United States, 2003-05* `https://www.cdc.gov/nchs/data/hestat/asthma03-05/asthma03-05.htm`. Accessed: 2022-08-23 (cit. on p. 2).
6. Huang, J.-L. Asthma severity and genetics in Taiwan. *Journal of Microbiology, Immunology, and Infection= Wei Mian yu gan ran za zhi* **38,** 158–163 (2005) (cit. on p. 2).
7. Jan, I, Chou, W.-H., Wang, J.-D., Kuo, S.-H., *et al.* Prevalence of and major risk factors for adult bronchial asthma in Taipei City. *Journal of the Formosan Medical Association* **103,** 259–263 (2004) (cit. on p. 2).
8. Rosser, F. J., Forno, E., Cooper, P. J. & Celedón, J. C. Asthma in Hispanics. An 8-year update. *American journal of respiratory and critical care medicine* **189,** 1316–1327 (2014) (cit. on p. 2).
9. Alonso, A. *et al.* Incidence of atrial fibrillation in whites and African-Americans: the Atherosclerosis Risk in Communities (ARIC) study. *American heart journal* **158,** 111–117 (2009) (cit. on p. 2).
10. Chiang, C.-E. *et al.* 2016 Guidelines of the Taiwan Heart Rhythm Society and the Taiwan Society of Cardiology for the management of atrial fibrillation. *Journal of the Formosan Medical Association* **115,** 893–952 (2016) (cit. on p. 2).
11. Linares, J. D. *et al.* Prevalence of atrial fibrillation and association with clinical, sociocultural, and ancestral correlates among Hispanic/Latinos: The Hispanic Community Health Study/Study of Latinos. *Heart rhythm* **16,** 686–693 (2019) (cit. on p. 3).
12. Hunt, B. R. Breast cancer prevalence and mortality among Hispanic subgroups in the United States, 2009–2013. *Journal of Cancer Epidemiology* **2016** (2016) (cit. on p. 3).

13. *American Cancer Society. Cancer Facts & Figures for African Americans 2019-2021. Atlanta: American Cancer Society, 2019.* https://www.cancer.org/content/dam/cancer-org/research/cancer-facts-and-statistics/cancer-facts-and-figures-for-african-americans/cancer-facts-and-figures-for-african-americans-2019-2021.pdf. Accessed: 2022-08-23 (cit. on p. 3).

14. Liu, F.-C. *et al.* Epidemiology and survival outcome of breast cancer in a nationwide study. *Oncotarget* **8,** 16939 (2017) (cit. on p. 3).

15. Khera, A. V. *et al.* Genome-wide polygenic scores for common diseases identify individuals with risk equivalent to monogenic mutations. *Nature Genetics* **50,** 1219 (2018) (cit. on p. 3).

16. Lee, Y.-T. *et al.* Chin-Shan Community Cardiovascular Cohort in Taiwan–baseline data and five-year follow-up morbidity and mortality. *Journal of clinical epidemiology* **53,** 838–846 (2000) (cit. on p. 3).

17. *CDC 2021. Summary Health Statistics: National Health Interview Survey: 2018. Table A-1a.* https://www.cdc.gov/nchs/nhis/shs/tables.htm and https://ftp.cdc.gov/pub/Health_Statistics/NCHS/NHIS/SHS/2018_SHS_Table_A-1.pdf. Accessed: 2022-08-23 (cit. on p. 3).

18. Lackland, D. T. Racial differences in hypertension: implications for high blood pressure management. *The American journal of the medical sciences* **348,** 135–138 (2014) (cit. on p. 3).

19. Pan, H.-Y., Lin, H.-J., Chen, W.-J. & Wang, T.-D. Prevalence, treatment, control and monitoring of hypertension: a nationwide community-based survey in Taiwan, 2017. *Acta Cardiologica Sinica* **36,** 375 (2020) (cit. on p. 3).

20. Lora, C. M. *et al.* Prevalence, awareness, and treatment of hypertension in hispanics/latinos with CKD in the Hispanic Community Health Study/Study of Latinos. *Kidney medicine* **2,** 332–340 (2020) (cit. on p. 3).

21. Borchers, A. T., Uibo, R. & Gershwin, M. E. The geoepidemiology of type 1 diabetes. *Autoimmunity reviews* **9,** A355–A365 (2010) (cit. on p. 3).

22. Kinney, G. L. *et al.* The prevalence of type 1 diabetes in Hispanic/Latino populations in the United States: findings from the Hispanic Community Health Study/Study of Latinos. *Epidemiology* **31,** e7–e8 (2020) (cit. on p. 3).

23. Jiang, Y.-D., Chang, C.-H., Tai, T.-Y., Chen, J.-F. & Chuang, L.-M. Incidence and prevalence rates of diabetes mellitus in Taiwan: analysis of the 2000–2009 Nationwide Health Insurance database. *Journal of the Formosan Medical Association* **111,** 599–604 (2012) (cit. on p. 3).

24. Lu, C.-L., Shen, H.-N., Chen, H.-F. & Li, C.-Y. Epidemiology of childhood Type 1 diabetes in Taiwan, 2003 to 2008. *Diabetic medicine* **31,** 666–673 (2014) (cit. on p. 3).

25. Gheith, O., Farouk, N., Nampoory, N., Halim, M. A. & Al-Otaibi, T. Diabetic kidney disease: world wide difference of prevalence and risk factors. *Journal of nephropharmacology* **5,** 49 (2016) (cit. on p. 3).

26. Lin, C.-C. *et al.* Time trend analysis of the prevalence and incidence of diagnosed type 2 diabetes among adults in Taiwan from 2000 to 2007: a population-based study. *BMC public health* **13,** 1–10 (2013) (cit. on p. 3).

27. Virtanen, P. *et al.* SciPy 1.0: Fundamental Algorithms for Scientific Computing in Python. *Nature Methods* **17,** 261–272 (2020) (cit. on p. 5).
